# Supplementary material for: LC-MS/MS metabolomics-facilitated identification of the active compounds responsible for anti-allergic activity of the ethanol extract of Xenostegia tridentata
Source: PLoS One. 2022 Apr 15;17(4):e0265505. doi: 10.1371/journal.pone.0265505 (PMC9012362; doi:10.1371/journal.pone.0265505)

S1 Appendix. LC-MS Metabolite profiling and Comparison of MS and MS/MS data of the feature obtained from metabolite profiling to isolated compounds

1. LC-MS Metabolite profiling

Chromatographic profiles of 5 samples (F1: crude EtOH, F2: EtOH/Hex, F3: EtOH/EtOAc, F4: EtOH/BuOH and F5: EtOH/H_2O) analyzed in ESI positive mode and ESI negative mode.

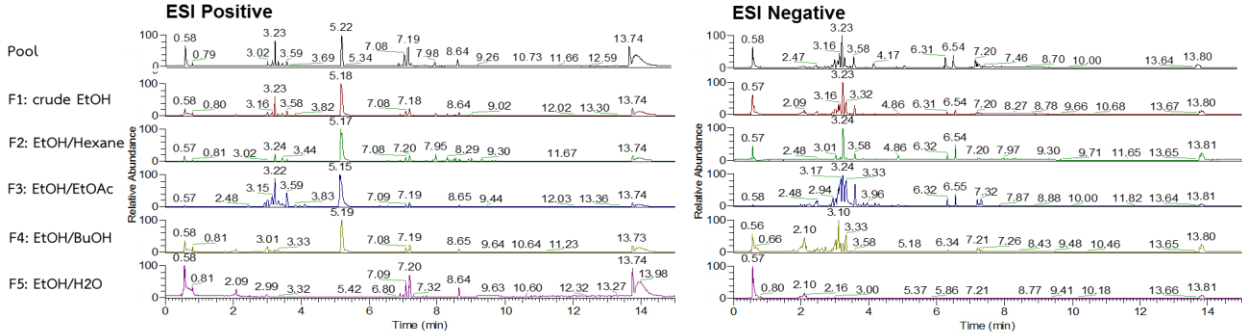

2. Comparison of MS and MS/MS data of the feature obtained from metabolite profiling to isolated compounds

Compared Peaks	Positive						Negative					
	$[M+H]^+$	RT (min)	ΔRT (min)	MS1	ΔMS1 (ppm)	Mass error (ppm)	$[M-H]^-$	RT (min)	ΔRT (min)	MS1	ΔMS1 (ppm)	Mass error (ppm)
F3: EtOH/EtOAc	517.1341	3.17	0.01	517.1340	2.32	-0.19	515.1195	3.15	0.01	515.1201	0.00	1.16
Purified Peak 1	517.1341	3.16		517.1328		-2.51	515.1195	3.16		515.1201		1.16
F3: EtOH/EtOAc	449.1078	3.02	0.00	449.1080	2.67	0.45	447.0933	3.02	0.01	447.0938	0.45	1.12
Purified Peak 2	449.1078	3.02		449.1068		-2.23	447.0933	3.03		447.0936		0.67
F3: EtOH/EtOAc	449.1078	3.23	0.02	449.1080	2.89	0.45	447.0933	3.23	0.02	447.0937	0.89	0.89
Purified Peak 3	449.1078	3.21		449.1067		-2.45	447.0933	3.21		447.0937		0.89
F3: EtOH/EtOAc	433.1129	3.60	0.02	433.1129	2.77	0.00	431.0984	3.59	0.01	431.0988	0.46	0.93
Purified Peak 4	433.1129	3.58		433.1117		-2.77	431.0984	3.58		431.0986		0.46

Feature: 516.12723_3.168

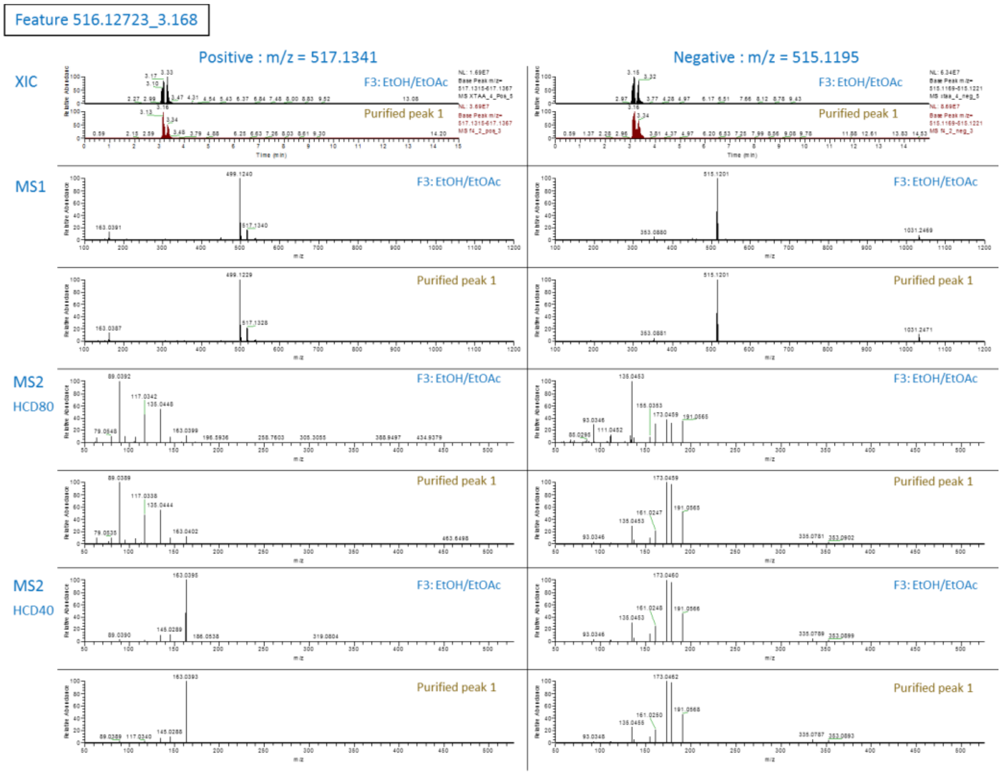

Feature: 448.10082_3.034

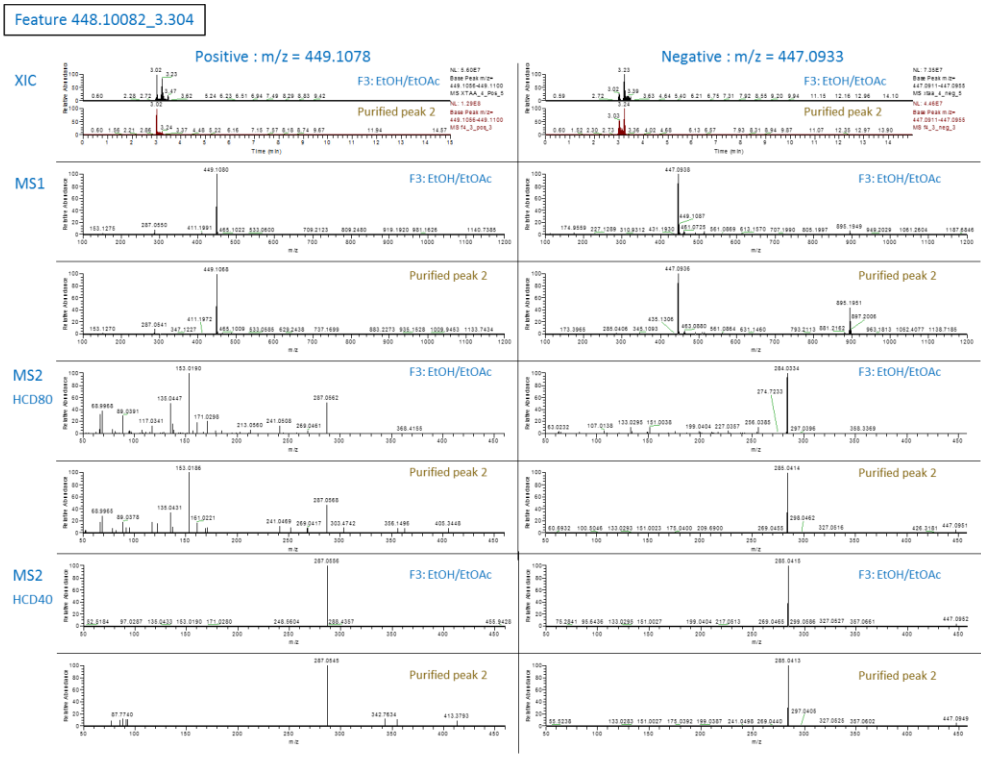

Feature: 448.10108_3.251

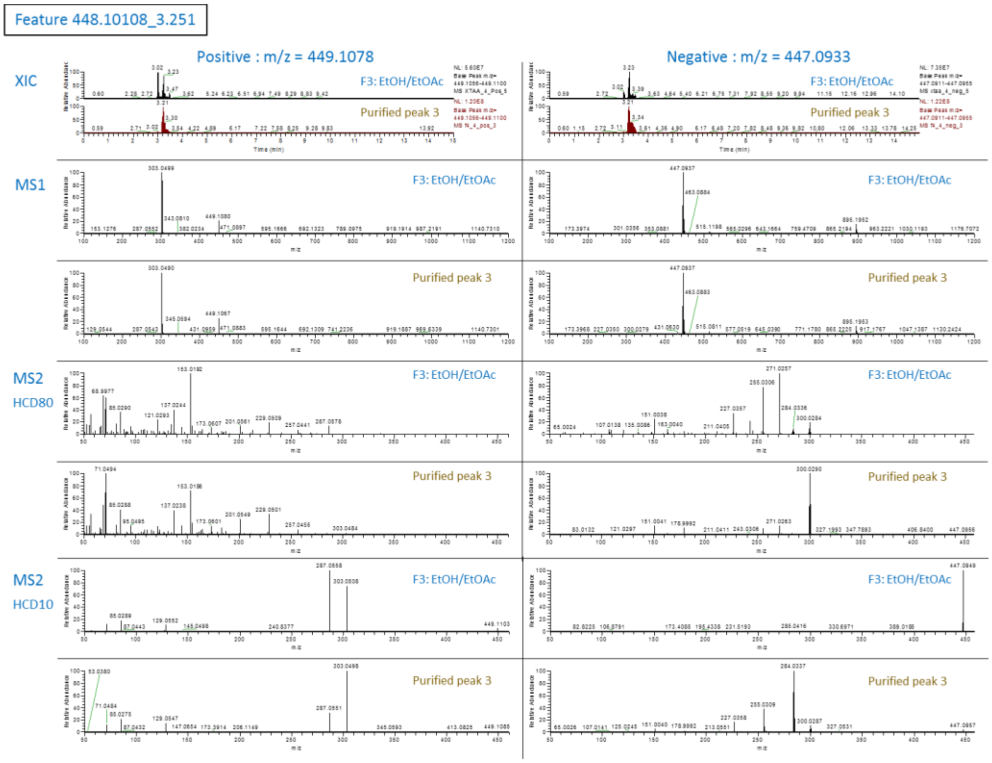

Feature: 432.10612_3.604

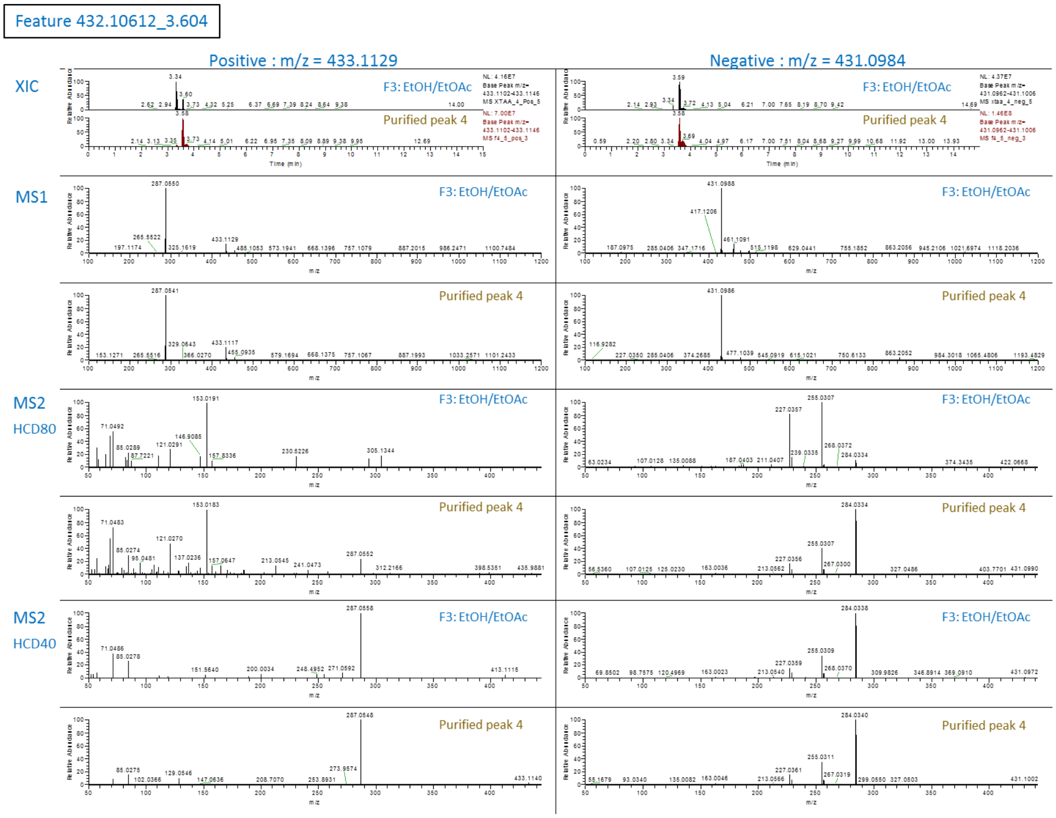

Supplement: S1 Appendix — (PDF) [file pone.0265505.s001.pdf]
