# Supplementary material for: LC-MS/MS metabolomics-facilitated identification of the active compounds responsible for anti-allergic activity of the ethanol extract of Xenostegia tridentata
Source: PLoS One. 2022 Apr 15;17(4):e0265505. doi: 10.1371/journal.pone.0265505 (PMC9012362; doi:10.1371/journal.pone.0265505)
Supplement: S2 Appendix — (PDF) [file pone.0265505.s002.pdf]

S2 Appendix. NMR spectra of the isolated compounds

1. Comparison of NMR spectra of the isolated compounds to literatures

3,5-dicaffeoylquinic acid

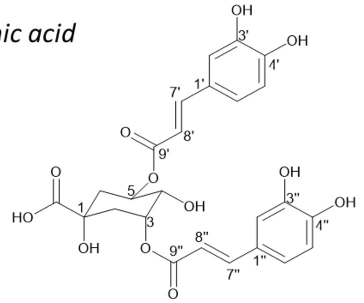

position	δ_H (*J* in Hz)		Δ (ppm)
	reference	experiment	
2	2.04-2.34 (2H, m)	2.11-2.37 (2H, m)	0.05
3	5.34-5.46 (1H, m)	5.35 – 5.49 (1H, m)	0.02
4	3.97 (1H, dd, 7.5,2.7)	3.99 (1H, dd, 7.5, 3.3)	0.02
5	5.34-5.46 (1H, m)	5.35 – 5.49 (1H, m)	0.02
6	2.04-2.34 (2H, m)	2.11-2.37 (2H, m)	0.05
2'/2''	7.07 (2H, s)	7.07 (2H, d, 2.0)	0
5'/5''	6.79/6.77 (2H, d, 8.3)	6.80/6.77 (2H, d, 1.2)	0.005
6'/6''	6.97/6.96 (2H, dd, 8.3, 2.2)	6.98/6.95 (2H, dd, 3.8, 2.1)	0
7'/7''	7.62/7.59 (2H, d, 16)	7.63/7.57 (2H, d, 12.2)	-0.005
8'/8''	6.36/6.27 (2H, d, 16)	6.35/6.27 (2H, d, 15.9)	-0.005

position	δ_C		Δ (ppm)
	reference	experiment	
1	74.8	74.7	-0.1
2	36.1	35.9	-0.2
3	72.6	72.5	-0.1
4	70.7	70.6	-0.1
5	72.1	72	-0.1
6	37.7	37.6	-0.1
1'/1''	127.9/127.8	127.9/127.8	0
2'/2''	115.6/115.2	115.5/115.2	-0.05
3'/3''	146.8/146.8	146.7/146.7	-0.1
4'/4''	149.6/149.5	149.5/149.4	-0.1
5'/5''	116.4/116.4	116.4/116.4	0
6'/6''	123.1/123.0	123.1/123.0	0
7'/7''	147.3/147.0	147.3/147.0	0
8'/8''	115.1/115.1	115.1/115.1	0
9'/9''	168.9/168.4	168.9/168.4	0
COO	177.6	177.3	-0.3

<u>*Reference*</u>*:* Chen, J. *et al.* Caffeoylquinic acid derivatives isolated from the aerial parts of *Gynura divaricata* and their yeast α-glucosidase and PTP1B inhibitory activity. *Fitoterapia* **99**, 1–6 (2014).

luteolin-7-O-glucoside

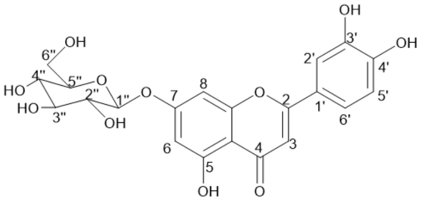

position	δ_H (*J* in Hz)		Δ (ppm)
	reference	experiment	
3	6.73 (1H, s)	6.75 (1H, s)	0.02
6	6.46 (1H, d, 2.0)	6.44 (1H, d, 2.1)	-0.02
8	6.81 (1H, d, 2.0)	6.78 (1H, d, 2.1)	-0.03
2'	7.43 (1H, d, 2.0)	7.42 (1H, d, 2.3)	-0.01
5'	6.94 (1H, d, 8.2)	6.90 (1H, d, 8.3)	-0.04
6'	7.47 (1H, dd, 8.2, 2.0)	7.45 (1H, dd, 8.3, 2.3)	-0.02
1''	5.07 (1H, d, 7.5)	5.08 (1H, d, 7.1)	0.01
Glc-OH	4.62∼5.34 (4H, m)		
Glc-H	3.20∼3.75 (6H, m)		

position	δ_C		Δ (ppm)
	reference	experiment	
2	164.9	164.5	-0.4
3	103.7	103.2	-0.5
4	181.8	181.9	0.1
5	161.2	161.1	-0.1
6	100	99.5	-0.5
7	163.4	162.9	-0.5
8	95.4	94.7	-0.7
9	157.4	156.9	-0.5
10	105.8	105.3	-0.5
1'	121.9	121.4	-0.5
2'	113.9	113.6	-0.3
3'	146	145.8	-0.2
4'	150.1	149.9	-0.2
5'	116.4	116	-0.4
6'	119.7	119.2	-0.5
1''	100.4	99.9	-0.5
2''	73.4	73.1	-0.3
3''	77.5	77.2	-0.3
4''	70	69.5	-0.5
5''	76.6	76.4	-0.2
6''	61	60.6	-0.4

<u>*Reference*</u>*:* Ma, Y. M., Zhang, Z. W. & Feng, C. L. Flavonoids of *Broussonetia papyrifera*. *Chem. Nat. Compd.* **45**, 881–882 (2009).

quercetin-3-O-rhamnoside

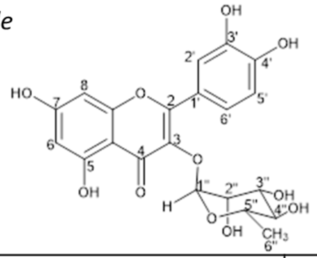

position	δ_H (*J* in Hz)		Δ (ppm)
	reference	experiment	
6	6.20 (1H, d, 1.3)	6.19 (1H, d, 2.1)	-0.01
8	6.36 (1H, d, 0.5)	6.36 (1H, d, 2.1)	0
2'	7.32 (1H, d, 2.0)	7.34 (1H, d, 2.1)	0.02
5'	6.90 (1H, d, 8.2)	6.91 (1H, d, 8.3)	0.01
6'	7.29 (1H, dd, 1.9, 8.3)	7.30 (1H, dd, 2.1, 8.3)	0.01
1''	5.35 (1H, d, 1.5)	5.35 (1H, d, 1.6)	0
2''	4.21 (1H, dd, 1.6, 3.2)	4.23 (1H, dd, 1.7, 3.4)	0.02
3''	3.73 (1H, dd, 3.3,9.2)	3.76 (1H, dd, 3.4, 9.2)	0.03
4''	3.40 (1H, m)	3.42(1H, m)	0.02
5''	3.33 (1H, m)	3.34 (1H, m)	0.01
6''	0.93 (3H, d, 6.0)	0.94 (2H, d, 5.9)	0.01

position	δ_C		Δ (ppm)
	reference	experiment	
2	159.5	159.3	-0.2
3	136.4	136.2	-0.2
4	179.8	179.6	-0.2
5	163.4	163.1	-0.3
6	100.1	99.8	-0.3
7	166.2	165.8	-0.4
8	94.9	94.7	-0.2
9	158.7	158.5	-0.2
10	106	105.9	-0.1
1'	123	122.9	-0.1
2'	117.1	117	-0.1
3'	146.6	149.7	3.1
4'	145	146.4	1.4
5'	116.6	116.3	-0.3
6'	123.1	122.9	-0.2
1''	103.7	103.5	-0.2
2''	72.1	71.9	-0.2
3''	72.3	72.1	-0.2
4''	72.2	72	-0.2
5''	73.5	73.2	-0.3
6''	17.9	17.6	-0.3

<u>*Reference*</u>*:* Baiseitova, A., Jenis, J., Kim, J. Y., Li, Z. P. & Park, K. H. Phytochemical analysis of aerial part of *Ikonnikovia kaufmanniana* and their protection of DNA damage. *Nat. Prod. Res.* **35**, 880–883 (2021).

Kaempferol-3-O-rhamnoside

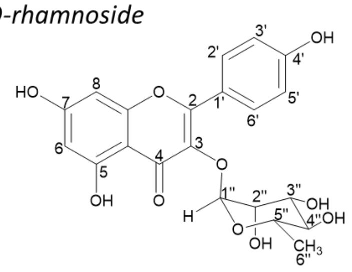

position	δ_H (*J* in Hz)		Δ (ppm)
	reference	experiment	
6	5.96 (1H, d, 2)	6.20 (1H, d, 2.1)	0.24
8	6.08 (1H, d, 2)	6.38 (1H, d, 2.1)	0.3
2',6'	7.68 (2H, d, 7.0)	7.76 (2H, d, 8.8)	0.08
3',5'	6.84 (2H, d, 7.0)	6.94 (2H, d, 8.8)	0.1
1''	5.29 (1H, d, 1.4)	5.38 (1H, d, 1.7)	0.09
2''	4.20 (1H, dd, J, 3.2, 1.6)	4.22 (1H, dd, 3.4, 1.8)	0.02
3'', 4'', 5''	3.18-3.75 (3H, m)	3.34-3.86 (3H, m)	0.135
6''	0.90 (3H, d, J, 5.8)	0.92 (3H, d, 5.9)	0.02

position	δ_C		Δ (ppm)
	reference	experiment	
2	159.5	159.3	-0.2
3	135.1	136.2	1.1
4	178.6	179.6	1
5	163.1	163.2	0.1
6	100.4	99.8	-0.6
7	166.4	165.8	-0.6
8	95.2	94.8	-0.4
9	158.9	158.5	-0.4
10	105	106	1
1'	123	122.6	-0.4
2', 6'	132.3	131.9	-0.4
3', 5'	116	116.5	0.5
4'	161.5	161.5	0
1''	103.5	103.5	0
2''	72.2	72.1	-0.1
3''	72	72	0
4''	73.3	73.2	-0.1
5''	71.9	71.9	0
6''	17.6	17.6	0

<u>*Reference*</u>*:* Wu, T.-S. & Chan, Y.-Y. Constituents of Leaves of *Uncaria hirsuta* Haviland. *J. Chinese Chem. Soc.* **41**, 209–212 (1994).

Kaouadji, M. Acylated and non-acylated kaempferol monoglycosides from *Platanus acerifolia* buds. *Phytochemistry* **29**, 2295–2297 (1990).

2. NMR spectra of the isolated compounds

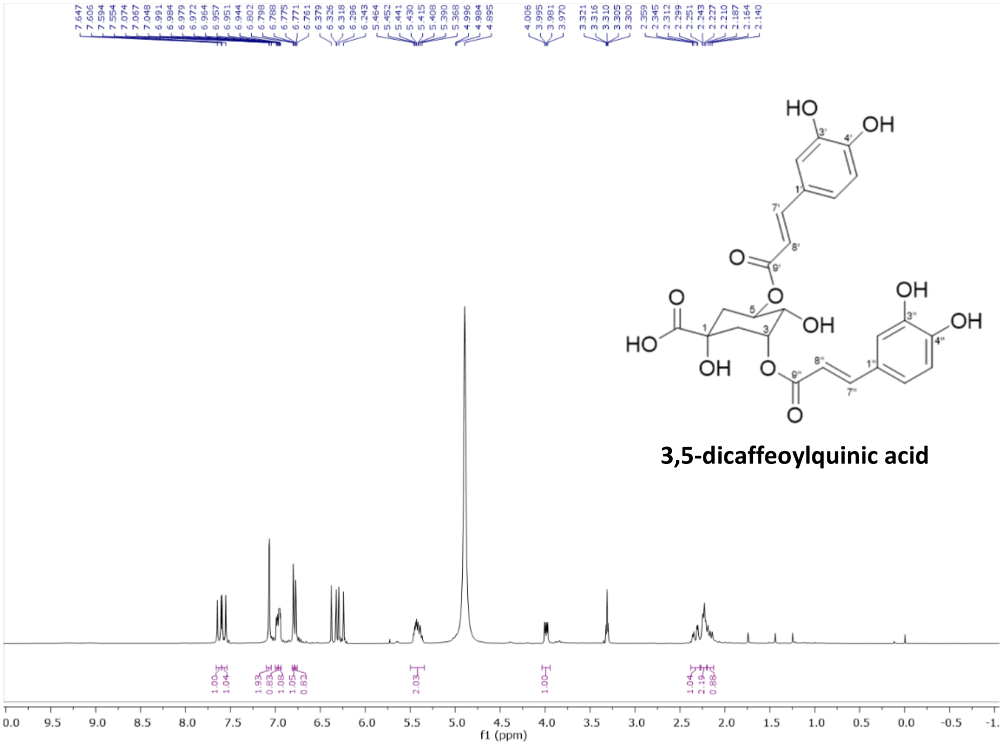

Figure 2-1. ^{1}H-NMR spectrum of 3,5-dicaffeoylquinic acid in CD_3OD

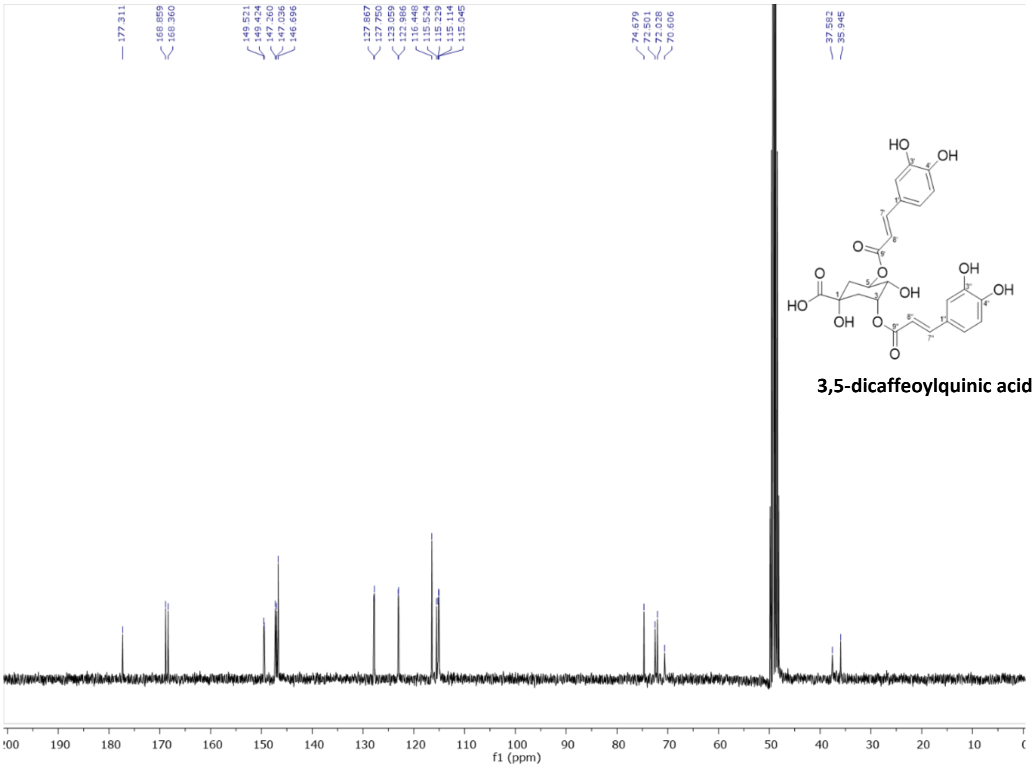

Figure 2-2. ^{13}C-NMR spectrum of 3,5-dicaffeoylquinic acid in CD_3OD

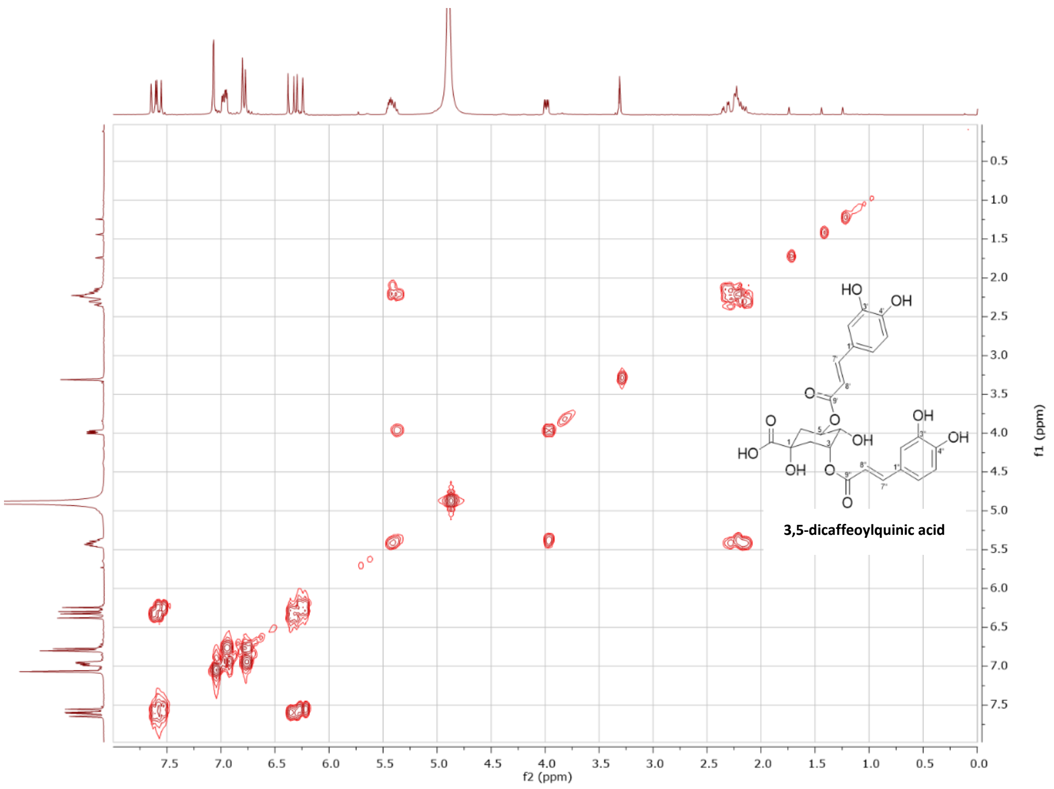

Figure 2-3. COSY spectrum of 3,5-dicaffeoylquinic acid in CD_3OD

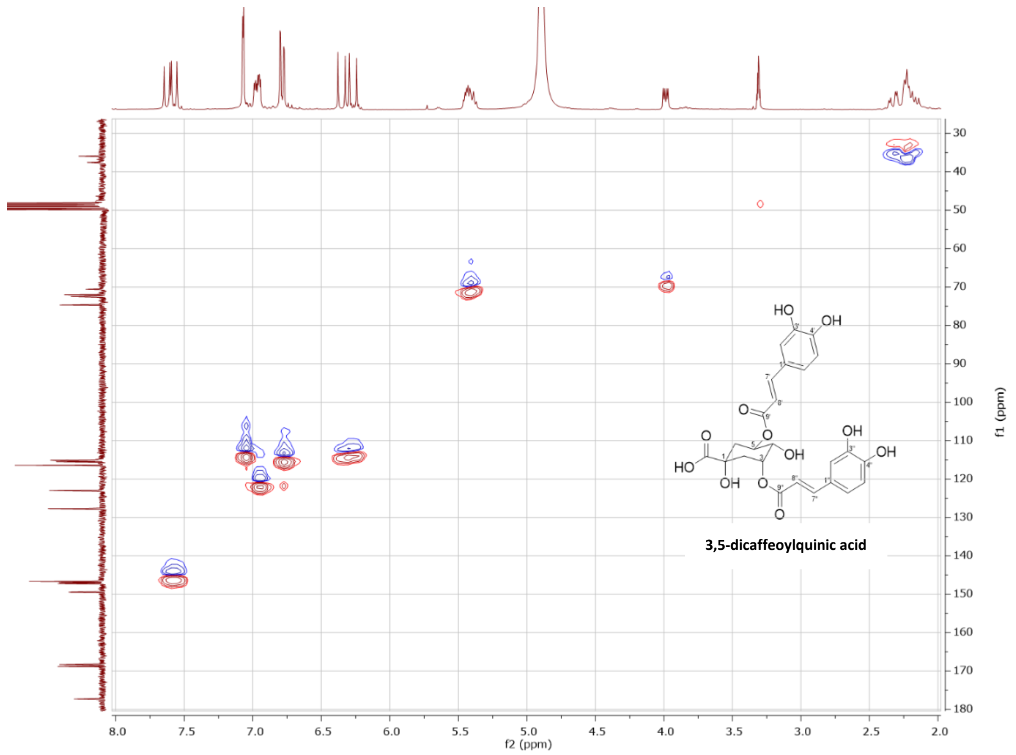

Figure 2-4. HSQC spectrum of 3,5-dicaffeoylquinic acid in CD_3OD

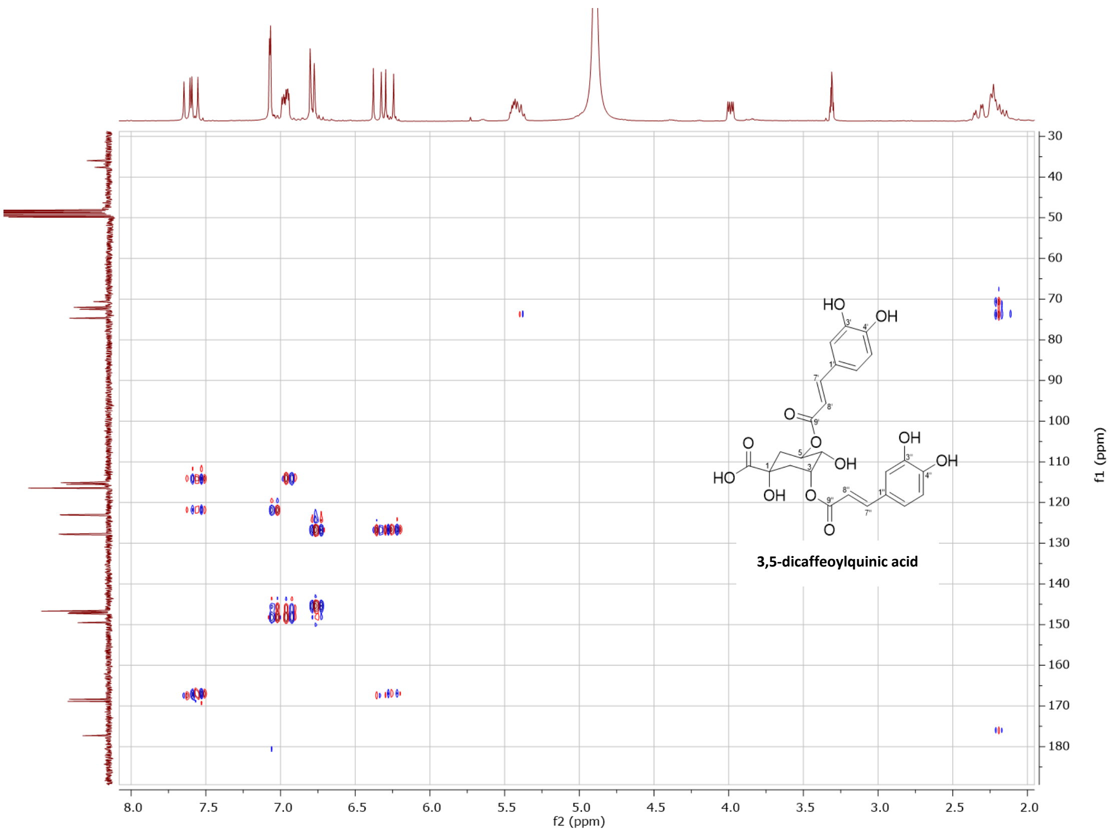

Figure 2-5. HMBC spectrum of 3,5-dicaffeoylquinic acid in CD_3OD

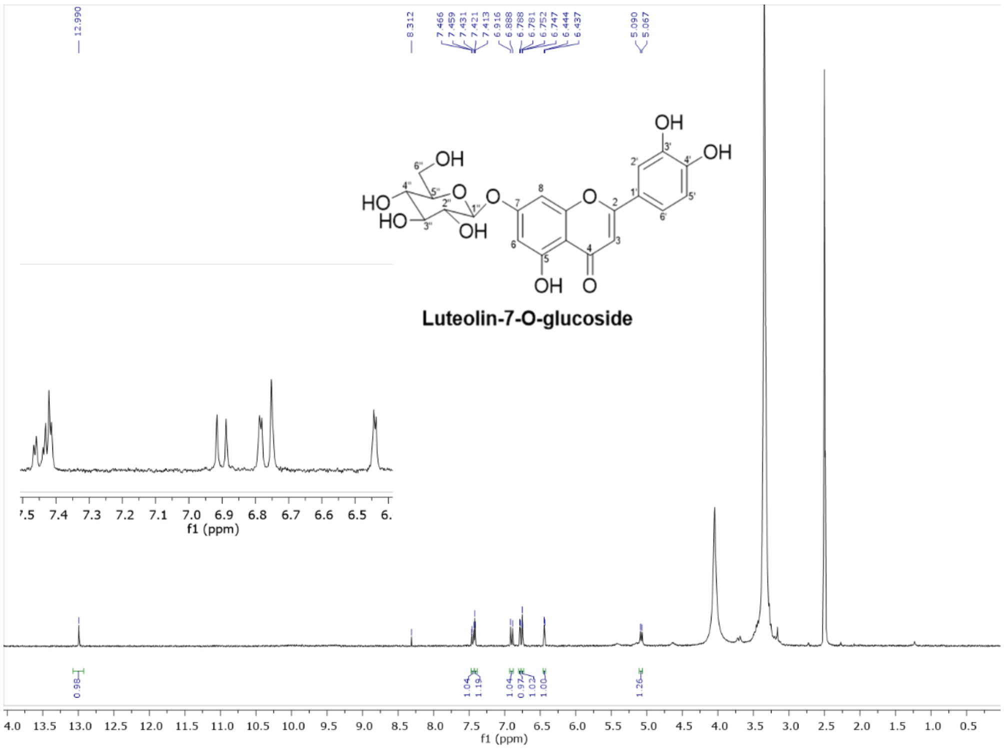

Figure 2-6. ^{1}H-NMR spectrum of luteolin-7-O-glucoside in DMSO-d_6

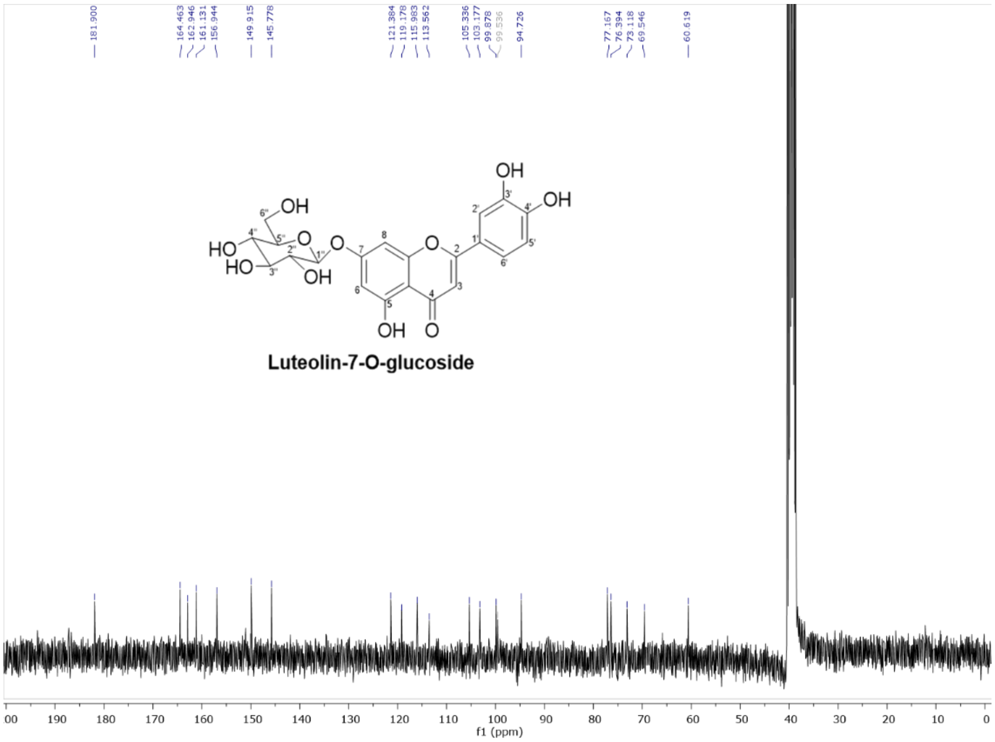

Figure 2-7. ^{13}C-NMR spectrum of luteolin-7-O-glucoside in DMSO-d_6

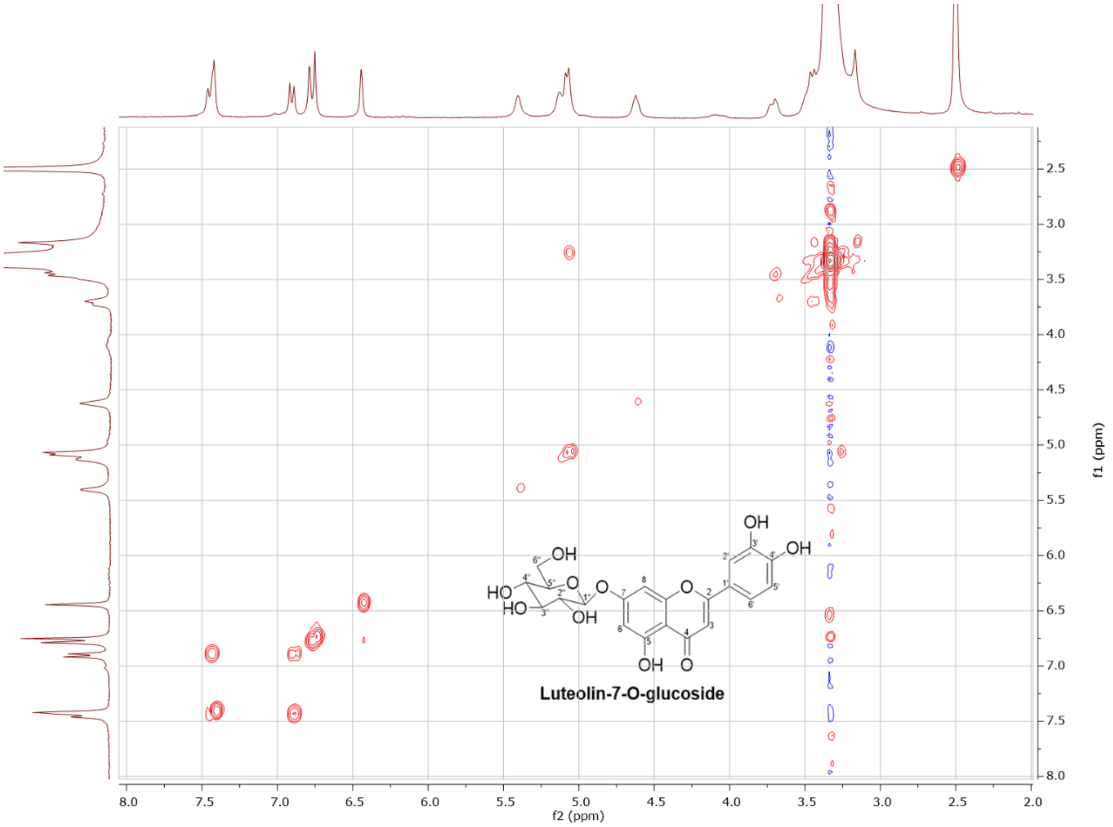

Figure 2-8. COSY spectrum of luteolin-7-O-glucoside in DMSO-d_6

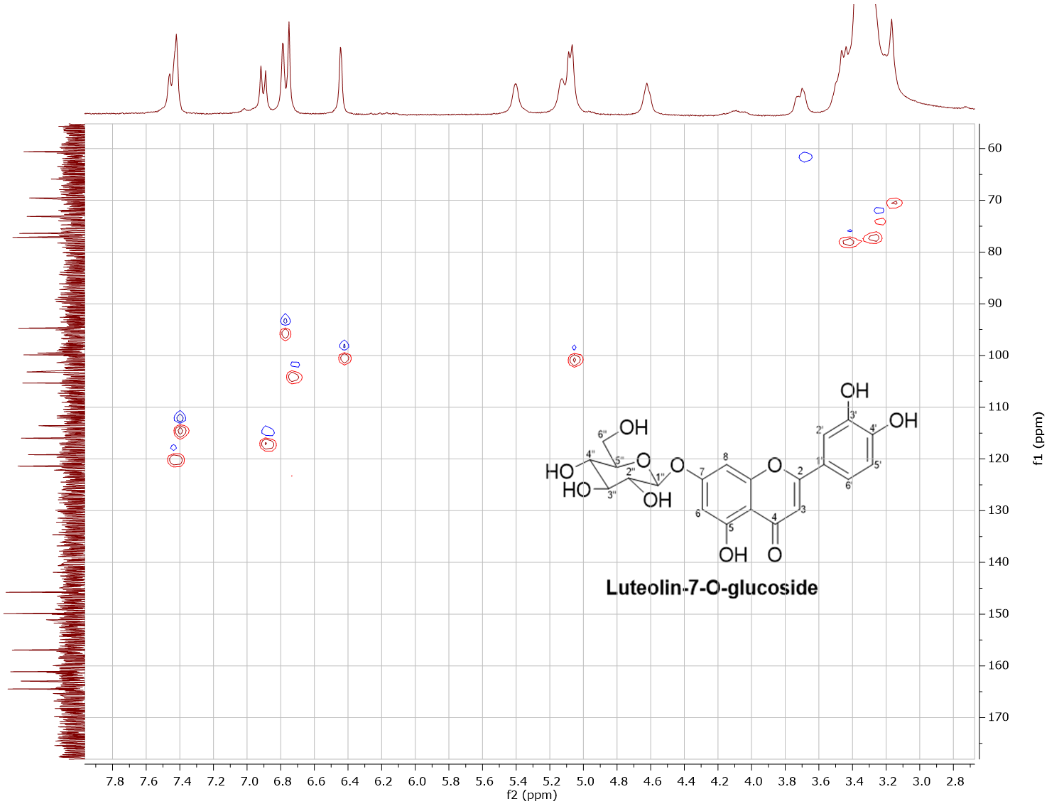

Figure 2-9. HSQC spectrum of luteolin-7-O-glucoside in DMSO-d6

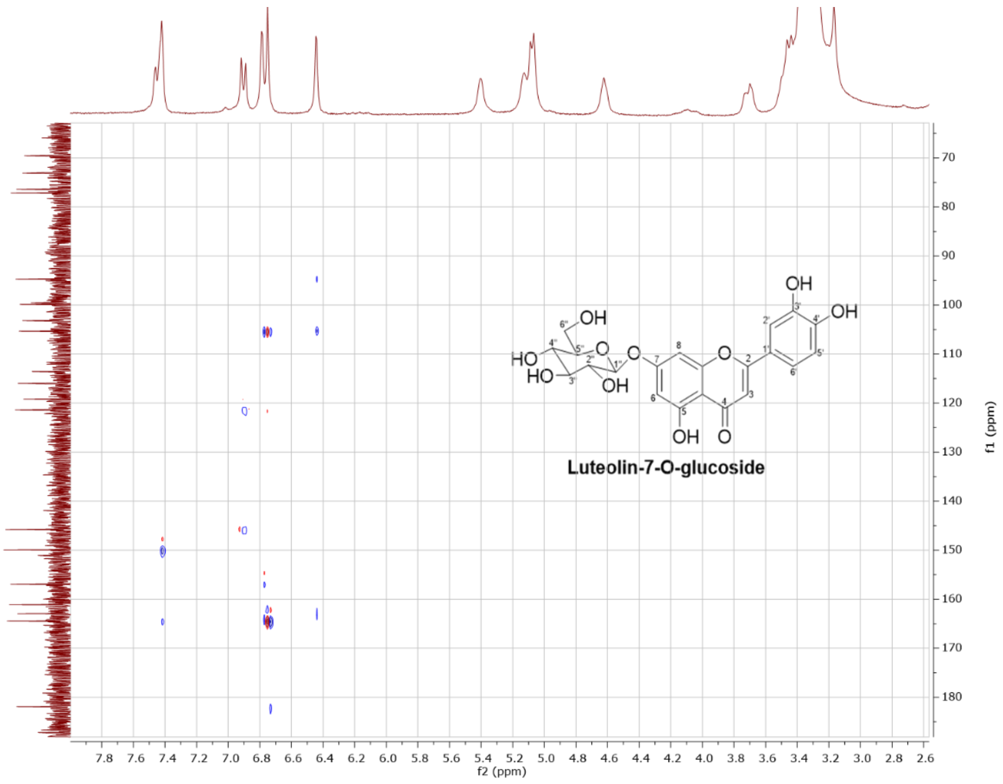

Figure 2-10. HMBC spectrum of luteolin-7-O-glucoside in DMSO-d6

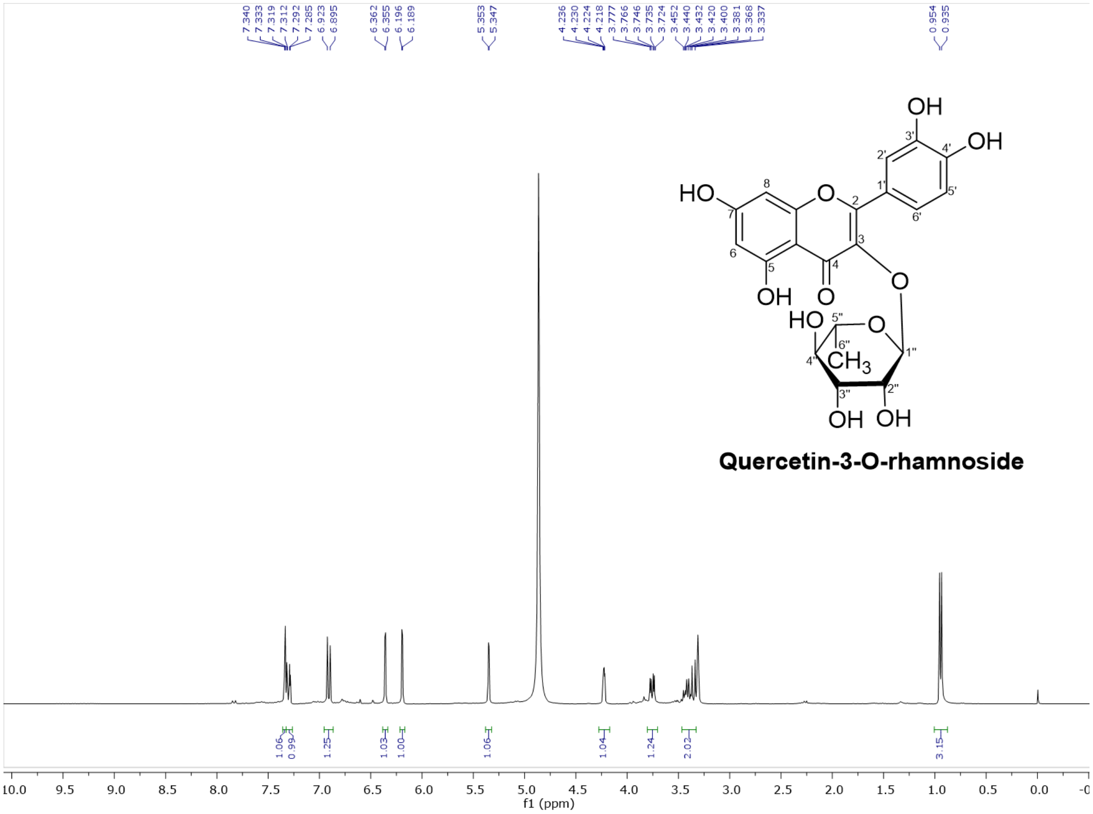

Figure 2-11. ^{1}H-NMR spectrum of quercetin-3-O-rhamnoside in CD_3OD

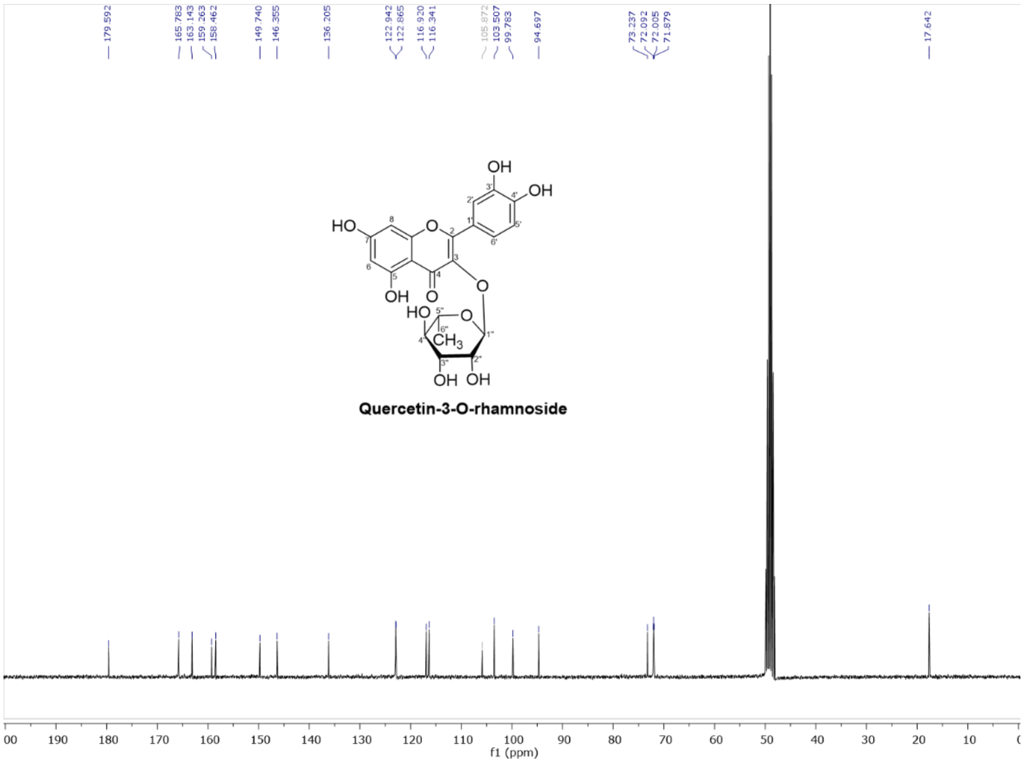

Figure 2-12. ^{13}C-NMR spectrum of quercetin-3-O-rhamnoside in CD_3OD

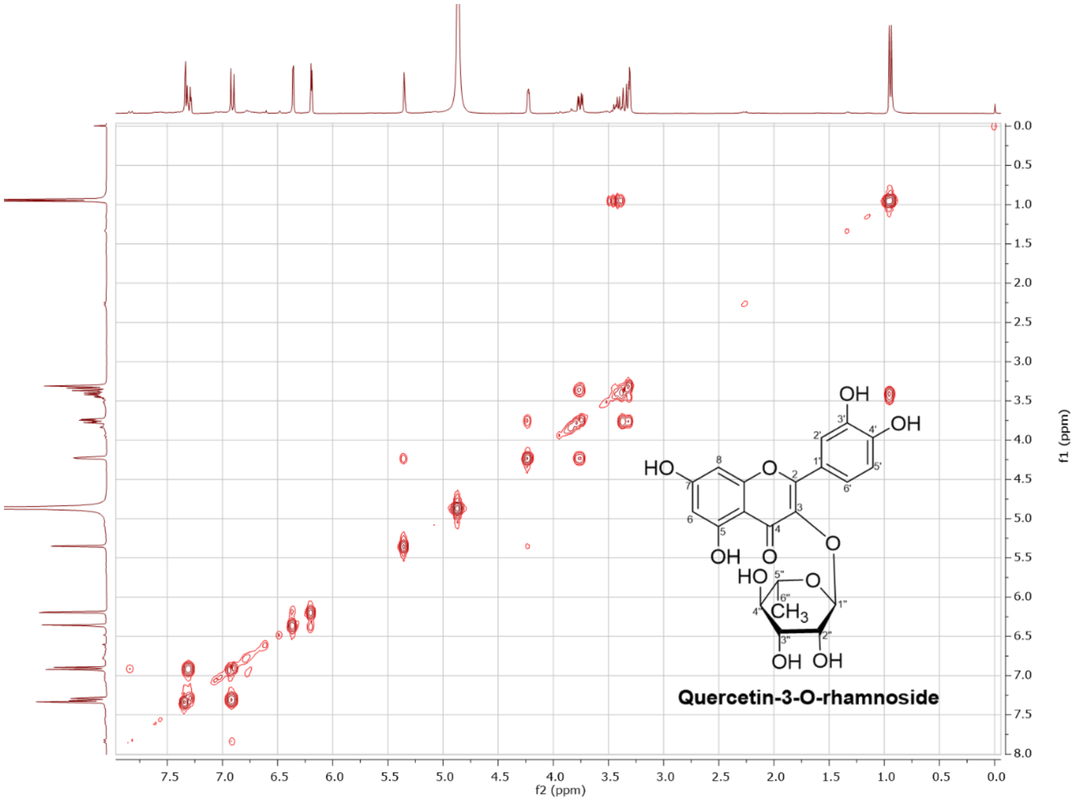

Figure 2-13. COSY spectrum of quercetin-3-O-rhamnoside in CD_3OD

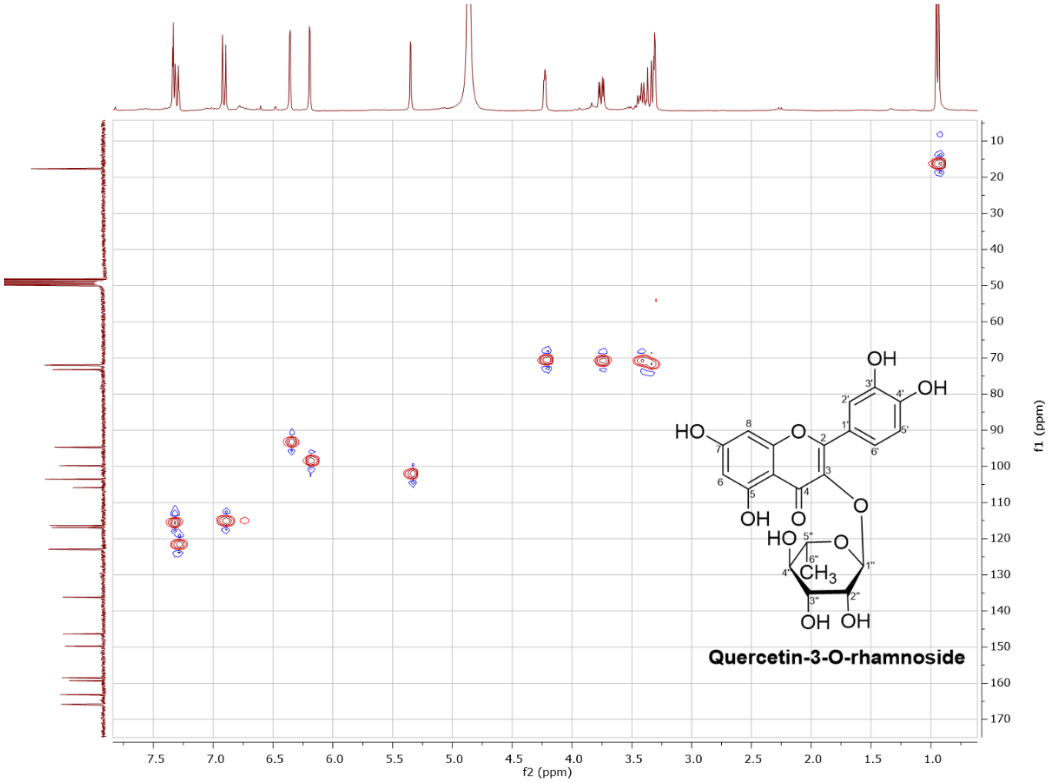

Figure 2-14. HSQC spectrum of quercetin-3-O-rhamnoside in CD_3OD

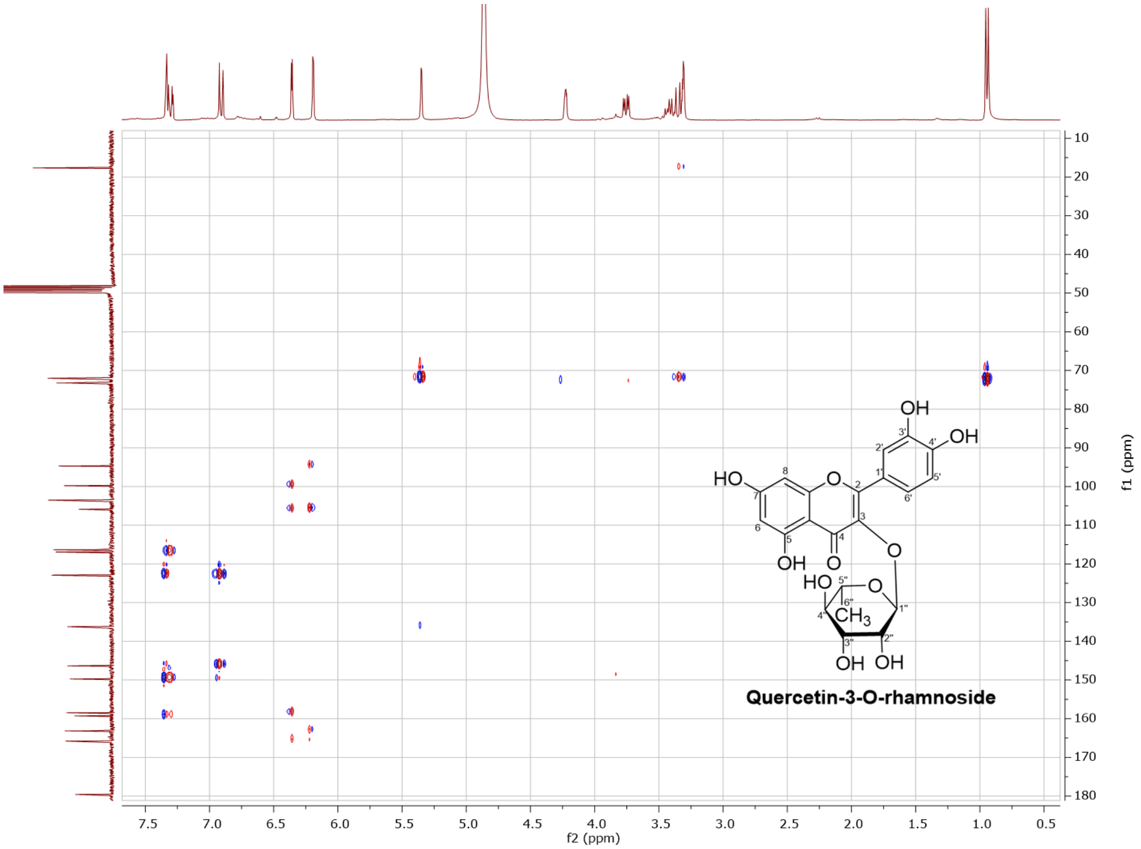

Figure 2-15. HMBC spectrum of quercetin-3-O-rhamnoside in CD_3OD

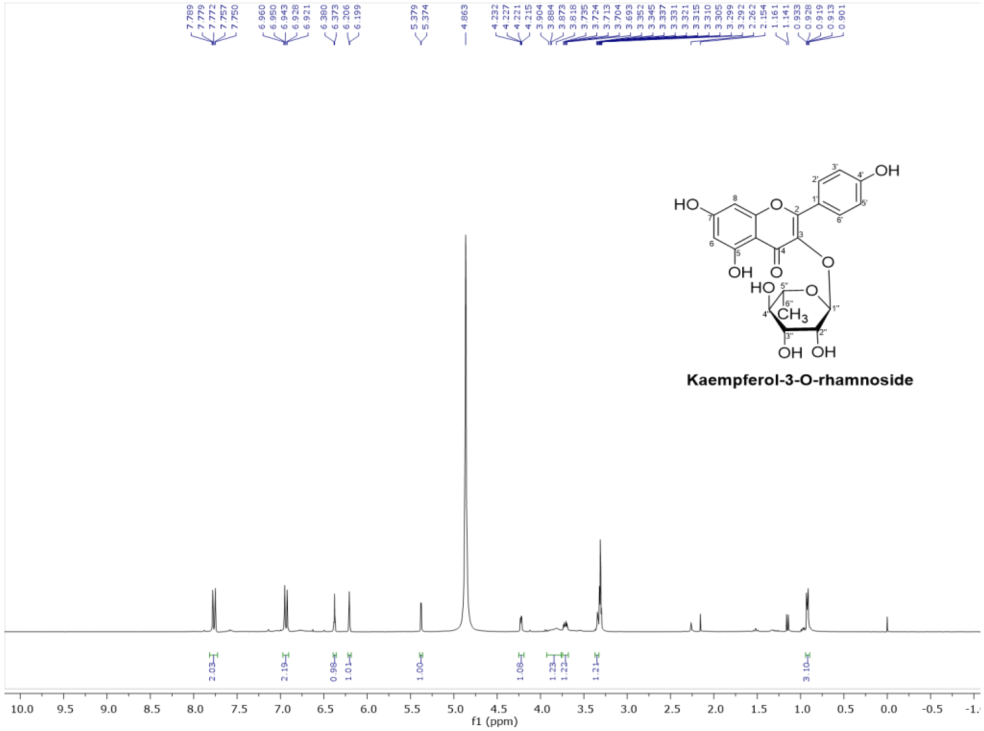

Figure 2-16. ^1H-NMR spectrum of Kaempferol-3-O-rhamnoside in CD_3OD

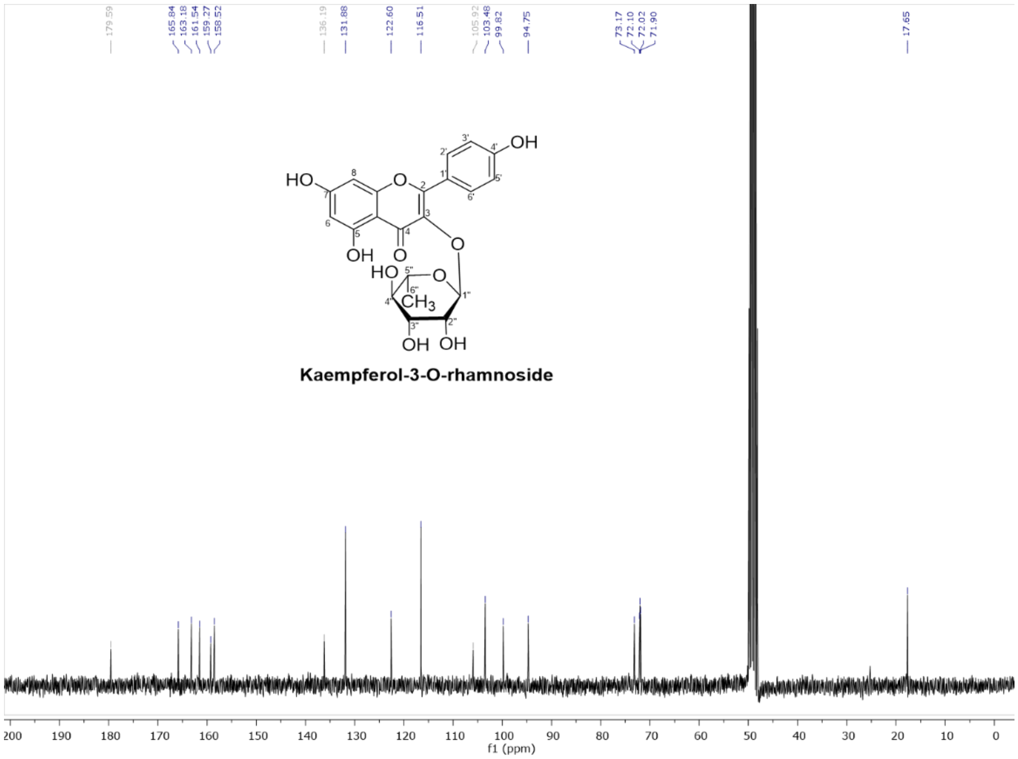

Figure 2-17. ^{13}C-NMR spectrum of Kaempferol-3-O-rhamnoside in CD_3OD

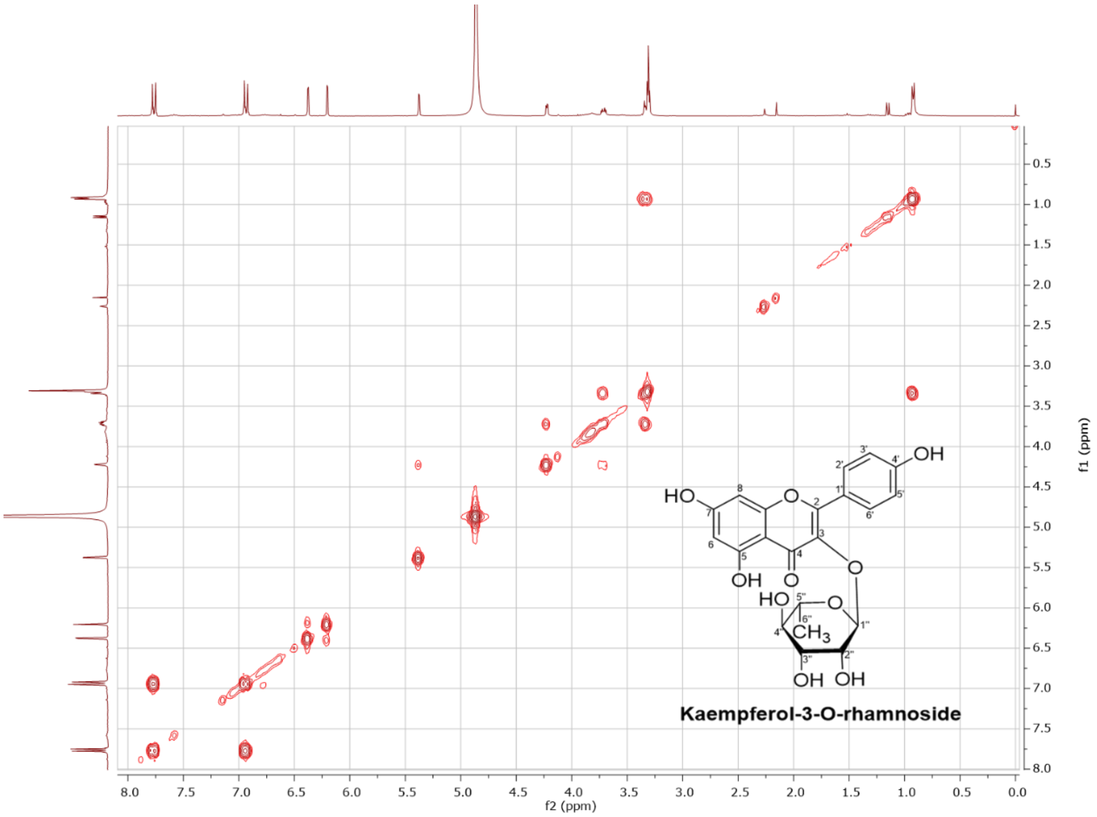

Figure 2-18. COSY spectrum of Kaempferol-3-O-rhamnoside in CD_3OD

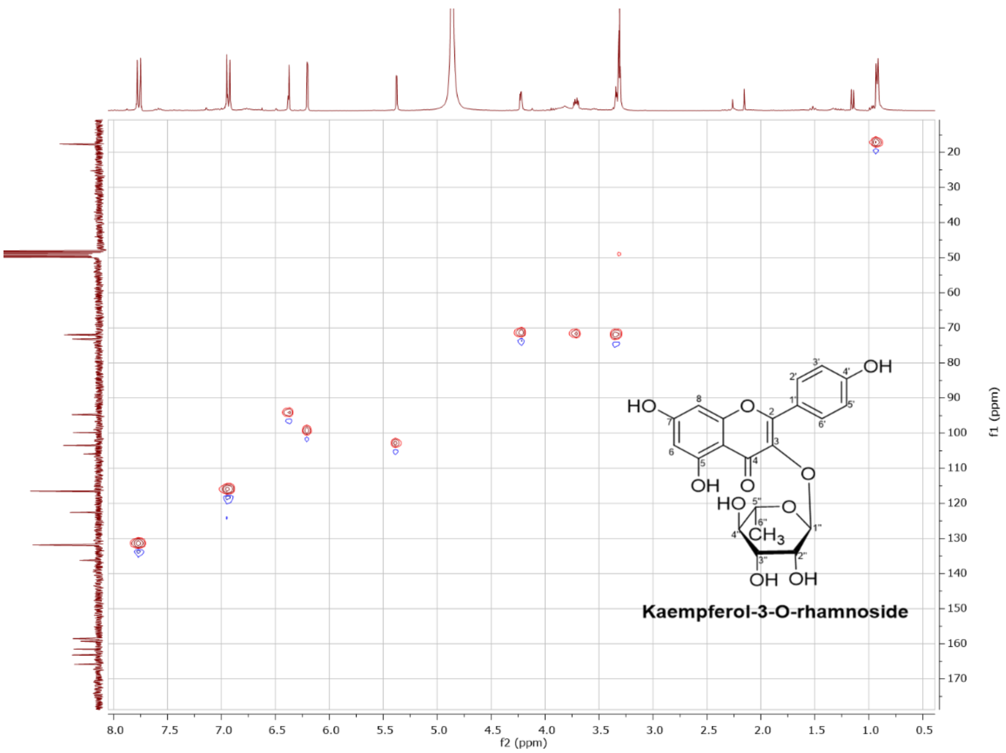

Figure 2-19. HSQC spectrum of Kaempferol-3-O-rhamnoside in CD_3OD

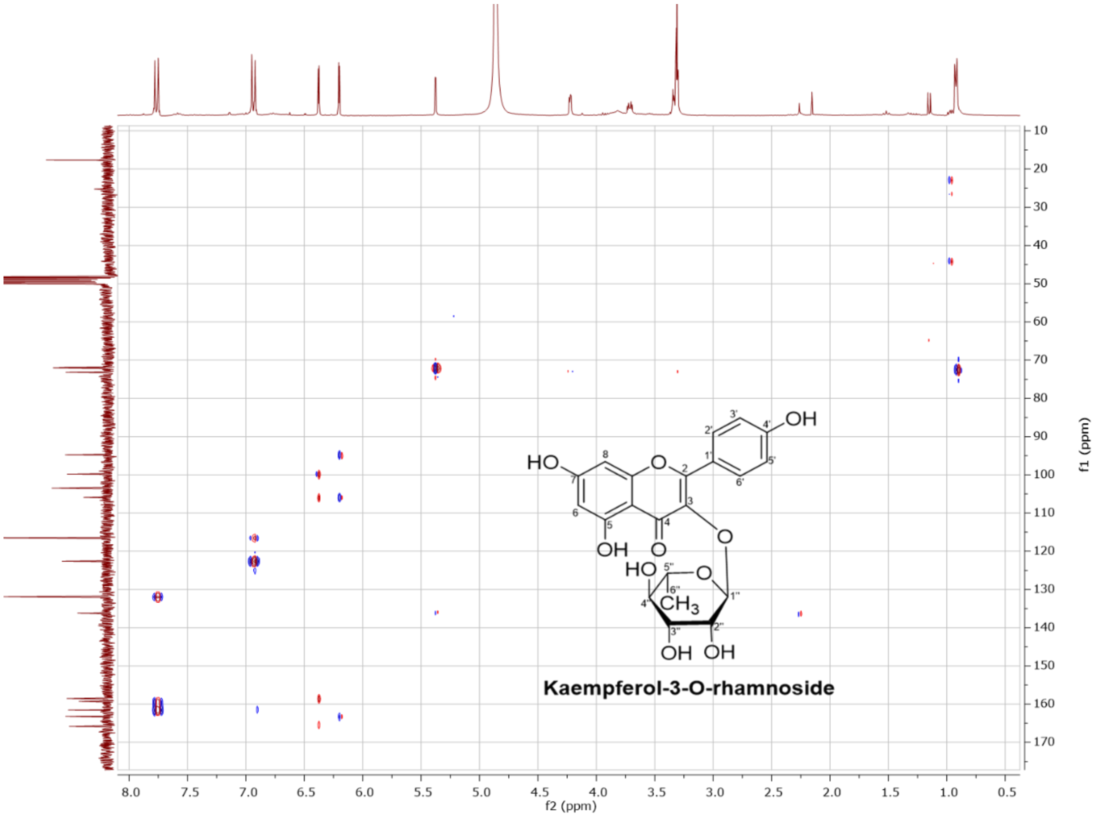

Figure 2-20. HMBC spectrum of Kaempferol-3-O-rhamnoside in CD_3OD

3. HPLC chromatograms of the isolated compounds

3.1 3,5-dicaffeoylquinic acid

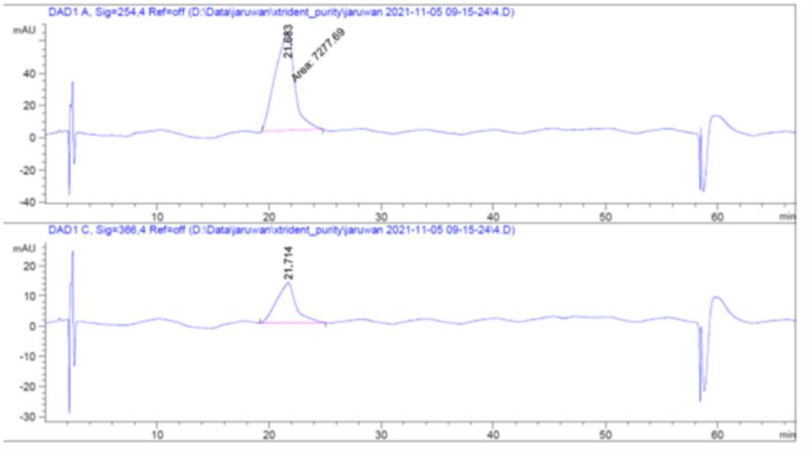

Signal 1: DAD1 A, Sig=254,4 Ref=off

Peak #	RetTime [min]	Type	Width [min]	Area [mAU*s]	Height [mAU]	Area %
1	21.683	MM	1.9970	7277.68945	60.73751	100.0000
Totals :				7277.68945	60.73751	

Signal 2: DAD1 C, Sig=366,4 Ref=off

Peak #	RetTime [min]	Type	Width [min]	Area [mAU*s]	Height [mAU]	Area %
1	21.714	BB	1.4561	1604.91162	13.27803	100.0000
Totals :				1604.91162	13.27803	

3.2 luteolin-7-O-glucoside

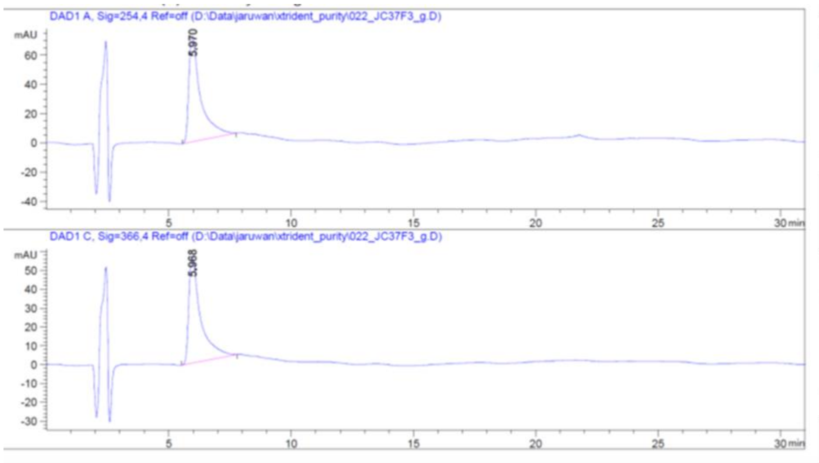

Signal 1: DAD1 A, Sig=254,4 Ref=off

Peak #	RetTime [min]	Type	Width [min]	Area [mAU*s]	Height [mAU]	Area %
1	5.970	BB	0.4821	2424.39087	71.77569	100.0000
Totals :				2424.39087	71.77569	

Signal 2: DAD1 C, Sig=366,4 Ref=off

Peak #	RetTime [min]	Type	Width [min]	Area [mAU*s]	Height [mAU]	Area %
1	5.968	BB	0.4866	1920.42749	56.21298	100.0000
Totals :				1920.42749	56.21298	

3.3 quercetin-3-O-rhamnoside

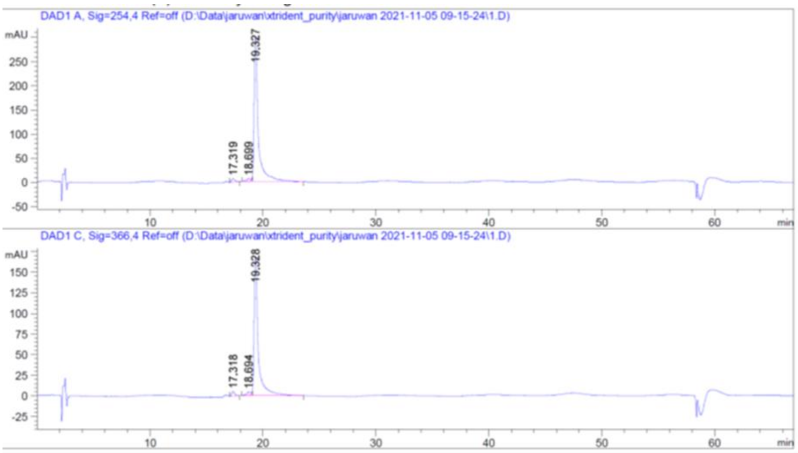

Signal 1: DAD1 A, Sig=254,4 Ref=off

Peak #	RetTime [min]	Type	Width [min]	Area [mAU*s]	Height [mAU]	Area %
1	17.319	BB	0.2633	138.09891	7.41467	1.7050
2	18.699	BV E	0.3372	142.05417	5.53922	1.7538
3	19.327	VB R	0.3717	7819.50537	300.43076	96.5412

Signal 2: DAD1 C, Sig=366,4 Ref=off

Peak #	RetTime [min]	Type	Width [min]	Area [mAU*s]	Height [mAU]	Area %
1	17.318	BB	0.2580	103.88760	5.66264	2.2848
2	18.694	BV E	0.3144	103.75903	4.37941	2.2820
3	19.328	VB R	0.3691	4339.29346	168.14775	95.4333

3.4 kaempferol-3-O-rhamnoside

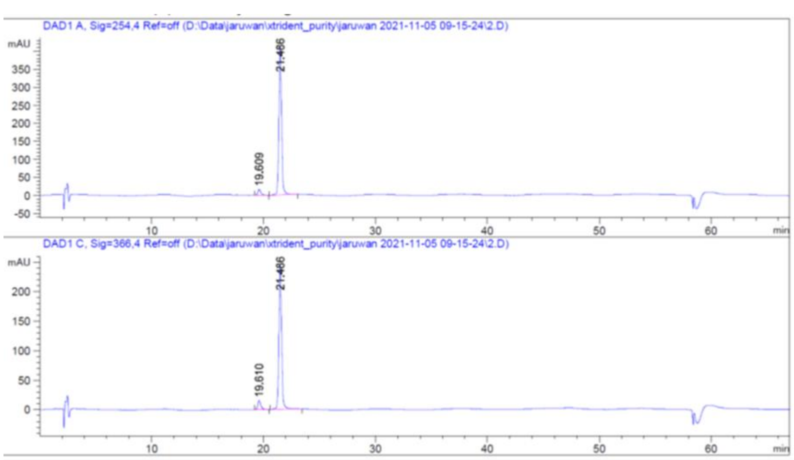

Signal 1: DAD1 A, Sig=254,4 Ref=off

Peak #	RetTime [min]	Type	Width [min]	Area [mAU*s]	Height [mAU]	Area %
1	19.609	BB	0.2855	317.31110	16.86901	4.3850
2	21.486	VB R	0.2623	6918.90869	413.10666	95.6150

Signal 2: DAD1 C, Sig=366,4 Ref=off

Peak #	RetTime [min]	Type	Width [min]	Area [mAU*s]	Height [mAU]	Area %
1	19.610	BB	0.2861	294.57797	15.61703	6.6367
2	21.486	BB	0.2647	4144.07324	245.77841	93.3633
Totals :				4438.65121	261.39544	
