# Supplementary material for: LC-MS/MS metabolomics-facilitated identification of the active compounds responsible for anti-allergic activity of the ethanol extract of Xenostegia tridentata
Source: PLoS One. 2022 Apr 15;17(4):e0265505. doi: 10.1371/journal.pone.0265505 (PMC9012362; doi:10.1371/journal.pone.0265505)
Supplement: S1 Table — (PDF) [file pone.0265505.s003.pdf]

S3 Table. Extraction yields, β-hexosaminidase inhibitory activity of the extracts, cytotoxicity of the isolated compounds and previously reported anti-allergic activities of the isolated compounds

1. Extraction yields

Two batches of extraction (200g- and 100g-scales) were performed. The obtained yields are shown in the following table.

Experiment	Fractions	200g-scale		100g-scale	
		gram	%yield	gram	%yield
maceration	Plant powder	200		100	
	crude (EtOH)	26.29	13.1	14.72	14.7
solvent extraction	crude EtOH taken for partition	24.5		14.7	
	EtOH/Hex	11.2	45.7	7.5	51.0
	EtOH/EtOAc	0.9	3.7	0.6	4.1
	EtOH/BuOH	2.6	10.6	1.3	8.8
	EtOH/H2O	4.6	18.8	2.9	19.7
	total	19.3	78.8	12.3	83.7

After the extractions, the same fractions from the two batches were combined before moving forward to the experiments reported in the manuscript.

2. Inhibitory effects of *X. tridentata* extracts on β-hexosaminidase activity

sample ID	conc. (μg/mL)	% inhibition of β-hex activity
F1: Crude EtOH	500	3.6 ± 0.8
	1000	-2.2 ± 0.6
	2000	-7.2 ± 0.1
F2: EtOH/Hex	500	4.1 ± 0.7
	1000	0.8 ± 1.6
	2000	-3.5 ± 0.7
F3: EtOH/EtOAc	500	-18.5 ± 0.6
	1000	-46.1 ± 0.9
	2000	-89.5 ± 0.5
F4: EtOH/BuOH	500	2.1 ± 0.7
	1000	-5.8 ± 1.4
	2000	-12.5 ± 0.9
F5: EtOH/H2O	500	-4.8 ± 0.3
	1000	-20.7 ± 1.3
	2000	-35.3 ± 0.8
ketotifen fumarate	25	-12.4 ± 3.9
	50	-21.0 ± 1.1
	100	-31.0 ± 0.8

The extracts showed no inhibitory effect on β-hexosaminidase activity, assuring that the observed mast cell degranulation inhibitory activity was not because of the ability of extracts to inhibit β-hexosaminidase.

3. Cytotoxicity of the isolated compounds on RBL-2H3 cells

		%viability
3,5-dicaffeoylquinic acid	100 µM	108.5±2.6
	25 µM	105.7±1.1
luteolin-7-O-glucoside	100 µM	103.7±1.1
	25 µM	104.9±0.4
quercetin-3-O-rhamnoside	100 µM	112.7±9.6
	25 µM	105.7±1.7
kaempferol-3-O-rhamnoside	100 µM	101.1±4.1
	25 µM	103.6±0.3
ketotifen fumarate	100 µM	115.3±0.1
	25 µM	97.1±1.8

The cytotoxicity of the isolated compounds (up to 100 µM) was not observed.

Note:

In the manuscript, values from the degranulation assay were calculated according to the following equations.

β-hex released ratio = Abs (supernatant)/ [Abs (supernatant) + Abs (cell lysate)]

β-hexosaminidase release ratio (% of max) = [release ratio (sample)/release ratio (control)] x 100

So, the “max” was the vehicle control, meaning that the experiment was performed by

1. sensitizing the cells with IgE
2. treating with DMSO (instead of the extracts or the isolated compounds) in Siraganian buffer.
3. stimulating the cells with the antigen.

4. Previously reported anti-allergic activities of the isolated compounds

Table 4.1. Examples of previously reported anti-allergic activities of the isolated compounds.

compounds	activities	references
**3,5-dicaffeoylquinic acid**	"Glycosylated flavonoids, caffeoylquinic acid and 3,5-dicaffeoylquinic acid have been identified in the ethyl acetate partition from the crude ethanol extract of Tocoyena bullata (Rubiaceae) leaves. The fraction containing the mixture of flavonol rutin and a tetraglycosylated flavonoid showed 89.2% inhibition and the mixture of isoquercitrin and 3,5-dicaffeoylquinic acid showed 88.5% inhibition of mast cell degranulation. **These results demonstrated that the tetraglycosylated flavonoid, rutin, isoquercitrin and 3,5-dicaffeoylquinic acid were the most promising phenolics for inhibition of mast cell degranulation."**	Natural Product Research. 34(22), 3295-3298 (2020)
	Inhibition of β-hexosaminidase release (leukotriene C4/D4/E4 synthesis and TNF-a production) and PCA reaction and suppression of smooth muscle constriction induced by histamine and leukotriene D4	J. Clin. Biochem. Nutr. 67(1), 10–18 (2020)
**luteolin-7-O-glucoside (Cynaroside)**	inhibitory activity on histamine release from rat peritoneal mast cells induced by compound 48/80 (IC50 = 41.6 uM)	Biol. Pharm. Bull. 25(2), 256—259 (2002)
	inhibitory activity on IgE-mediated Anti-allergic activity on RBL-2H3 cell line (IC50 = 470 uM)	CMU.J.Nat.Sci.Special Issue on Agricultural & Natural Resources. 11(1), 343 (2012)
	"Flavonoid glycosides**, luteolin 7-O-glucoside and stechamone, and maltol isolated from S. chamaejasme also showed strong antiallergic effects in hapten-induced dermatitis-like skin lesions in murine models as main components**"	Natural Product Communications. 15(7), 1–7 (2020)
	"Stellera chamaejasme and **Its Main Compound Luteolin 7-O-Glucoside Alleviates Skin Lesions in Oxazolone- and 2,4-DinitrochlorobenzeneStimulated Murine Models of Atopic Dermatitis**"	Planta Med. 85(7), 583-590 (2019)
**quercetin-3-O-rhamnoside (quercitrin)**	"Quercitrin, but not rutin, inhibited OVA-induced contractions of sensitized trachea."	Medicine in Drug Discovery. 12, 100106 (2021)

**kaempferol-3-O-rhamnoside (Afzelin)**	"kaempferol-3-O-rhamnoside fully maintained its anti-inflammatory and anti-asthmatic effects compared with kaempferol in an asthma model mouse. Both kaempferol and kaempferol-3-O-rhamnoside significantly reduced the elevated inflammatory cell numbers in the bronchoalveolar lavage fluid (BALF). kaempferol and kaempferol-3-O-rhamnoside also significantly inhibited the increase in Th2 cytokines (IL-4, IL-5, and IL-13) and TNF-α protein levels through inhibition of the phosphorylation Akt and effectively suppressed eosinophilia in a mouse model of allergic asthma. The total immunoglobulin (Ig) E levels in the serum and BALF were also blocked by kaempferol and kaempferol-3-O-rhamnoside to similar extents. kaempferol-3-O-rhamnoside exerts similar or even slightly higher inhibitory effects on Th2 cytokines and IgE production compared with kaempferol, whereas kaempferol-3-O-rhamnoside was less effective at DPPH radical scavenging and the inhibition of ROS generation in inflammatory cells compared with kaempferol. **These results suggested that the kaempferol-3-O-rhamnoside, as well as kaempferol, may also be a promising candidate for the development of health beneficial foods or therapeutic agents that can prevent or treat allergic asthma**."	International Immunopharmacology . 25(2), 302-310 (2015)
	Afzelin attenuates asthma phenotypes by downregulation of GATA3 in a murine model of asthma. "**Afzelin is promising as a beneficial medication for the treatment of asthma through ameliorating allergic responses**"	Molecular Medicine Reports. 12, 71-76 (2015)

Table 4.2. Previously reported inhibitory effects of compounds on antigen-induced release of β-hexosaminidase from RBL-2H3 Cells

**compound**	**concentration**	**%inhibition**	**reference**
**ketotifen fumarate**	25 ug/mL (~58 uM)	20	Chemistry of Natural Compounds. 52(6), 1089–1092 (2016)
	50 ug/mL (~117 uM)	49	
	35 uM	31	International Immunopharmacology. 59, 113–119 (2018)
	15.62 ug/mL (36 uM)	60	BMC Complementary and Alternative Medicine. 19, 361(2019)

	35 uM	60	Evidence-Based Complementary and Alternative Medicine. 2020, Article ID 4307161 (2020)
**luteolin-7-O-glucoside (Cynaroside)**	25 ug/mL (~55 uM)	0.21	Chemistry of Natural Compounds. 52(6), 1089–1092 (2016)
	50 ug/mL (~111 uM)	0.59	
	10 uM	69	International Immunopharmacology. 59, 113–119 (2018)
**quercetin-3-O-rhamnoside (quercitrin)**	10uM	51	Evidence-Based Complementary and Alternative Medicine. 2020, Article ID 4307161 (2020)
	30 uM	56	
