# Supplementary material for: LC-MS/MS metabolomics-facilitated identification of the active compounds responsible for anti-allergic activity of the ethanol extract of Xenostegia tridentata
Source: PLoS One. 2022 Apr 15;17(4):e0265505. doi: 10.1371/journal.pone.0265505 (PMC9012362; doi:10.1371/journal.pone.0265505)
Supplement: S3 Table — (PDF) [file pone.0265505.s005.pdf]

S4 Table. Metabolite identification of features contributed to the discrimination between F3:EtOH/EtOAc and F5:EtOH/H2O fractions

1. **Metabolite identification of features contributed to the discrimination between F3:EtOH/EtOAc and F5:EtOH/H2O fractions in ESI positive mode.**

Feature	Predicted Formula	MS1 matching			MS2 Matching	Putative Identified Compound	MSI
		ChemiSpider	Flavonoid Mass list	*Convolvulaceae* Mass list	mzCloud		
248.13884_3.543	C11 H16 N6 O	2-(6-Hydrazino-9H-purin-9-yl)cyclohexanol					4
270.05306_5.128	C15 H10 O5	Genistein;Apigenin;Baicalein;Archin;Galangin;Aloe emodin;3',4',7-Trihydroxyisoflavone;sulfurein;6-Hydroxydaidzein;7,8,4'-trihydroxyisoflavone;2',4',7-trihydroxyisoflavone;MFCD00226431;MFCD00189455;MFCD00423934;7,8-Dihydroxy-2-(4-hydroxyphenyl)-4H-chromen-4-one;MFCD18430347;(2E)-2-(3,4-Dihydroxybenzylidene)-6-hydroxy-1-benzofuran-3(2H)-one;MFCD18324963;2-(3,4-Dihydroxybenzylidene)-6-hydroxy-1-benzofuran-3(2H)-one;MFCD18430661;6-Hydroxy-2-(6-oxo-1-oxaspiro[2.5]octa-4,7-dien-2-yl)-1-benzofuran-3(2H)-one;(2Z)-6-Hydroxy-2-[(4-hydroxyphenoxy)methylene]-1-benzofuran-3(2H)-one;1,6,7-Trihydroxy-2-methyl-9,10-anthraquinone;(2E)-6-Hydroxy-2-[(4-hydroxyphenoxy)methylene]-1-benzofuran-3(2H)-one	Sulfuretin;4,6,4'-Trihydroxyaurone;7,3',4'-Trihydroxyflavone;5,8,2'-Trihydroxyflavone;5,2',5'-Trihydroxyflavone;5,7,2'-Trihydroxyflavone;Apigenin;Baicalein;2',4'-Dihydroxy-5,6-methylenedioxy-2-phenylbenzofuran;Galangin;3'-Hydroxydaidzein;2'-Hydroxydaidzein;Demethyltexasin;8-Hydroxydaidzein;FLIAA8GS0001_a;5,7,3'-Trihydroxyisoflavone;Resokaempferol;Norwogonin;Genistein		Apigenin (90.6%);Genistein (89.6%);Galangin (88.7%);Pelargonidin (86.7%);Emodin (83.2%);5,7,8-Trihydroxyflavone (82.2%);Baicalein (82.2%);5,7-dihydroxy-3-(4-hydroxyphenyl)-4H-chromen-4-one (79.8%);Aloe-emodin (79.0%);NP-020752 (76.2%);3,5,7-trihydroxy-2-phenyl-4H-chromen-4-one (75.1%)	Apigenin	2
283.12085_3.847	C17 H17 N O3	8394939;trans-N-p-coumaroyl tyramine;N-FMOC-ETHANOLAMINE;Coumarin 334;BW A4C;2-(4-Acetylphenoxy)-N-o-tolylacetamide;MFCD00095390;N-(Phenylacetyl)phenylalanine;MFCD00739392;MFCD01071884;MFCD05874952;MFCD00412027;MFCD00522899;MFCD00745004;MFCD01181533;MFCD01181534;morphinone;MFCD01181532;N-(Phenylacetyl)phenylalanine;MFCD01340309;MFCD01216046;MFCD01195147;MFCD01213206;MFCD01195142;Heclin;MFCD01195072;MFCD01195168;MFCD01195146;KS 370G;MFCD00414766;3-(1,3-Benzodioxol-5-ylamino)-1-(4-methylphenyl)-1-propanone;MFCD03720166;MFCD18430430;MFCD02668157;MFCD02668144;MFCD18429680;MFCD00096249;MFCD184			(2E)-3-(4-Hydroxyphenyl)-N-[2-(4-hydroxyphenyl)ethyl]acrylamide (87.0%)	(2E)-3-(4-Hydroxyphenyl)-N-[2-(4-hydroxyphenyl)ethyl]acrylamide	2
286.04762_3.608	C15 H10 O6	Kaempferol;Luteolin;Scutellarein;2'-HYDROXYGENISTEIN;Orobol;Datiscetin;5,7,8,4'-tetrahydroxyisoflavone;MFCD16621432;5,6,7-Trihydroxy-3-(4-hydroxyphenyl)-4H-chromen-4-one;7,8,3',4'-Tetrahydroxyisoflavone;6,7,3',4'-Tetrahydroxyisoflavone;1,3,8-Trihydroxy-2-methoxy-9,10-anthraquinone;1,2,3,8-Tetrahydroxy-7-methyl-9,10-anthraquinone	Maritimetin;2'-Hydroxygenistein;Orobol;6-Hydroxygenistein;7,8,2',4'-Tetrahydroxyisoflavone;Baptigenin;6-Hydroxygalangin;Kaempferol;Datiscetin;3,7,8,4'-Tetrahydroxyflavone;3,3',4',7-Tetrahydroxyflavone;8-Hydroxygalangin;Aureusidin;Norartocarpetin;4',5,7,8-Tetrahydroxyflavone;FL3FF8GS0006_a;Scutellarein;FL3FE8GS0001_a;5,7,2',3'-Tetrahydroxyflavone;5,7,2',6'-Tetrahydroxyflavone;5,7,2',5'-Tetrahydroxyflavone;7,3',4',5'-Tetrahydroxyflavone;FL1CQUGS0001_a;Helmon;Luteolin;FLNAACGS0001_a	kaempferol;luteolin	Kaempferol (87.3%);Fisetin (82.6%);4-(methylthio)-6-[4-(trifluoromethyl)phenyl]-1,3,5-triazin-2-amine (79.8%);Luteolin (78.6%);NP-013249 (77.8%);Cyanidin (77.7%);2,6-di(2-thienylmethylidene)cyclohexan-1-one (76.9%)	Flavonoids	3
286.04762_3.212	C15 H10 O6	Kaempferol;Luteolin;Scutellarein;2'-HYDROXYGENISTEIN;Orobol;Datiscetin;5,7,8,4'-tetrahydroxyisoflavone;MFCD16621432;5,6,7-Trihydroxy-3-(4-hydroxyphenyl)-4H-chromen-4-one;7,8,3',4'-Tetrahydroxyisoflavone;6,7,3',4'-Tetrahydroxyisoflavone;1,3,8-Trihydroxy-2-methoxy-9,10-anthraquinone;1,2,3,8-Tetrahydroxy-7-methyl-9,10-anthraquinone	Maritimetin;2'-Hydroxygenistein;Orobol;6-Hydroxygenistein;7,8,2',4'-Tetrahydroxyisoflavone;Baptigenin;6-Hydroxygalangin;Kaempferol;Datiscetin;3,7,8,4'-Tetrahydroxyflavone;3,3',4',7-Tetrahydroxyflavone;8-Hydroxygalangin;Aureusidin;Norartocarpetin;4',5,7,8-Tetrahydroxyflavone;FL3FF8GS0006_a;Scutellarein;FL3FE8GS0001_a;5,7,2',3'-Tetrahydroxyflavone;5,7,2',6'-Tetrahydroxyflavone;5,7,2',5'-Tetrahydroxyflavone;7,3',4',5'-Tetrahydroxyflavone;FL1CQUGS0001_a;Helmon;Luteolin;FLNAACGS0001_a	kaempferol;luteolin	Kaempferol (79.5%);4-(methylthio)-6-[4-(trifluoromethyl)phenyl]-1,3,5-triazin-2-amine (79.5%);Cyanidin (77.5%);Fisetin (77.4%);2,6-di(2-thienylmethylidene)cyclohexan-1-one (76.6%);Luteolin (75.3%)	Flavonoids	3
292.20366_6.335	C18 H28 O3	(9S,13S)-12-Oxophytodienoic acid;p-Undecyloxybenzoic Acid;(2,4-Di-tert-pentylphenoxy)acetic acid;Methyl 4-(decyloxy)benzoate;9,10-EOT;colnelenic acid;MFCD01095820;Decyl 4-methoxybenzoate;MFCD00229195;MFCD00026534;MFCD00021059;Decyl 3-methoxybenzoate;MFCD11502129;Decyl 2-methoxybenzoate;MFCD09868700					4
302.0424_3.377	C15 H10 O7	Quercetin;Morin;Tricetin;Herbacetin;3-(3,4-Dihydroxyphenyl)-5,6,7-trihydroxy-4H-chromen-4-one;3-(3,4-Dihydroxyphenyl)-5,7,8-trihydroxy-4H-chromen-4-one;1,3,5,8-Tetrahydroxy-2-methoxy-9,10-anthraquinone	Bracteatin;5,7,8,2',4'-Pentahydroxyisoflavone;6-Hydroxykaempferol;Morin;3,5,7,2',5'-Pentahydroxyflavone;3,7,8,3',4'-Pentahydroxyflavone;Rhynchosin;Viscidulin I;Robinetin;Herbacetin;Quercetin;Isoetin;Tricetin;Hypolaetin;6-Hydroxyluteolin;FL5F1LGL0001_a	quercetin	Quercetin (88.0%);Morin (85.6%);Robinetin (84.1%);2-(2,6-dihydroxyphenyl)-3,5,7-trihydroxy-4H-chromen-4-one (81.4%);2-(2,4-dihydroxyphenyl)-3,5,7-trihydroxy-4H-chromen-4-one (81.4%);5,7-dihydroxy-2-(2,3,4-trihydroxyphenyl)-4H-chromen-4-	Flavonoids	3
302.04244_2.959	C15 H10 O7	Quercetin;Morin;Tricetin;Herbacetin;3-(3,4-Dihydroxyphenyl)-5,6,7-trihydroxy-4H-chromen-4-one;3-(3,4-Dihydroxyphenyl)-5,7,8-trihydroxy-4H-chromen-4-one;1,3,5,8-Tetrahydroxy-2-methoxy-9,10-anthraquinone	Bracteatin;5,7,8,2',4'-Pentahydroxyisoflavone;6-Hydroxykaempferol;Morin;3,5,7,2',5'-Pentahydroxyflavone;3,7,8,3',4'-Pentahydroxyflavone;Rhynchosin;Viscidulin I;Robinetin;Herbacetin;Quercetin;Isoetin;Tricetin;Hypolaetin;6-Hydroxyluteolin;FL5F1LGL0001_a	quercetin	Quercetin (77.1%); 2-(2,6-dihydroxyphenyl)-3,5,7-trihydroxy-4H-chromen-4-one (76.2%)	Flavonoids	3
302.04245_3.253	C15 H10 O7	Quercetin;Morin;Tricetin;Herbacetin;3-(3,4-Dihydroxyphenyl)-5,6,7-trihydroxy-4H-chromen-4-one;3-(3,4-Dihydroxyphenyl)-5,7,8-trihydroxy-4H-chromen-4-one;1,3,5,8-Tetrahydroxy-2-methoxy-9,10-anthraquinone	Bracteatin;5,7,8,2',4'-Pentahydroxyisoflavone;6-Hydroxykaempferol;Morin;3,5,7,2',5'-Pentahydroxyflavone;3,7,8,3',4'-Pentahydroxyflavone;Rhynchosin;Viscidulin I;Robinetin;Herbacetin;Quercetin;Isoetin;Tricetin;Hypolaetin;6-Hydroxyluteolin;FL5F1LGL0001_a	quercetin	Quercetin (76.3%); robinetin (76.3%)	Flavonoids	3
311.18852_6.41	C14 H26 N5 O P	Adiphenine;Dienogest;2372;MFCD01847300;MFCD01180578;MFCD01180548;MFCD02705168;MFCD01180542;MFCD01180543;2-((4-CYCLOHEXYLANILINO)METHYL)-5-METHOXYPHENOL;MFCD01180546;1-{4-[2-(Dimethylamino)ethoxy]phenyl}-2-phenyl-1-butanone;MFCD01180547;MFCD02964568;MFCD00027730;MFCD03093974;MFCD02342238;MFCD00027727;MFCD02079522;3-(4-SEC-BUTYLANILINO)-1-(4-METHOXYPHENYL)-1-PROPANONE;MFCD03093973;MFCD02666651;MFCD01757476;MFCD10569647;9-Dodecyn-1-yl 4-cyanobenzoate;MFCD00225384;3-(Dimethylamino)-1-(4-methylphenyl)-1-phenylpropyl acetate;4-[3-(Dimethylamino)-1-(4-methylphenyl)propyl]phenyl acetate;Benzyl N-benzyl-3-methyl-L-valinate					4
312.19101_6.293	C13 H24 N6 O3	{4-[(2,6-Diisopropylphenoxy)methyl]phenyl}boronic acid;{2-[(2,6-Diisopropylphenoxy)methyl]phenyl}boronic acid					4

Feature	Predicted Formula	MS1 matching			MS2 Matching	Putative Identified Compound	MSI
		ChemiSpider	Flavonoid Mass list	*Convolvulaceae* Mass list	mzCloud		
324.21996_5.206	C21 H28 N2 O	OS9545500;MFCD00218394;MFCD01071200;diampromide;MFCD00085867;MFCD00027738;MFCD18428730;N-Benzyl-N-[6-(2-cyanoethyl)-1-cyclohexen-1-yl]pentanamide;N-Benzyl-N-[6-(2-cyanoethyl)-1-cyclohexen-1-yl]-3-methylbutanamide;1,3-Bis[2-(2-methyl-2-propanyl)phenyl]urea					4
432.10586_3.357	C21 H20 O10	5-Hydroxy-3-(4-hydroxyphenyl)-4-oxo-4H-chromen-7-yl hexopyranoside;Apigetrin;beta-D-Genistin;vitexin;Afzelin;Isovitexin;MFCD01725365;Baicalein 7-b-D-glucopyranoside;Genistin;MFCD00275821;MFCD00228892;Sophoricoside;3-(4-Hydroxyphenyl)-4-oxo-3,4-dihydro-2H-chromen-7-yl beta-D-glucopyranosiduronic acid;(1S)-1,5-Anhydro-1-[5,7-dihydroxy-3-(4-hydroxyphenyl)-4-oxo-4H-chromen-6-yl]-D-glucitol;(2Z)-2-(3,4-Dihydroxybenzylidene)-3-oxo-2,3-dihydro-1-benzofuran-6-yl beta-D-glucopyranoside;3-(beta-D-Glucopyranosyloxy)-7-hydroxy-2-(4-hydroxyphenyl)-5-chromeniumolate;5-[5-(beta-D-Glucopyranosyloxy)-7-hydroxy-2-chromeniumyl]-2-hydroxyphenolate;4-{[(Z)-(6-Hydroxy-3-oxo-1-benzofuran-2(3H)-ylidene)methyl]oxy}phenyl beta-D-glucopyranoside;(2Z)-2-(3,4-Dihydroxybenzylidene)-3-oxo-2,3-dihydro-1-benzofuran-6-yl D-glucopyranoside;(2Z)-2-(3,4-Dihydroxybenzylidene)-3-oxo-2,3-dihydro-1-benzofuran-6-yl hexopyranoside	Baicalein 6-glucoside;7,3',4',5'-Tetrahydroxyflavone 7-rhamnoside;6-C-Fucopyranosylluteolin;Scutellarein 7-rhamnoside;6-C-Chinovopyranosylluteolin;Apigenin 5-galactoside;Apigenin 7-galactoside;Apigenin 5-glucoside;Apigenin 7-O-glucoside;FL3FAACS0092_a;Neovitexin;6-C-beta-D-Galactosylapigenin;8-C-beta-D-Galactopyranosylapigenin;Vitexin;5,7,2'-Trihydroxy 7-glucoside;7,3',4'-Trihydroxyflavone 7-galactoside;Apigenin 4'-glucoside;Kaempferol 7-rhamnoside;Luteolin 3'-methyl ether 7-xyloside;5,7,8-Trihydroxyflavone 7-galactoside;Kaempferol 5-rhamnoside;Afzelin;Pueraria glycoside 1;6,7,4'-Trihydroxyisoflavone 4'-O-glucoside;Isogenistein 7-O-glucoside;Genistein 8-C-glucoside;Genistin;Genistein 4'-O-glucoside;Genistein 5-O-glucoside;Galangin 7-glucoside;5,7,8-Trihydroxyflavone 5-glucoside;Galanginin;Resokaempferol 7-glucoside;Resokaempferol 3-glucoside;8-C-Glucosyl-5-deoxykaempferol;3'-Hydroxy-3,5,8,4',5'-pentamethoxy-6,7-methylenedioxyflavone;Kaempferide 3-alpha-L-arabinopyranoside;Kaempferol 4'-rhamnoside;FL3FADCS0022_a;Luteolin 3'-rhamnoside;Luteolin 7-rhamnoside;8-C-Rhamnopyranosylluteolin;Resokaempferol 4'-glucoside;7,3',4'-Trihydroxyflavone 7-glucoside;Isovitexin;Sulfurein;Aureusidin 6-rhamnoside		Genistin (94.6%);Apigetrin (94.6%);1,5-Anhydro-1-[5,7-dihydroxy-3-(4-hydroxyphenyl)-4-oxo-4H-chromen-8-yl]hexitol (80.5%);5-hydroxy-3-(4-hydroxyphenyl)-7-{[(2S,3R,4S,5S,6R)-3,4,5-trihydroxy-6-(hydroxymethyl)oxan-2-yl]oxy}-4H-chromen-4-one (79.7%)	Apigetrin (Apigenin-7-O-glucoside)	2
**432.10612_3.604**	C21 H20 O10	5-Hydroxy-3-(4-hydroxyphenyl)-4-oxo-4H-chromen-7-yl hexopyranoside;Apigetrin;beta-D-Genistin;vitexin;Afzelin;Isovitexin;MFCD01725365;Baicalein 7-b-D-glucopyranoside;Genistin;MFCD00275821;MFCD00228892;Sophoricoside;3-(4-Hydroxyphenyl)-4-oxo-3,4-dihydro-2H-chromen-7-yl beta-D-glucopyranosiduronic acid;(1S)-1,5-Anhydro-1-[5,7-dihydroxy-3-(4-hydroxyphenyl)-4-oxo-4H-chromen-6-yl]-D-glucitol;(2Z)-2-(3,4-Dihydroxybenzylidene)-3-oxo-2,3-dihydro-1-benzofuran-6-yl beta-D-glucopyranoside;3-(beta-D-Glucopyranosyloxy)-7-hydroxy-2-(4-hydroxyphenyl)-5-chromeniumolate;5-[5-(beta-D-Glucopyranosyloxy)-7-hydroxy-2-chromeniumyl]-2-hydroxyphenolate;4-{[(Z)-(6-Hydroxy-3-oxo-1-benzofuran-2(3H)-ylidene)methyl]oxy}phenyl beta-D-glucopyranoside;(2Z)-2-(3,4-Dihydroxybenzylidene)-3-oxo-2,3-dihydro-1-benzofuran-6-yl D-glucopyranoside;(2Z)-2-(3,4-Dihydroxybenzylidene)-3-oxo-2,3-dihydro-1-benzofuran-6-yl hexopyranoside	Baicalein 6-glucoside;7,3',4',5'-Tetrahydroxyflavone 7-rhamnoside;6-C-Fucopyranosylluteolin;Scutellarein 7-rhamnoside;6-C-Chinovopyranosylluteolin;Apigenin 5-galactoside;Apigenin 7-galactoside;Apigenin 5-glucoside;Apigenin 7-O-glucoside;FL3FAACS0092_a;Neovitexin;6-C-beta-D-Galactosylapigenin;8-C-beta-D-Galactopyranosylapigenin;Vitexin;5,7,2'-Trihydroxy 7-glucoside;7,3',4'-Trihydroxyflavone 7-galactoside;Apigenin 4'-glucoside;Kaempferol 7-rhamnoside;Luteolin 3'-methyl ether 7-xyloside;5,7,8-Trihydroxyflavone 7-galactoside;Kaempferol 5-rhamnoside;Afzelin;Pueraria glycoside 1;6,7,4'-Trihydroxyisoflavone 4'-O-glucoside;Isogenistein 7-O-glucoside;Genistein 8-C-glucoside;Genistin;Genistein 4'-O-glucoside;Genistein 5-O-glucoside;Galangin 7-glucoside;5,7,8-Trihydroxyflavone 5-glucoside;Galanginin;Resokaempferol 7-glucoside;Resokaempferol 3-glucoside;8-C-Glucosyl-5-deoxykaempferol;3'-Hydroxy-3,5,8,4',5'-pentamethoxy-6,7-methylenedioxyflavone;Kaempferide 3-alpha-L-arabinopyranoside;Kaempferol 4'-rhamnoside;FL3FADCS0022_a;Luteolin 3'-rhamnoside;Luteolin 7-rhamnoside;8-C-Rhamnopyranosylluteolin;Resokaempferol 4'-glucoside;7,3',4'-Trihydroxyflavone 7-glucoside;Isovitexin;Sulfurein;Aureusidin 6-rhamnoside		Afzelin (86.8%)	Afzelin (Kaempferol-3-O-rhamnoside)	2
448.1005_3.147	C21 H20 O11	Quercitrin;Astragalin;Cynaroside;Orientin;Isoorientin;kaempferol 7-O-beta-D-glucopyranoside;Trifolin;Naringenin 7-O-beta-D-glucuronide;4-(5,7-Dihydroxy-4-oxo-3,4-dihydro-2H-chromen-2-yl)phenyl beta-D-glucopyranosiduronic acid;MFCD13186795;aureusidin 6-O-beta-glucoside;7-Hydroxy-2-(4-hydroxyphenyl)-4-oxo-3,4-dihydro-2H-chromen-5-yl beta-D-glucopyranosiduronic acid;Kaempferol 3-O-D-galactoside;cyanidin 3-O-beta-D-glucoside betaine;cyanidin 3-O-beta-D-galactoside betaine;2-(3,4-Dihydroxyphenyl)-3-(hexopyranosyloxy)-7-hydroxy-5-chromeniumolate;5-[3-(D-Glucopyranosyloxy)-5,7-dihydroxy-2-chromeniumyl]-2-hydroxyphenolate	Kaempferol 3-alpha-D-galactoside;8-C-beta-D-Glucopyranosylkaempferol;6-C-Glucopyranosylkaempferol;Datiscanin;Fisetin 4'-glucoside;Fisetin 7-glucoside;Fisetin 3-glucoside;8-C-Glucosylfisetin;Herbacetin 8-rhamnoside;5,7,2',6'-Tetrahydroxyflavone 2'-O-glucoside;evolvuside B;6-Hydroxyluteolin 5-rhamnoside;6-Hydroxyluteolin 7-rhamnoside;6-Hydroxyluteolin 6-rhamnoside;6-C-Galactosylisoscutellarein;Isoscutellarein 7-glucoside;Hypolaetin 8-rhamnoside;8-C-Galactosylluteolin;Herbacetin 7-rhamnoside;Isorhamnetin 3-xyloside;Distichin;Isorhamnetin 3-alpha-L-arabinofuranoside;Quercetin 7-rhamnoside;Quercitrin;8-C-Methylquercetin 3-xyloside;Luteolin 4'-glucoside;Luteolin 3'-glucoside;Luteolin 7-galactoside;Luteolin-7-glucoside;Galuteolin;Luteolin 5-galactoside;Isoorientin;6-C-Galactosylluteolin;5,7,3',4'-Tetrahydroxy-4-phenylcoumarin 5-O-glucoside;Orobol 7-O-glucoside;6-C-Glucosylorobol;8-C-Glucosylorobol;Quercetin 3-methyl ether 3'-xyloside;Rhamnetin 3-alpha-L-arabinopyranoside;Rhamnetin 3-alpha-L-arabinofuranoside;Kaempferol 4'-glucoside;Populnin;Kaempferol 7-galactoside;Kaempferol 7-alloside;Kaempferol 5-glucoside;Asiaticalin;Orientin;Kaempferol 3-alpha-D-glucoside;Trifolin;Scutellarein 6-glucoside;Dihydronorwogonin 7-O-glucuronide;Dihydrobaicalein 7-O-glucuronide;Carthamone;Aureusin;Cernuoside;Astragalin;Maritimetin 7-glucoside; Scutellarein 7-glucoside; Maritimein	lutoelin-7-O-D-glucoside	Cynaroside (92.3%);Kuromanin (91.3%);Trifolin (85.9%);5,7-dihydroxy-2-(3-hydroxy-4-{[(2S,3R,4S,5S,6R)-3,4,5-trihydroxy-6-(hydroxymethyl)oxan-2-yl]oxy}phenyl)-4H-chromen-4-one (83.7%);2-(3,4-dihydroxyphenyl)-5,7-dihydroxy-3-{[(2S,3R,4R,5R,6S)-3,4,5-trihydroxy-6-methyloxan-2-yl]oxy}-4H-chromen-4-one (83.5%);Quercitrin (82.8%);Kaempferol-7-O-glucoside (82.2%);4-(3,4-dihydroxyphenyl)-7-hydroxy-5-{[(2S,3R,4S,5S,6R)-3,4,5-trihydroxy-6-(hydroxymethyl)oxan-2-yl]oxy}-2H-chromen-2-one (78.1%)	Flavonoid glycosides	3

Feature	Predicted Formula	MS1 matching			MS2 Matching	Putative Identified Compound	MSI
		ChemiSpider	Flavonoid Mass list	*Convolvulaceae* Mass list	mzCloud		
**448.10082_3.034**	C21 H20 O11	Quercitrin;Astragalin;Cynaroside;Orientin;Isoorientin;kaempferol 7-O-beta-D-glucopyranoside;Trifolin;Naringenin 7-O-beta-D-glucuronide;4-(5,7-Dihydroxy-4-oxo-3,4-dihydro-2H-chromen-2-yl)phenyl beta-D-glucopyranosiduronic acid;MFCD13186795;aureusidin 6-O-beta-glucoside;7-Hydroxy-2-(4-hydroxyphenyl)-4-oxo-3,4-dihydro-2H-chromen-5-yl beta-D-glucopyranosiduronic acid;Kaempferol 3-O-D-galactoside;cyanidin 3-O-beta-D-glucoside betaine;cyanidin 3-O-beta-D-galactoside betaine;2-(3,4-Dihydroxyphenyl)-3-(hexopyranosyloxy)-7-hydroxy-5-chromeniumolate;5-[3-(D-Glucopyranosyloxy)-5,7-dihydroxy-2-chromeniumyl]-2-hydroxyphenolate	Kaempferol 3-alpha-D-galactoside;8-C-beta-D-Glucopyranosylkaempferol;6-C-Glucopyranosylkaempferol;Datiscanin;Fisetin 4'-glucoside;Fisetin 7-glucoside;Fisetin 3-glucoside;8-C-Glucosylfisetin;Herbacetin 8-rhamnoside;5,7,2',6'-Tetrahydroxyflavone 2'-O-glucoside;evolvuside B;6-Hydroxyluteolin 5-rhamnoside;6-Hydroxyluteolin 7-rhamnoside;6-Hydroxyluteolin 6-rhamnoside;6-C-Galactosylisoscutellarein;Isoscutellarein 7-glucoside;Hypolaetin 8-rhamnoside;8-C-Galactosylluteolin;Herbacetin 7-rhamnoside;Isorhamnetin 3-xyloside;Distichin;Isorhamnetin 3-alpha-L-arabinofuranoside;Quercetin 7-rhamnoside;Quercitrin;8-C-Methylquercetin 3-xyloside;Luteolin 4'-glucoside;Luteolin 3'-glucoside;Luteolin 7-galactoside;Luteolin-7-glucoside;Galuteolin;Luteolin 5-galactoside;Isoorientin;6-C-Galactosylluteolin;5,7,3',4'-Tetrahydroxy-4-phenylcoumarin 5-O-glucoside;Orobol 7-O-glucoside;6-C-Glucosylorobol;8-C-Glucosylorobol;Quercetin 3-methyl ether 3'-xyloside;Rhamnetin 3-alpha-L-arabinopyranoside;Rhamnetin 3-alpha-L-arabinofuranoside;Kaempferol 4'-glucoside;Populnin;Kaempferol 7-galactoside;Kaempferol 7-alloside;Kaempferol 5-glucoside;Asiaticalin;Orientin;Kaempferol 3-alpha-D-glucoside;Trifolin;Scutellarein 6-glucoside;Dihydronorwogonin 7-O-glucuronide;Dihydrobaicalein 7-O-glucuronide;Carthamone;Aureusin;Cernuoside;Astragalin;Maritimetin 7-glucoside; Scutellarein 7-glucoside; Maritimein	lutoelin-7-O-D-glucoside	Kuromanin (96.8%);Cynaroside (91.3%);Kaempferol-7-O-glucoside (85.5%);Trifolin (83.6%);4-(3,4-dihydroxyphenyl)-7-hydroxy-5-{[(2S,3R,4S,5S,6R)-3,4,5-trihydroxy-6-(hydroxymethyl)oxan-2-yl]oxy}-2H-chromen-2-one (81.2%);(1ξ)-1,5-Anhydro-1-[2-(3,4-dihydroxyphenyl)-5,7-dihydroxy-4-oxo-4H-chromen-8-yl]-D-galactitol (78.8%);5-(5,7-Dihydroxy-3-methoxy-4-oxo-4H-chromen-2-yl)-2-hydroxyphenyl beta-D-xylopyranoside (77.3%)	Cynaroside (luteolin-7-O-glucoside)	2
448.10088_3.492	C21 H20 O11	Quercitrin;Astragalin;Cynaroside;Orientin;Isoorientin;kaempferol 7-O-beta-D-glucopyranoside;Trifolin;Naringenin 7-O-beta-D-glucuronide;4-(5,7-Dihydroxy-4-oxo-3,4-dihydro-2H-chromen-2-yl)phenyl beta-D-glucopyranosiduronic acid;MFCD13186795;aureusidin 6-O-beta-glucoside;7-Hydroxy-2-(4-hydroxyphenyl)-4-oxo-3,4-dihydro-2H-chromen-5-yl beta-D-glucopyranosiduronic acid;Kaempferol 3-O-D-galactoside;cyanidin 3-O-beta-D-glucoside betaine;cyanidin 3-O-beta-D-galactoside betaine;2-(3,4-Dihydroxyphenyl)-3-(hexopyranosyloxy)-7-hydroxy-5-chromeniumolate;5-[3-(D-Glucopyranosyloxy)-5,7-dihydroxy-2-chromeniumyl]-2-hydroxyphenolate	Kaempferol 3-alpha-D-galactoside;8-C-beta-D-Glucopyranosylkaempferol;6-C-Glucopyranosylkaempferol;Datiscanin;Fisetin 4'-glucoside;Fisetin 7-glucoside;Fisetin 3-glucoside;8-C-Glucosylfisetin;Herbacetin 8-rhamnoside;5,7,2',6'-Tetrahydroxyflavone 2'-O-glucoside;evolvuside B;6-Hydroxyluteolin 5-rhamnoside;6-Hydroxyluteolin 7-rhamnoside;6-Hydroxyluteolin 6-rhamnoside;6-C-Galactosylisoscutellarein;Isoscutellarein 7-glucoside;Hypolaetin 8-rhamnoside;8-C-Galactosylluteolin;Herbacetin 7-rhamnoside;Isorhamnetin 3-xyloside;Distichin;Isorhamnetin 3-alpha-L-arabinofuranoside;Quercetin 7-rhamnoside;Quercitrin;8-C-Methylquercetin 3-xyloside;Luteolin 4'-glucoside;Luteolin 3'-glucoside;Luteolin 7-galactoside;Luteolin-7-glucoside;Galuteolin;Luteolin 5-galactoside;Isoorientin;6-C-Galactosylluteolin;5,7,3',4'-Tetrahydroxy-4-phenylcoumarin 5-O-glucoside;Orobol 7-O-glucoside;6-C-Glucosylorobol;8-C-Glucosylorobol;Quercetin 3-methyl ether 3'-xyloside;Rhamnetin 3-alpha-L-arabinopyranoside;Rhamnetin 3-alpha-L-arabinofuranoside;Kaempferol 4'-glucoside;Populnin;Kaempferol 7-galactoside;Kaempferol 7-alloside;Kaempferol 5-glucoside;Asiaticalin;Orientin;Kaempferol 3-alpha-D-glucoside;Trifolin;Scutellarein 6-glucoside;Dihydronorwogonin 7-O-glucuronide;Dihydrobaicalein 7-O-glucuronide;Carthamone;Aureusin;Cernuoside;Astragalin;Maritimetin 7-glucoside; Scutellarein 7-glucoside; Maritimein	lutoelin-7-O-D-glucoside	Cynaroside (87.7%);Kuromanin (86.6%);5,7-dihydroxy-2-(3-hydroxy-4-{[(2S,3R,4S,5S,6R)-3,4,5-trihydroxy-6-(hydroxymethyl)oxan-2-yl]oxy}phenyl)-4H-chromen-4-one (83.4%);Quercitrin (82.9%);2-(3,4-dihydroxyphenyl)-5,7-dihydroxy-3-{[(2S,3R,4R,5R,6S)-3,4,5-trihydroxy-6-methyloxan-2-yl]oxy}-4H-chromen-4-one (81.3%);Kaempferol-7-O-glucoside (79.9%);Trifolin (78.3%)	Flavonoid glycosides	3
**448.10108_3.251**	C21 H20 O11	Quercitrin;Astragalin;Cynaroside;Orientin;Isoorientin;kaempferol 7-O-beta-D-glucopyranoside;Trifolin;Naringenin 7-O-beta-D-glucuronide;4-(5,7-Dihydroxy-4-oxo-3,4-dihydro-2H-chromen-2-yl)phenyl beta-D-glucopyranosiduronic acid;MFCD13186795;aureusidin 6-O-beta-glucoside;7-Hydroxy-2-(4-hydroxyphenyl)-4-oxo-3,4-dihydro-2H-chromen-5-yl beta-D-glucopyranosiduronic acid;Kaempferol 3-O-D-galactoside;cyanidin 3-O-beta-D-glucoside betaine;cyanidin 3-O-beta-D-galactoside betaine;2-(3,4-Dihydroxyphenyl)-3-(hexopyranosyloxy)-7-hydroxy-5-chromeniumolate;5-[3-(D-Glucopyranosyloxy)-5,7-dihydroxy-2-chromeniumyl]-2-hydroxyphenolate	Kaempferol 3-alpha-D-galactoside;8-C-beta-D-Glucopyranosylkaempferol;6-C-Glucopyranosylkaempferol;Datiscanin;Fisetin 4'-glucoside;Fisetin 7-glucoside;Fisetin 3-glucoside;8-C-Glucosylfisetin;Herbacetin 8-rhamnoside;5,7,2',6'-Tetrahydroxyflavone 2'-O-glucoside;evolvuside B;6-Hydroxyluteolin 5-rhamnoside;6-Hydroxyluteolin 7-rhamnoside;6-Hydroxyluteolin 6-rhamnoside;6-C-Galactosylisoscutellarein;Isoscutellarein 7-glucoside;Hypolaetin 8-rhamnoside;8-C-Galactosylluteolin;Herbacetin 7-rhamnoside;Isorhamnetin 3-xyloside;Distichin;Isorhamnetin 3-alpha-L-arabinofuranoside;Quercetin 7-rhamnoside;Quercitrin;8-C-Methylquercetin 3-xyloside;Luteolin 4'-glucoside;Luteolin 3'-glucoside;Luteolin 7-galactoside;Luteolin-7-glucoside;Galuteolin;Luteolin 5-galactoside;Isoorientin;6-C-Galactosylluteolin;5,7,3',4'-Tetrahydroxy-4-phenylcoumarin 5-O-glucoside;Orobol 7-O-glucoside;6-C-Glucosylorobol;8-C-Glucosylorobol;Quercetin 3-methyl ether 3'-xyloside;Rhamnetin 3-alpha-L-arabinopyranoside;Rhamnetin 3-alpha-L-arabinofuranoside;Kaempferol 4'-glucoside;Populnin;Kaempferol 7-galactoside;Kaempferol 7-alloside;Kaempferol 5-glucoside;Asiaticalin;Orientin;Kaempferol 3-alpha-D-glucoside;Trifolin;Scutellarein 6-glucoside;Dihydronorwogonin 7-O-glucuronide;Dihydrobaicalein 7-O-glucuronide;Carthamone;Aureusin;Cernuoside;Astragalin;Maritimetin 7-glucoside; Scutellarein 7-glucoside; Maritimein	lutoelin-7-O-D-glucoside	Astragalin (82.4%) ; Quercitrin (83.5%)	Quercitrin (Quercetin-3-O-rhamnoside)	2
458.11871_3.523	C10 H26 N4 O14 S	MFCD00225625;MFCD09266099;MFCD09266101;MFCD07787740;MFCD05151986;MFCD09266012					4
458.21517_7.888	C23 H30 N4 O6	4-{3,4-Bis[(trimethylsilyl)oxy]phenyl}-2,2,7,7-tetramethyl-3,6-dioxa-2,7-disilaoctane;MFCD00024933					4

Feature	Predicted Formula	MS1 matching			MS2 Matching	Putative Identified Compound	MSI
		ChemiSpider	Flavonoid Mass list	*Convolvulaceae* Mass list	mzCloud		
462.11648_3.521	C22 H22 O11	tectoridin;tectoridin;MFCD00546751;MFCD24849323;MFCD03090837;5,7-Dihydroxy-2-(4-methoxyphenyl)-4-oxo-4H-chromen-3-yl beta-D-glucopyranoside	5,7,2'-Trihydroxy-6-methoxyflavone 7-O-glucoside;Rhamnetin 3-rhamnoside;Isorhamnetin 3-rhamnoside;5,3',4'-Trihydroxy-7-methoxy-4-phenylcoumarin 5-O-glucoside;Isotectorigenin 7-O-glucoside;Tectoridin;Pratensein 7-O-glucoside;3'-O-Methylorobol 7-O-glucoside;Dalpanitin;6-Methoxykaempferol 4'-rhamnoside;6-Methoxykaempferol 3-rhamnoside;Vogelin;3-O-Methylquercetin 7-O-rhamnopyranoside;Isokaempferide 7-glucoside;Ombuin 3-arabinofuranoside;FL5FCCDS0001_a;Luteolin 3'-methyl ether 7-glucoside;Luteolin 3'-methyl ether 4'-glucoside;8-C-beta-D-Glucopyranosyldiosmetin;6-C-beta-D-Glucopyranosyldiosmetin;Luteolin 4'-methyl ether 7-glucoside;Diosmetin 3'-glucoside;Tricin 7-xyloside;Setaricin;Tricin 4'-apioside;8-D-Glucopyranosyl-7,3',4'-trihydroxy-5-methoxyflavone;Luteolin 5-methyl ether 7-glucoside;Luteorin 5-methyl ether 3'-glucoside;Leucanthoside;Isoswertiajaponin;Luteolin 7-methyl ether 5-glucoside;Mumenin;Isorhamnetin 7-rhamnoside;5,3',4'-Trihydroxy-7-methoxy-4-phenylcoumarin 5-O-galactoside;Tamarixetin 3-rhamnoside;6-C-Methylkaempferol 3-glucoside;(-) -Isoscoparin;Isoscoparin;Isoscutellarein 4'-methyl ether 8-glucoside;6-Methoxyluteolin 7-rhamnoside;Hispidulin 4'-glucoside;Scutellarein 4'-methyl ether 7-glucoside;8-C-beta-D-Glucopyranosylchrysoeriol;Scutellarein 7-methyl ether 6-glucoside;Hispidulin 7-glucoside;Kaempferol 7-methyl ether 4'-glucoside;Rhamnocitrin 5-glucoside;Geraldol 4'-glucoside;5,2',6'-Trihydroxy-7-methoxyflavone 2'-O-glucoside;Leptosin;Scutellarein 7-methyl ether 6-galactoside;Episcoparin;Rhamnocitrin 3-glucoside;Kaempferol 5-methyl ether 3-galactoside;Azaleatin 3-rhamnoside;6-beta-D-Glucopyranosyl-3,4',5-trihydroxy-7-methoxyflavone;Rhamnocitrin 3-galactoside;Rhamnocitrin 3-alloside;Kaempferide 3-galactoside;Kaempferide 3-glucoside;Luteolin 3'-methyl ether 5-glucoside;8-C-Methylkaempferol 7-glucoside	diosmetin-7-O-D-glucoside	5,7-dihydroxy-6-methoxy-2-(4-{[(2S,3R,4S,5S,6R)-3,4,5-trihydroxy-6-(hydroxymethyl)oxan-2-yl]oxy}phenyl)-4H-chromen-4-one (81.6%);5-hydroxy-2-(4-hydroxyphenyl)-6-methoxy-7-{[(2S,3R,4S,5S,6R)-3,4,5-trihydroxy-6-(hydroxymethyl)oxan-2-yl]oxy}-4H-chromen-4-one (81.5%)	Methoxy flavonoid glycosides	3
482.12103_3.77	C25 H22 O10	4105;Silymarin;Silidianin;(3S)-3,5,7-Trihydroxy-2-[(2S,3S)-3-(4-hydroxy-3-methoxyphenyl)-2-(hydroxymethyl)-2,3-dihydro-1,4-benzodioxin-6-yl]-2,3-dihydro-4H-chromen-4-one;ISOSILYBIN B;Silibinin	Silychristin;Isosilybin;Silydianin;Isosilychristin;Silybin			Silymarin (flavonolignans)	3
482.12115_3.515	C25 H22 O10	4105;Silymarin;Silidianin;(3S)-3,5,7-Trihydroxy-2-[(2S,3S)-3-(4-hydroxy-3-methoxyphenyl)-2-(hydroxymethyl)-2,3-dihydro-1,4-benzodioxin-6-yl]-2,3-dihydro-4H-chromen-4-one;ISOSILYBIN B;2,2,4,6,8-Pentamethyl-4,6,8-triphenyl-1,3,5,7,2,4,6,8-tetroxatetrasilocane;Silibinin;MFCD03933352;MFCD05150758;MFCD05150775	Silychristin;Isosilybin;Silydianin;Isosilychristin;Silybin			Silymarin (flavonolignans)	3
498.11685_4.286	C26 H18 N4 O7	MFCD00767685;MFCD02726012					4
512.13159_3.981	C22 H20 N6 O9	MFCD03212222;MFCD03284107;MFCD03462902;MFCD03212701;MFCD03462897;MFCD04441745;MFCD03091035;MFCD01458020					4
512.13175_4.317	C23 H16 N10 O5	MFCD03212222;MFCD03284107;MFCD03462902;MFCD03212701;MFCD03462897;MFCD04441745;MFCD03417501;MFCD03091035;MFCD01458020					4
516.12723_3.168	C25 H24 O12	Cynarine;MFCD22375481;3,4-Dicaffeoylquinic acid;Cynarin;3,5-O-dicaffeoylquinic acid	2",6"-Di-O-Acetyl isovitexin;Apigenin 7- (3",4"-diacetylglucoside);Kaempferol 3- (3",4"-diacetylrhamnoside);Formononetin 7-O- (6"-malonylglucoside);Kaempferol 3- (2",4"-diacetylrhamnoside);Apigenin 7- (2",3"-diacetylglucoside)	4,5-dicaffeoylquinic acid;3,5-dicaffeoylquinic acid;3,4-di-O-caffeoylquinic acid;1,3-di-O-caffeoylquinic acid	4,5-Dicaffeoylquinic acid (90.0%)	Dicaffeoylqunic acid	3
526.14751_4.721	C27 H26 O11	MFCD01959021;MFCD01942790;MFCD03295620;MFCD05151752;MFCD03533862;MFCD04573100;MFCD03937200;MFCD09867869;MFCD09868215;2,4-Bis[2-(phenylsulfonyl)ethyl]dodecahydrooxireno[4',5']pentaleno[2',1',6':3,4,5]pentaleno[1,2-b]oxirene	Viscutin 1			Viscutin 1	3

Feature	Predicted Formula	MS1 matching			MS2 Matching	Putative Identified Compound	MSI
		ChemiSpider	Flavonoid Mass list	*Convolvulaceae* Mass list	mzCloud		
530.14304_4.313	C26 H26 O12	(1S,3R,4R,5R)-3-{[(2E)-3-(3,4-Dihydroxyphenyl)-2-propenoyl]oxy}-4,5-dihydroxy-1-{[(2E)-3-(4-hydroxy-3-methoxyphenyl)-2-propenoyl]oxy}cyclohexanecarboxylic acid;(1S,3R,4R,5R)-1-{[(2E)-3-(3,4-Dihydroxyphenyl)-2-propenoyl]oxy}-3,4-dihydroxy-5-{[(2E)-3-(4-hydroxy-3-methoxyphenyl)-2-propenoyl]oxy}cyclohexanecarboxylic acid	Luteolin 3'-methyl ether 7- (6"-crotonylglucoside);Luteolin 7-glucoside-4'- (Z-2-methyl-2-butenoate)			Flavonoid glycoside derivatives	3
530.14304_3.973	C26 H26 O12	(1S,3R,4R,5R)-3-{[(2E)-3-(3,4-Dihydroxyphenyl)-2-propenoyl]oxy}-4,5-dihydroxy-1-{[(2E)-3-(4-hydroxy-3-methoxyphenyl)-2-propenoyl]oxy}cyclohexanecarboxylic acid;(1S,3R,4R,5R)-1-{[(2E)-3-(3,4-Dihydroxyphenyl)-2-propenoyl]oxy}-3,4-dihydroxy-5-{[(2E)-3-(4-hydroxy-3-methoxyphenyl)-2-propenoyl]oxy}cyclohexanecarboxylic acid	Luteolin 3'-methyl ether 7- (6"-crotonylglucoside);Luteolin 7-glucoside-4'- (Z-2-methyl-2-butenoate)			Flavonoid glycoside derivatives	3
530.14305_3.717	C26 H26 O12	(1S,3R,4R,5R)-3-{[(2E)-3-(3,4-Dihydroxyphenyl)-2-propenoyl]oxy}-4,5-dihydroxy-1-{[(2E)-3-(4-hydroxy-3-methoxyphenyl)-2-propenoyl]oxy}cyclohexanecarboxylic acid;(1S,3R,4R,5R)-1-{[(2E)-3-(3,4-Dihydroxyphenyl)-2-propenoyl]oxy}-3,4-dihydroxy-5-{[(2E)-3-(4-hydroxy-3-methoxyphenyl)-2-propenoyl]oxy}cyclohexanecarboxylic acid	Luteolin 3'-methyl ether 7- (6"-crotonylglucoside);Luteolin 7-glucoside-4'- (Z-2-methyl-2-butenoate)			Flavonoid glycoside derivatives	3
535.09984_3.341	C16 H23 N7 O10 P2	MFCD03294823;MFCD03302834					4
1070.20008_3.165	C43 H53 N4 O18 P3 S2						4

[1] Putative identified compounds were determined as level 1-4 according to Metabolomicsc Standard Initiative(MSI).

[2] Compounds with MSI level 2 that were putatively annotated based on spectral similarity to available databases.

[3] Compounds with MSI level 3 that were putatively characterized their compound classes according to spectral similarity to known compounds of a chemical class.

[4] Unknown compounds with MSI level 4 that are differentiable from spectra data but unclassified or unidentified based on MS and MS/MS data.

2. Metabolite identification of features contributed to the discrimination between F3:EtOH/EtOAc and F5:EtOH/H2O fractions in ESI negative mode.

Feature	Predicted Formula	MS1 matching			MS2 Matching	Putative Identified Compound	MSI
		ChemiSpider	Flavonoid Mass list	*Convolvulaceae* Mass list	mzCloud		
250.06648_3.766	C13 H14 O3 S	MFCD09869012					4
283.12085_3.847	C17 H17 N O3	8394939;trans-N-p-coumaroyl tyramine;N-FMOC-ETHANOLAMINE;Coumarin 334;BW A4C;2-(4-Acetylphenoxy)-N-o-tolylacetamide;MFCD00095390;N-(Phenylacetyl)phenylalanine;MFCD00739392;MFCD01071884;MFCD05874952;MFCD00412027;MFCD00522899;MFCD00745004;MFCD01181533;MFCD01181534;morphinone;MFCD01181532;N-(Phenylacetyl)phenylalanine;MFCD01340309;MFCD01216046;MFCD01195147;MFCD01213206;MFCD01195142;Heclin;MFCD01195072;MFCD01195168;MFCD01195146;KS 370G;MFCD00414766;3-(1,3-Benzodioxol-5-ylamino)-1-(4-methylphenyl)-1-propanone;MFCD03720166;MFCD18430430;MFCD02668157;MFCD02668144;MFCD18429680;MFCD00096249;MFCD18430130;MFCD00667688;MFCD00032221;MFCD00032409;MFCD12974141;MFCD00091186;MFCD18430065;MFCD03414231;MFCD03937598;MFCD18430061;MFCD02685067;MFCD18430060;MFCD01179536;MFCD18430138;MFCD18430131			(2E)-3-(4-Hydroxyphenyl)-N-[2-(4-hydroxyphenyl)ethyl]acrylamide (87.0%)	(2E)-3-(4-Hydroxyphenyl)-N-[2-(4-hydroxyphenyl)ethyl] acrylamide	2
288.23061_6.625	C16 H32 O4	Glaurin;MFCD01863712;Methyl 3-(decyloxy)-2-ethoxypropanoate;2-[2-(Decyloxy)ethoxy]ethyl acetate					4
313.13132_4.124	C18 H19 N O4	kresoxim-methyl;BTEE;Moupinamide;DG8545700;Coumarin 314;3217335;2768622;MFCD03426358;MFCD00783884;MFCD01185169;MFCD00487301;MFCD01009000;MFCD01034065;3-(2,3-Dihydro-1,4-benzodioxin-6-ylamino)-1-(4-methoxyphenyl)-1-propanone;MFCD00175033;MFCD00522919;MFCD00091476;Methyl N-(4-methoxybenzoyl)phenylalaninate;MFCD00221994;MFCD01180475;MFCD01009325;MFCD01007907;MFCD02360807;MFCD00522712;1-(1,3-benzodioxol-5-yl)-3-(4-ethoxyanilino)-1-propanone;MFCD01034066;MFCD01038401;MFCD02363314;MFCD01878254;MFCD01612152;MFCD02366924;MFCD01185352;MFCD02080744;MFCD06804568;MFCD02363303;MFCD00396842;MFCD01032744;MFCD00091477;MFCD02668917;MFCD00685289;MFCD03418827;MFCD00027910;Methyl N-(2-methoxybenzoyl)phenylalaninate;MFCD00032267;MFCD00094657;MFCD02804903;MFCD02668140;MFCD03936365;Methyl N-(3-methoxybenzoyl)phenylalaninate;MFCD00094666;MFCD03937601;MFCD03937600;MFCD09865410;1-(Bicyclo[2.2.1]hept-2-en-2-yl)-3-buten-2-yl 4-nitrobenzoate;14-Hydroxy-3-methoxy-17-methyl-7,8-didehydro-4,5-epoxymorphinan-6-one;(5S,7bS)-2,2a,3,4,4a,5,7a,7b-Octahydro-1H-cyclopenta[cd]inden-5-yl 4-nitrobenzoate;RCL R543721					4
328.22551_6.388	C18 H32 O5	(10E,15Z)-9,12,13-Trihydroxy-10,15-octadecadienoic acid;MFCD00087221;MFCD03931398			Corchorifatty acid F (90.2%)	Corchorifatty acid F	2
328.22573_6.293	C18 H32 O5	(10E,15Z)-9,12,13-Trihydroxy-10,15-octadecadienoic acid;MFCD00087221;MFCD03931398			Corchorifatty acid F (86.5%)	Corchorifatty acid F	2
330.24103_6.566	C18 H34 O5	15-(2-Methyl-2-propanyl)tetradecahydro-1,4,7,10,13-benzopentaoxacyclopentadecine;(9Z)-12,13,17-Trihydroxy-9-octadecenoic acid;1-Hydroxy-1,2-ethanediyl dioctanoate					4
370.22629_5.188	C20 H35 O4 P	bis[4-(pentyloxy)phenyl]diazene oxide;MFCD30718315					4
392.11154_2.845	C19 H20 O9	MFCD03936902;MFCD00614496;MFCD00614570;MFCD18428612					4
432.10630_3.741	C21 H20 O10	5-Hydroxy-3-(4-hydroxyphenyl)-4-oxo-4H-chromen-7-yl hexopyranoside;Apigetrin;beta-D-Genistin;vitexin;Afzelin;Isovitexin;MFCD01725365;Baicalein 7-b-D-glucopyranoside;Genistin;MFCD00275821;MFCD00228892;Sophoricoside;3-(4-Hydroxyphenyl)-4-oxo-3,4-dihydro-2H-chromen-7-yl beta-D-glucopyranosiduronic acid;(1S)-1,5-Anhydro-1-[5,7-dihydroxy-3-(4-hydroxyphenyl)-4-oxo-4H-chromen-6-yl]-D-glucitol;(2Z)-2-(3,4-Dihydroxybenzylidene)-3-oxo-2,3-dihydro-1-benzofuran-6-yl beta-D-glucopyranoside;3-(beta-D-Glucopyranosyloxy)-7-hydroxy-2-(4-hydroxyphenyl)-5-chromeniumolate;5-[5-(beta-D-Glucopyranosyloxy)-7-hydroxy-2-chromeniumyl]-2-hydroxyphenolate;4-{[(Z)-(6-Hydroxy-3-oxo-1-benzofuran-2(3H)-ylidene)methyl]oxy}phenyl beta-D-glucopyranoside	Luteolin 3'-methyl ether 7-xyloside;Kaempferide 3-alpha-L-arabinopyranoside;3'-Hydroxy-3,5,8,4',5'-pentamethoxy-6,7-methylenedioxyflavone;8-C-Glucosyl-5-deoxykaempferol;Resokaempferol 3-glucoside;Resokaempferol 7-glucoside;Resokaempferol 4'-glucoside;Galanginin;Galangin 7-glucoside;Afzelin;Kaempferol 5-rhamnoside;Kaempferol 7-rhamnoside;FL3FADCS0022_a;Kaempferol 4'-rhamnoside;6,7,4'-Trihydroxyisoflavone 4'-O-glucoside;Isogenistein 7-O-glucoside;Genistein 8-C-glucoside;Genistin;Genistein 4'-O-glucoside;Genistein 5-O-glucoside;Pueraria glycoside 1;Luteolin 3'-rhamnoside;8-C-Rhamnopyranosylluteolin;Sulfurein;Aureusidin 6-rhamnoside;7,3',4'-Trihydroxyflavone 7-glucoside;7,3',4'-Trihydroxyflavone 7-galactoside;7,3',4',5'-Tetrahydroxyflavone 7-rhamnoside;5,7,2'-Trihydroxy 7-glucoside;Vitexin;8-C-beta-D-Galactopyranosylapigenin;6-C-beta-D-Galactosylapigenin;Luteolin 7-rhamnoside;Neovitexin;Apigenin 7-O-glucoside;Isovitexin;Apigenin 5-glucoside;Apigenin 5-galactoside;Apigenin 7-galactoside;Apigenin 4'-glucoside;6-C-Chinovopyranosylluteolin;6-C-Fucopyranosylluteolin;Baicalein 6-glucoside;Scutellarein 7-rhamnoside;5,7,8-Trihydroxyflavone 5-glucoside;5,7,8-Trihydroxyflavone 7-galactoside;FL3FAACS0092_a		Afzelin (86.8%)	Flavonoid glycosides	3

Feature	Predicted Formula	MS1 matching			MS2 Matching	Putative Identified Compound	MSI
		ChemiSpider	Flavonoid Mass list	*Convolvulaceae* Mass list	mzCloud		
434.08560_ 3.072	C20 H18 O11	Avicularin;Quercetin 3-O-.alpha.-L-arabinopyranside;6023007;MFCD00275850	6-Hydroxyluteolin 6-xyloside;6-Hydroxyluteolin 7-arabinopyranoside;6-Hydroxyluteolin 7-xyloside;6-Hydroxyluteolin 7-apioside;8-Hydroxyluteolin 7-xyloside;Tricetin 3'-xyloside;Isoetin 2'-xyloside;Guaijaverin;Quercetin 3-beta-L-arabinopyranoside;Reynoutrin;Quercetin 7-xyloside;Quercetin 3'-xyloside;Quercetin 3-O-alpha-D-arabinopyranoside;Herbacetin 7-beta-L-arabinopyranoside;Herbacetin 8-alpha-L-arabinopyranoside;Herbacetin 8-xyloside;Avicularin			Flavonoid glycosides	3
448.10088_ 3.492	C21 H20 O11	Quercitrin;Astragalin;Cynaroside;Orientin;Isoorientin;kaempferol 7-O-beta-D-glucopyranoside;Trifolin;Naringenin 7-O-beta-D-glucuronide;4-(5,7-Dihydroxy-4-oxo-3,4-dihydro-2H-chromen-2-yl)phenyl beta-D-glucopyranosiduronic acid;MFCD13186795;aureusidin 6-O-beta-glucoside;7-Hydroxy-2-(4-hydroxyphenyl)-4-oxo-3,4-dihydro-2H-chromen-5-yl beta-D-glucopyranosiduronic acid;Kaempferol 3-O-D-galactoside;cyanidin 3-O-beta-D-glucoside betaine;cyanidin 3-O-beta-D-galactoside betaine;2-(3,4-Dihydroxyphenyl)-3-(hexopyranosyloxy)-7-hydroxy-5-chromeniumolate;5-[3-(D-Glucopyranosyloxy)-5,7-dihydroxy-2-chromeniumyl]-2-hydroxyphenolate	Carthamone;Dihydrobaicalein 7-O-glucuronide;Dihydronorwogonin 7-O-glucuronide;evolvuside B;5,7,2',6'-Tetrahydroxyflavone 2'-O-glucoside;Isoorientin;Orientin;6-C-Galactosylluteolin;Scutellarein 6-glucoside;Scutellarein 7-glucoside;6-Hydroxyluteolin 5-rhamnoside;6-Hydroxyluteolin 7-rhamnoside;Aureusin;6-Hydroxyluteolin 6-rhamnoside;Isoscutellarein 7-glucoside;Hypolaetin 8-rhamnoside;8-C-Galactosylluteolin;Galuteolin;Luteolin 5-galactoside;Luteolin-7-glucoside;Luteolin 7-galactoside;Luteolin 3'-glucoside;Luteolin 4'-glucoside;8-C-Methylquercetin 3-xyloside;Quercitrin;Quercetin 7-rhamnoside;6-C-Galactosylisoscutellarein;Isorhamnetin 3-alpha-L-arabinofuranoside;Cernuoside;Maritimein;Maritimetin 7-glucoside;Distichin;Herbacetin 7-rhamnoside;Isorhamnetin 3-xyloside;Herbacetin 8-rhamnoside;8-C-Glucosylfisetin;Fisetin 3-glucoside;Fisetin 7-glucoside;Fisetin 4'-glucoside;Datiscanin;6-C-Glucopyranosylkaempferol;8-C-beta-D-Glucopyranosylkaempferol;Kaempferol 3-alpha-D-galactoside;Trifolin;Astragalin;Kaempferol 3-alpha-D-glucoside;Asiaticalin;Kaempferol 7-alloside;Kaempferol 7-galactoside;Populnin;Kaempferol 4'-glucoside;Rhamnetin 3-alpha-L-arabinofuranoside;Rhamnetin 3-alpha-L-arabinopyranoside;Quercetin 3-methyl ether 3'-xyloside;8-C-Glucosylorobol;6-C-Glucosylorobol;Orobol 7-O-glucoside;5,7,3',4'-Tetrahydroxy-4-phenylcoumarin 5-O-glucoside;Kaempferol 5-glucoside	lutoelin-7-O-D-glucoside	Cynaroside (87.7%);Kuromanin (86.6%);5,7-dihydroxy-2-(3-hydroxy-4-{[(2S,3R,4S,5S,6R)-3,4,5-trihydroxy-6-(hydroxymethyl)oxan-2-yl]oxy}phenyl)-4H-chromen-4-one (83.4%);Quercitrin (82.9%);2-(3,4-dihydroxyphenyl)-5,7-dihydroxy-3-{[(2S,3R,4R,5R,6S)-3,4,5-trihydroxy-6-methyloxan-2-yl]oxy}-4H-chromen-4-one (81.3%);Kaempferol-7-O-glucoside (79.9%);Trifolin (78.3%)	Flavonoid glycosides	3
**448.10108_ 3.251**	C21 H20 O11	Quercitrin;Astragalin;Cynaroside;Orientin;Isoorientin;kaempferol 7-O-beta-D-glucopyranoside;Trifolin;Naringenin 7-O-beta-D-glucuronide;4-(5,7-Dihydroxy-4-oxo-3,4-dihydro-2H-chromen-2-yl)phenyl beta-D-glucopyranosiduronic acid;MFCD13186795;aureusidin 6-O-beta-glucoside;7-Hydroxy-2-(4-hydroxyphenyl)-4-oxo-3,4-dihydro-2H-chromen-5-yl beta-D-glucopyranosiduronic acid;Kaempferol 3-O-D-galactoside;cyanidin 3-O-beta-D-glucoside betaine;cyanidin 3-O-beta-D-galactoside betaine;2-(3,4-Dihydroxyphenyl)-3-(hexopyranosyloxy)-7-hydroxy-5-chromeniumolate;5-[3-(D-Glucopyranosyloxy)-5,7-dihydroxy-2-chromeniumyl]-2-hydroxyphenolate	Carthamone;Dihydrobaicalein 7-O-glucuronide;Dihydronorwogonin 7-O-glucuronide;evolvuside B;5,7,2',6'-Tetrahydroxyflavone 2'-O-glucoside;Isoorientin;Orientin;6-C-Galactosylluteolin;Scutellarein 6-glucoside;Scutellarein 7-glucoside;6-Hydroxyluteolin 5-rhamnoside;6-Hydroxyluteolin 7-rhamnoside;Aureusin;6-Hydroxyluteolin 6-rhamnoside;Isoscutellarein 7-glucoside;Hypolaetin 8-rhamnoside;8-C-Galactosylluteolin;Galuteolin;Luteolin 5-galactoside;Luteolin-7-glucoside;Luteolin 7-galactoside;Luteolin 3'-glucoside;Luteolin 4'-glucoside;8-C-Methylquercetin 3-xyloside;Quercitrin;Quercetin 7-rhamnoside;6-C-Galactosylisoscutellarein;Isorhamnetin 3-alpha-L-arabinofuranoside;Cernuoside;Maritimein;Maritimetin 7-glucoside;Distichin;Herbacetin 7-rhamnoside;Isorhamnetin 3-xyloside;Herbacetin 8-rhamnoside;8-C-Glucosylfisetin;Fisetin 3-glucoside;Fisetin 7-glucoside;Fisetin 4'-glucoside;Datiscanin;6-C-Glucopyranosylkaempferol;8-C-beta-D-Glucopyranosylkaempferol;Kaempferol 3-alpha-D-galactoside;Trifolin;Astragalin;Kaempferol 3-alpha-D-glucoside;Asiaticalin;Kaempferol 7-alloside;Kaempferol 7-galactoside;Populnin;Kaempferol 4'-glucoside;Rhamnetin 3-alpha-L-arabinofuranoside;Rhamnetin 3-alpha-L-arabinopyranoside;Quercetin 3-methyl ether 3'-xyloside;8-C-Glucosylorobol;6-C-Glucosylorobol;Orobol 7-O-glucoside;5,7,3',4'-Tetrahydroxy-4-phenylcoumarin 5-O-glucoside;Kaempferol 5-glucoside	lutoelin-7-O-D-glucoside	Astragalin (82.4%) ; Quercitrin (83.5%)	Quercitrin (Quercetin-3-O-rhamnoside)	2
448.10124_ 3.392	C21 H20 O11	Quercitrin;Astragalin;Cynaroside;Orientin;Isoorientin;kaempferol 7-O-beta-D-glucopyranoside;Trifolin;Naringenin 7-O-beta-D-glucuronide;4-(5,7-Dihydroxy-4-oxo-3,4-dihydro-2H-chromen-2-yl)phenyl beta-D-glucopyranosiduronic acid;MFCD13186795;aureusidin 6-O-beta-glucoside;7-Hydroxy-2-(4-hydroxyphenyl)-4-oxo-3,4-dihydro-2H-chromen-5-yl beta-D-glucopyranosiduronic acid;Kaempferol 3-O-D-galactoside;cyanidin 3-O-beta-D-glucoside betaine;cyanidin 3-O-beta-D-galactoside betaine;2-(3,4-Dihydroxyphenyl)-3-(hexopyranosyloxy)-7-hydroxy-5-chromeniumolate;5-[3-(D-Glucopyranosyloxy)-5,7-dihydroxy-2-chromeniumyl]-2-hydroxyphenolate	Carthamone;Dihydrobaicalein 7-O-glucuronide;Dihydronorwogonin 7-O-glucuronide;evolvuside B;5,7,2',6'-Tetrahydroxyflavone 2'-O-glucoside;Isoorientin;Orientin;6-C-Galactosylluteolin;Scutellarein 6-glucoside;Scutellarein 7-glucoside;6-Hydroxyluteolin 5-rhamnoside;6-Hydroxyluteolin 7-rhamnoside;Aureusin;6-Hydroxyluteolin 6-rhamnoside;Isoscutellarein 7-glucoside;Hypolaetin 8-rhamnoside;8-C-Galactosylluteolin;Galuteolin;Luteolin 5-galactoside;Luteolin-7-glucoside;Luteolin 7-galactoside;Luteolin 3'-glucoside;Luteolin 4'-glucoside;8-C-Methylquercetin 3-xyloside;Quercitrin;Quercetin 7-rhamnoside;6-C-Galactosylisoscutellarein;Isorhamnetin 3-alpha-L-arabinofuranoside;Cernuoside;Maritimein;Maritimetin 7-glucoside;Distichin;Herbacetin 7-rhamnoside;Isorhamnetin 3-xyloside;Herbacetin 8-rhamnoside;8-C-Glucosylfisetin;Fisetin 3-glucoside;Fisetin 7-glucoside;Fisetin 4'-glucoside;Datiscanin;6-C-Glucopyranosylkaempferol;8-C-beta-D-Glucopyranosylkaempferol;Kaempferol 3-alpha-D-galactoside;Trifolin;Astragalin;Kaempferol 3-alpha-D-glucoside;Asiaticalin;Kaempferol 7-alloside;Kaempferol 7-galactoside;Populnin;Kaempferol 4'-glucoside;Rhamnetin 3-alpha-L-arabinofuranoside;Rhamnetin 3-alpha-L-arabinopyranoside;Quercetin 3-methyl ether 3'-xyloside;8-C-Glucosylorobol;6-C-Glucosylorobol;Orobol 7-O-glucoside;5,7,3',4'-Tetrahydroxy-4-phenylcoumarin 5-O-glucoside;Kaempferol 5-glucoside	lutoelin-7-O-?-D-glucoside	Astragalin (87.6%);Kaempferol-7-O-glucoside (78.7%)	Flavonoid glycosides	3

Feature	Predicted Formula	MS1 matching			MS2 Matching	Putative Identified Compound	MSI
		ChemiSpider	Flavonoid Mass list	*Convolvulaceae* Mass list	mzCloud		
452.11113_ 3.673	C24 H20 O9	foramsulfuron;MFCD01650235;MFCD01650135;MFCD04442468;MFCD11975230;MFCD05151016	Epigallocatechin 3-O-p-coumarate;Cinchonain Ic;Cinchonain Id;9,10-Dihydro-10- (3,4-dihydroxyphenyl) -pyrano [2,3-h] catechin-8-one;Cinchonain Ib			Flavan-3-ol derivatives	3
453.11447_ 3.215	C16 H20 N7 O7 P	MFCD02654785;MFCD03225697;MFCD03494474;MFCD03285415;MFCD05720973					4
476.22668_ 7.876	C23 H32 N4 O7	Falgpa					4
495.10201_ 3.599	C22 H17 N5 O9	MFCD02330324;MFCD03224434;MFCD03223195;MFCD03473529;MFCD03223194;MFCD03089445					4
498.11685_ 4.286	C26 H18 N4 O7	MFCD00767685;MFCD02726012					4
498.1171_3 .862	C26 H18 N4 O7	MFCD00767685;MFCD02726012					4
498.11711_ 3.793	C22 H14 N10 O5	MFCD03533804;MFCD03141359;MFCD00767685;MFCD04525290;MFCD02726012;MFCD02831722;MFCD02842048;MFCD11055154;MFCD09869300					4
500.13232_ 3.767	C25 H24 O11	MFCD00667386	Apigenin 7- (6"-crotonylglucoside); Epigallocatechin 5,3',5'-trimethyl ether 3-O-gallate;Puerarin 4',6"-diacetate				4
500.13278_ 3.465	C25 H24 O11	MFCD03010405;MFCD03296217;MFCD03301384;MFCD03284168;MFCD01793233;MFCD00667386;RN-9893;RCL T224529	Apigenin 7- (6"-crotonylglucoside); Epigallocatechin 5,3',5'-trimethyl ether 3-O-gallate;Puerarin 4',6"-diacetate				4
**516.12723_ 3.168**	C25 H24 O12	Cynarine;MFCD22375481;3,4-Dicaffeoylquinic acid;Cynarin;3,5-O-dicaffeoylquinic acid	2",6"-Di-O-Acetyl isovitexin;Apigenin 7- (2",3"-diacetylglucoside);Apigenin 7- (3",4"-diacetylglucoside);Kaempferol 3- (2",4"-diacetylrhamnoside);Kaempferol 3- (3",4"-diacetylrhamnoside);Formononetin 7-O- (6"-malonylglucoside)	1,3-di-O-caffeoylquinic acid;3,4-di-O-caffeoylquinic acid;3,5-dicaffeoylquinic acid;4,5-dicaffeoylquinic acid	4,5-Dicaffeoylquinic acid (90.0%)	Dicaffeoylqunic acid	3
530.14304_ 4.313	C26 H26 O12	(1S,3R,4R,5R)-3-{[(2E)-3-(3,4-Dihydroxyphenyl)-2-propenoyl]oxy}-4,5-dihydroxy-1-{[(2E)-3-(4-hydroxy-3-methoxyphenyl)-2-propenoyl]oxy}cyclohexanecarboxylic acid;(1S,3R,4R,5R)-1-{[(2E)-3-(3,4-Dihydroxyphenyl)-2-propenoyl]oxy}-3,4-dihydroxy-5-{[(2E)-3-(4-hydroxy-3-methoxyphenyl)-2-propenoyl]oxy}cyclohexanecarboxylic acid	Luteolin 7-glucoside-4'- (Z-2-methyl-2-butenoate);Luteolin 3'-methyl ether 7- (6"-crotonylglucoside)			Flavonoid glycoside derivatives	3
530.14304_ 3.973	C26 H26 O12	(1S,3R,4R,5R)-3-{[(2E)-3-(3,4-Dihydroxyphenyl)-2-propenoyl]oxy}-4,5-dihydroxy-1-{[(2E)-3-(4-hydroxy-3-methoxyphenyl)-2-propenoyl]oxy}cyclohexanecarboxylic acid;(1S,3R,4R,5R)-1-{[(2E)-3-(3,4-Dihydroxyphenyl)-2-propenoyl]oxy}-3,4-dihydroxy-5-{[(2E)-3-(4-hydroxy-3-methoxyphenyl)-2-propenoyl]oxy}cyclohexanecarboxylic acid	Luteolin 7-glucoside-4'- (Z-2-methyl-2-butenoate);Luteolin 3'-methyl ether 7- (6"-crotonylglucoside)			Flavonoid glycoside derivatives	3
530.14305_ 3.717	C26 H26 O12	(1S,3R,4R,5R)-3-{[(2E)-3-(3,4-Dihydroxyphenyl)-2-propenoyl]oxy}-4,5-dihydroxy-1-{[(2E)-3-(4-hydroxy-3-methoxyphenyl)-2-propenoyl]oxy}cyclohexanecarboxylic acid;(1S,3R,4R,5R)-1-{[(2E)-3-(3,4-Dihydroxyphenyl)-2-propenoyl]oxy}-3,4-dihydroxy-5-{[(2E)-3-(4-hydroxy-3-methoxyphenyl)-2-propenoyl]oxy}cyclohexanecarboxylic acid	Luteolin 7-glucoside-4'- (Z-2-methyl-2-butenoate);Luteolin 3'-methyl ether 7- (6"-crotonylglucoside)			Flavonoid glycoside derivatives	3
530.14308_ 3.913	C26 H26 O12	(1S,3R,4R,5R)-3-{[(2E)-3-(3,4-Dihydroxyphenyl)-2-propenoyl]oxy}-4,5-dihydroxy-1-{[(2E)-3-(4-hydroxy-3-methoxyphenyl)-2-propenoyl]oxy}cyclohexanecarboxylic acid;(1S,3R,4R,5R)-1-{[(2E)-3-(3,4-Dihydroxyphenyl)-2-propenoyl]oxy}-3,4-dihydroxy-5-{[(2E)-3-(4-hydroxy-3-methoxyphenyl)-2-propenoyl]oxy}cyclohexanecarboxylic acid	Luteolin 7-glucoside-4'- (Z-2-methyl-2-butenoate);Luteolin 3'-methyl ether 7- (6"-crotonylglucoside)			Flavonoid glycoside derivatives	3
544.1587_5 .275	C28 H24 N4 O8	MFCD02672529;(1S,3R,4R,5R)-3,4-Dihydroxy-1,5-bis{[(2E)-3-(4-hydroxy-3-methoxyphenyl)-2-propenoyl]oxy}cyclohexanecarboxylic acid					4
544.15876_ 4.435	C29 H20 N8 O4	MFCD02672529;(1S,3R,4R,5R)-3,4-Dihydroxy-1,5-bis{[(2E)-3-(4-hydroxy-3-methoxyphenyl)-2-propenoyl]oxy}cyclohexanecarboxylic acid					4
544.15887_ 4.713	C28 H24 N4 O8	MFCD02672529					4
570.13851_ 3.609	C23 H23 N8 O8 P						4
678.15954_ 4.308	C34 H30 O15			3,4,5-tri-O-caffeoylquinic acid		3,4,5-tri-O-caffeoylquinic acid	3
897.20656_ 3.385	C37 H41 N9 O12 P2 S						4
1032.25617 3.168	C52 H40 N8 O16						4

[1] Putative identified compounds were determined as level 1-4 according to Metabolomicsc Standard Initiative(MSI).

[2] Compounds with MSI level 2 that were putatively annotated based on spectral similarity to available databases.

[3] Compounds with MSI level 3 that were putatively characterized their compound classes according to spectral similarity to known compounds of a chemical class.

[4] Unknown compounds with MSI level 4 that are differentiable from spectra data but unclassified or unidentified based on MS and MS/MS data.
