# Supplementary material for: LC-MS/MS metabolomics-facilitated identification of the active compounds responsible for anti-allergic activity of the ethanol extract of Xenostegia tridentata
Source: PLoS One. 2022 Apr 15;17(4):e0265505. doi: 10.1371/journal.pone.0265505 (PMC9012362; doi:10.1371/journal.pone.0265505)
Supplement: Attachment — Submitted filename: Response to Reviewers.pdf [file pone.0265505.s006.pdf]

Response to reviewers

PONE-D-21-29347

LC-MS/MS metabolomics-facilitated identification of the active compounds responsible for anti-allergic activity of the ethanol extract of *Xenostegia tridentata*

Journal Requirements:

1. Please ensure that your manuscript meets PLOS ONE's style requirements, including those for file naming. The PLOS ONE style templates can be found at https://journals.plos.org/plosone/s/file?id=wjVg/PLOSOne_formatting_sample_main_body.pdf and https://journals.plos.org/plosone/s/file?id=ba62/PLOSOne_formatting_sample_title_authors_affiliations.pdf

The manuscript is re-formatted to meet PLOS ONE's style requirements**.**

2. Your ethics statement should only appear in the Methods section of your manuscript. If your ethics statement is written in any section besides the Methods, please move it to the Methods section and delete it from any other section. Please ensure that your ethics statement is included in your manuscript, as the ethics statement entered into the online submission form will not be published alongside your manuscript.

The ethics statement is moved into the Materials and methods section.

3. Please include captions for your Supporting Information files at the end of your manuscript, and update any in-text citations to match accordingly. Please see our Supporting Information guidelines for more information: http://journals.plos.org/plosone/s/supporting-information.

By following the guidelines, captions for Supporting Information files are added to the end of the manuscript and the in-text citations are updated.

Author's responses to Reviewers' comments:

1. "Xenostegia tridentata (L.) D.F. Austin & Staples is a synonym of *Merremia tridentata* (L.) Hallier f.", please see below link and correct the statements about the first isolations from the plant (e.g., the last paragraph "for the first time" is not appropriate, because many previous studies of Merrenia were shown, also in the end of Abstract P. 2. Line 15). P. 3 please mention the synonym of the plant. For plant synonyms, please check theplantlist.org. here: http://www.theplantlist.org/tpl1.1/record/tro-8502902

1.1 **The word "also known as *Merremia tridentata* (L.) Hallier f." is added into the Introduction (P.3).**

1.2 Although there have been reports on the presence of many phytochemicals including flavonoids and phenolic compounds in *Merremia tridentata*. To the best of our knowledge, only four flavonoids (diosmetin, luteolin, diosmetin-7-O-β-D-glucoside and luteolin-7-O-β-D-glucoside) have been isolated as pure compounds from the plant.[a] Other reported compounds were just determined by various analytical methods such as GC-MS. In our manuscript, we therefore highlighted that this was the first time for the isolation of 3,5-dicaffeoylquinic acid, quercetin-3-O-rhamnoside and kaempferol-3-O-rhamnoside from the plant. However, by

following the reviewer's comment, **we have removed "for the first time", in the revised version**.

[a] Krishnappa AL, Seetharama TR. Flavonoids of *Merremia tridentata*. Fitoterapia. 1992; 63(2):190

2. Timing of the assays. The errors of the assays, SEM show high error. The viability window for F4 (BuOH) and ketotifen doesn't fit the antigen-induced assay. Ketotifen is approx. 25% toxic at 100uM which would seriously affect the assay response considering sensitivity of antigen-induced assay in RBL cells (unless the timing of treatments was significantly different which is not shown in the Table 1)? Was the samples treatment time in Table 1. For MTT 48 hr and for beta-hexo assay 2hr? Please describe and explain why the cytotoxic samples (including kerotifen at 100uM with over 20% toxicity) were used in antigen-induced beta-hexo assay and the effects of butanol layer are not fitting their toxic levels? Is it because of the different time treatment?

Yes. The treatment times were different. RBL-2H3 cells were incubated with the tested compounds for 2 hr in the antigen-induced assay and for 48 hr in the viability (MTT) assay. The information about incubation times was described in the Materials and methods section. However, in the revised version, **we have added notes about the incubation times under Table1 to clearly show the difference**.

In Table1, we intended to compare the anti-allergic activities of all fractions at the same concentrations and to compare the activity of the extracts with ketotifen fumarate (a known anti-allergic agent) in the same unit (µg/mL). However, at high concentrations, the significant cytotoxicity of EtOH/BuOH (F4) was observed (for 48-hour incubation time). We therefore discussed that its cytotoxicity presumably was the reason that made the activity of F4 in degranulation assay not in dose-dependent manner.

According to the reviewer's comments, we agree that the activity at the toxic concentrations should not be reported. To see if crude EtOH (F1), EtOH/BuOH (F4) and ketotifen fumarate (100 µg/mL) are toxic to RBL-2H3 cells at 2-hr incubation time, we have performed MTT assay during this revision process. The result is shown in the below table. F1 showed 1.4 and 6.2 %toxicity at 500 µg/mL and 1000 µg/mL, respectively. However, at 2000 µg/mL, the cytotoxicity was more than 20%. F4 displayed 2.8% toxicity at the 500 µg/mL and over 20% toxicity at the concentrations of 1000 and 2000 µg/mL. Ketotifen fumarate at 100 µg/mL showed 5.4% toxicity.

Cytotoxicity of F1:crude, F4:EtOH/BuOH and ketotifen fumarate against RBL-2H3 cells (2-hr incubation)

sample	concentration	%cytotoxicity
F1: Crude EtOH	500 µg/mL<br>1000 µg/mL<br>2000 µg/mL	1.4±1.0<br>6.2±0.8<br>31.3±6.1
F4: EtOH/BuOH	500 µg/mL<br>1000 µg/mL<br>2000 µg/mL	2.8±1.2<br>23.1±0.9<br>58.0±0.4
Ketotifen fumarate	100 µg/mL	5.4±0.2

Consequently, in the revised version, **we have removed the inhibitory activity results of F1: Crude EtOH at 2000 µg/mL and F4: EtOH/BuOH at 1000 and 2000 µg/mL from Table1. The discussion also has been corrected accordingly.**

In the revised manuscript, Page13, lines 3-14

“As shown in Table 1, similar to the effect of ketotifen fumarate (a known anti-allergic agent), the F1-F3 extracts possessed mast cell degranulation inhibitory activity on IgE-mediated in a dose-dependent manner. Among them, the ethyl acetate fraction (F3) exhibited the highest anti-allergic activity and showed improved activity over the crude ethanol extract (F1), without being cytotoxic to the cells at the concentration up to 2000 µg/mL. The butanol fraction (F4) showed weak activity at 500 µg/mL and was over 20% toxic to the cells at the higher concentrations. The water fraction (F5) also exerted weak activity and did not inhibit the degranulation in dose-dependent manner. Markedly, at the concentrations of 1000 and 2000 µg/mL, F3 exhibited significantly higher inhibitory activity than F5: EtOH/H2O ($p<0.001$). Moreover, to assure that the observed effects of the extracts on the inhibition of mast cell degranulation were not from the inhibition of the enzyme activity, β-hexosaminidase inhibitory activity of each extract was determined. The results showed that no significant inhibition was observed for all tested samples (see S3 Table).”

3. n/a

4. In the procedure, long incubation times may cause statistical errors particularly in the antigen induced assays.

From literature search, the incubation times in the antigen-induced assays were quite varied (from 10-min[a] to 20-hr[b] incubation time). Although 1-hr incubation seemed to be the most common one. In our optimization step, we found more significant activity when performed at 2-hr incubation. So, all the results reported in this manuscript were from the incubation of the sensitized cells with tested compounds for 2 hr prior to the stimulation.

[a]Scientific Reports, **2018**, *8*, 11638.

[b]Frontiers in Pharmacology, **2021**, *12*, 674095.

5. P. 6. Line 10 Was the solubility of extracts in tween saline solution good enough? Was the sample filtered before administration? Was the solvent and vehicle the same? Please describe clearly.

Tween is an emulsifier widely used in the food industry and it is compatible with our treatment application by oral route. Since the crude ethanol extract contained some non-polar compounds, tween saline solution was used as the solvent to enhance the solubility of the extract. The prepared solution was then filtrated before administration. The solvent (tween saline solution) was used in the vehicle control group. **In the revised manuscript, we have added more detail about the sample preparation and the solvent used in the vehicle control group for the passive cutaneous anaphylaxis assay in Materials and methods section**.

In the revised manuscript, Page6, lines 9-22

"Experiments were performed on 7-8-week-old adult ICR mice (Siam Nomura, Thailand). The experiments were carried out in Center for Animal Research, Naresuan University. To induce passive cutaneous anaphylaxis reaction, ears of ICR mice were intradermally injected with 0.5 μg of mouse anti-dinitrophenyl (DNP) IgE antibodies (Sigma Aldrich) or normal saline solution (NSS). After 24 hours**, the filtered crude EtOH extract (F1) or dexamethasone resuspended in 1.2%TWEEN in NSS was orally administered at indicated concentrations. The 1.2%TWEEN in NSS was administered in vehicle control group**. One hour later, mice were intravenously injected with dinitrophenylated human serum albumin (DNP-HSA) 60 μg containing 0.5% Evans blue in phosphate buffer saline (PBS). Mice were observed for another one hour and euthanized. Ears were photographed and removed for Evans blue elution assay. The ears were cut into small pieces and dissolved in 400 μL of formamide at 63 ºC overnight. The resulting supernatant was then collected, and the absorbance was measured at 630 nm using a microplate reader (EnSpire™ Multimode Plate Reader, PerkinElmer, Inc.). Data were presented as absorbance compared to the control group. All efforts were made to minimize suffering and the number of mice needed to reach statistical significance and experimental reproducibility."

6. Isolation scheme and Table 1. Considering that all the samples were oily, and the major fraction was hexane layer (11 g), while ethyl acetate layer was the minor (0.9 g) one. Referring also to Table 4 that content of these compounds is low in crude extract, while 3.034 is high in BuOH. => Is it possible that the most active fraction is hexane layer and maybe due to its unsaturated fatty acid content which exerts strong anti-inflammatory properties? It would be useful to investigate more on the content of this fraction.

Although the major fraction was hexane. From the degranulation assay, we compared the inhibitory activity of each fraction at the same concentrations and found that F3 was significantly more active than other fractions and showed better activity than the crude ethanol extract. This suggested that the most active compounds should present in the F3 fraction. It is possible that the chemical constituents such as unsaturated fatty acids in the hexane fraction could also exert the anti-allergic activity. However, based on the result from the degranulation assay in Table1, their inhibitory activities are not as strong as that of the compounds present in F3.

In Fig 4, although the feature at 3.034 (luteolin-7-O-glucoside) was also abundant in F4 fraction, the amount of this feature found in F4 was significantly less than that found in F3. In addition, the other three features were relatively absent in F4, as compared to in F3. Corresponding to the inhibitory activity of F3 and F4, shown in Table1, the results suggested that the four features including luteolin-7-O-glucoside, quercetin-3-O-rhamnoside, kaempferol-3-O-rhamnoside and 3,5-dicaffeoylquinic acid were important for the activity.

7. Why Compound 48/80 was chosen as inducer, what is its mechanism of action?

compound 48/80 is a non-immune stimulant widely used for studying IgE-independent stimulation of mast cells.[a-c] Particularly, previous works have shown that polyphenol compounds possess inhibitory activity on compound 48/80-induced mast cell degranulation.[d]

We therefore tested the ability of our isolated compounds on the inhibition of compound 48/80-induced degranulation.

compound 48/80 is a mixed polymer of p-methoxy-N-methylphenethylamine cross-linked by formaldehyde.[a] It activates mast cells by interacting with Gα-subunits of trimeric G proteins[e], leading to cell degranulation and promotion of allergic cascade.

[a] Int. J. Mol. Sci. 2021, 22, 10994.
[b] PLOS ONE. 2016, 11(12), e0168120.
[c] Molecules. 2020, 25, 2434.
[d] Anat Cell Biol. 2017, 50, 275-283.
[d] Biol. Pharm. Bull. 2001, 24(9), 1060-1063.

8. P. 21. Line 13 Mention the level of activity, IC50 or values would be useful as well as the comparison with literature values. There was no concentration manner observed from Fig. 5 and 6 for pure compounds activity, is it reasonable I comparison with literature??

Anti-allergic activities of the isolated compounds have been known and reported, as shown in the table below (Table A). In our work, we tested mast cell degranulation inhibitory activity of the isolated compounds to ensure that they really exerted the activity in our assay. Experiments toward better understanding on the anti-allergic effects of the compounds and the extracts of *X.tridentata* are planned to be performed in due course.

In Fig 5 and 6, most compounds showed dose-dependent effects in both IgE-mediated and compound 48/80-stimulated degranulation. Treatment with the compounds at 100 µM showed lower levels of b-hexosaminidase release than at 25 µM. Only for quercetin-3-O-rhamnoside, the effects of the compound at 100 and 25 µM were not different in the compound 48/80-stimulated degranulation assay (Fig 6).

We agree that IC50 values would be useful to compare with literature values. However, from literature search, we found that it's quite hard to compare the activities observed from our assay to the ones reported in the literatures since the detailed protocols from different papers were varied in terms of the incubation times and the amounts of stimulating agents. And, most of them reported the activities in terms of % β-hexosaminidase release or %inhibition rather than IC50 values. We have summarized some of the previously reported inhibitory effects of compounds on antigen-induced release of β-hexosaminidase from RBL-2H3 Cells, as shown in Table B.

Table A. Examples of previously reported anti-allergic activities of the isolated compounds.

compounds	activities	references
**3,5-dicaffeoylquinic acid**	"Glycosylated flavonoids, caffeoylquinic acid and 3,5-dicaffeoylquinic acid have been identified in the ethyl acetate partition from the crude ethanol extract of Tocoyena bullata (Rubiaceae) leaves. The fraction containing the mixture of flavonol rutin and a tetraglycosylated flavonoid showed 89.2% inhibition and the mixture of isoquercitrin and 3,5-dicaffeoylquinic acid showed 88.5% inhibition of mast cell degranulation. **These results demonstrated that the tetraglycosylated flavonoid, rutin, isoquercitrin and 3,5-dicaffeioylquinic acid were the most promising phenolics for inhibition of mast cell degranulation."**	Natural Product Research. 34(22), 3295-3298 (2020)

	Inhibition of β-hexosaminidase release (leukotriene C4/D4/E4 synthesis and TNF-a production) and PCA reaction and suppression of smooth muscle constriction induced by histamine and leukotriene D4	J. Clin. Biochem. Nutr. 67(1), 10–18 (2020)
**luteolin-7-O-glucoside (Cynaroside)**	inhibitory activity on histamine release from rat peritoneal mast cells induced by compound 48/80 (IC50 = 41.6 uM)	Biol. Pharm. Bull. 25(2), 256—259 (2002)
	inhibitory activity on IgE-mediated Anti-allergic activity on RBL-2H3 cell line (IC50 = 470 uM)	CMU.J.Nat.Sci.Special Issue on Agricultural & Natural Resources. 11(1), 343 (2012)
	“Flavonoid glycosides**, luteolin 7-O-glucoside and stechamone, and maltol isolated from S. chamaejasme also showed strong antiallergic effects in hapten-induced dermatitis-like skin lesions in murine models as main components**”	Natural Product Communications. 15(7), 1–7 (2020)
	"Stellera chamaejasme and **Its Main Compound Luteolin 7-O-Glucoside Alleviates Skin Lesions in Oxazolone- and 2,4-DinitrochlorobenzeneStimulated Murine Models of Atopic Dermatitis**"	Planta Med. 85(7), 583-590 (2019)
**quercetin-3-O-rhamnoside (quercitrin)**	"Quercitrin, but not rutin, inhibited OVA-induced contractions of sensitized trachea."	Medicine in Drug Discovery. 12, 100106 (2021)
**kaempferol-3-O-rhamnoside (Afzelin)**	"kaempferol-3-O-rhamnoside fully maintained its anti-inflammatory and anti-asthmatic effects compared with kaempferol in an asthma model mouse. Both kaempferol and kaempferol-3-O-rhamnoside significantly reduced the elevated inflammatory cell numbers in the bronchoalveolar lavage fluid (BALF). kaempferol and kaempferol-3-O-rhamnoside also significantly inhibited the increase in Th2 cytokines (IL-4, IL-5, and IL-13) and TNF-α protein levels through inhibition of the phosphorylation Akt and effectively suppressed eosinophilia in a mouse model of allergic asthma. The total immunoglobulin (Ig) E levels in the serum and BALF were also blocked by kaempferol and kaempferol-3-O-rhamnoside to similar extents. kaempferol-3-O-rhamnoside exerts similar or even slightly higher inhibitory effects on Th2 cytokines and IgE production compared with kaempferol, whereas kaempferol-3-O-rhamnoside was less effective at DPPH radical scavenging and the inhibition of ROS generation in inflammatory cells compared with kaempferol. **These results suggested that the kaempferol-3-O-rhamnoside, as well as kaempferol, may also be a promising candidate for the development of health beneficial foods or therapeutic agents that can prevent or treat allergic asthma**."	International Immunopharmacology. 25(2), 302-310 (2015)

	Afzelin attenuates asthma phenotypes by downregulation of GATA3 in a murine model of asthma. "**Afzelin is promising as a beneficial medication for the treatment of asthma through ameliorating allergic responses**"	Molecular Medicine Reports. 12, 71-76 (2015)

Table B. Previously reported inhibitory effects of compounds on antigen-induced release of β-hexosaminidase from RBL-2H3 Cells

**compound**	**concentration**	**%inhibition**	**references**
**ketotifen fumarate**	25 ug/mL (~58 uM)	20	Chemistry of Natural Compounds. 52(6), 1089–1092 (2016)
	50 ug/mL (~117 uM)	49	
	35 uM	31	International Immunopharmacology. 59, 113–119 (2018)
	15.62 ug/mL (36 uM)	60	BMC Complementary and Alternative Medicine. 19, 361(2019)
	35 uM	60	Evidence-Based Complementary and Alternative Medicine. 2020, Article ID 4307161 (2020)
**luteolin-7-O-glucoside (Cynaroside)**	25 ug/mL (~55 uM)	0.21	Chemistry of Natural Compounds. 52(6), 1089–1092 (2016)
	50 ug/mL (~111 uM)	0.59	
	10 uM	69	International Immunopharmacology. 59, 113–119 (2018)
**quercetin-3-O-rhamnoside (quercitrin)**	10uM	51	Evidence-Based Complementary and Alternative Medicine. 2020, Article ID 4307161 (2020)
	30 uM	56	

9. Vehicle control in Fig. 4 ROS is very unstable.

The intracellular ROS of RBL-2H3 treated with the isolated compounds, the vehicle control or the unstimulated control were determined in the same experiment, using a microplate reader to measure the signal at 30-s interval for 16 min. In Fig 5a (lower panel), all tested compounds and the vehicle control gave unstable signals in the same manner. However, relatively, we could still see that the isolated compounds suppressed the intracellular ROS generation, as compared to the vehicle control.

10. Ketotifen at 100 (ug/mL or uM?) should not be used if was toxic by over 20% in Table 1. And why it's values differ so much from Fig. 5 and 6 (35 vs 60 for 100ug/ml or uM?). The concentration unit is also unclear?

Ketotifen fumarate in Table1 was in the unit of µg/mL, while in µM unit in Fig 5 and Fig 6. So, the concentration of ketotifen fumarate in Table 1 and Fig 5 were different. As already explained in question#2, the reason that we used Ketotifen fumarate in the indicated units was to compare the activity with our tested samples in the same units.

In table 1, ketotifen fumarate 100 ug/mL(~235 uM): 37% b-hex release ratio (%of max)

In Fig 5, ketotifen fumarate 100 uM : 55% b-hex release ratio (%of max)

11. Why MS2 in mzcloud give sometimes no hit, while often many hits, which fragment (product) ion was considered?

All peaks in the MS2 spectra obtained from the experiment were used to match the available MS2 spectra on mzCloud database. The mzCloud database stores MS2 spectra acquired on Thermo Fisher Scientific mass spectrometers which provide similar MS2 pattern for the same compound. It is noted that LCMS data from similar instrument model generally gives comparable pattern.

Hit result refers to the spectra matching that pass the setting criteria (Precursor mass tolerance is less than or equal to 10 ppm, fragmentation energy tolerance is less than or equal to 20, Matching threshold is less than or equal to 75). The matching considered both the intensity and m/z accuracy of the fragment ions.

Therefore, "Hit/Not hit" depends on 2 major reasons

1. Availability of MS2 spectra: the identified compounds have MS2 spectra available on database that can be searched for matching.
2. The searching criteria: non-stringent setting criteria can increase the number of hits but will at the same time lead to higher chance of incorrect identification. The authors therefore strict to the setting indicated in the manuscript although some features give no hit.

12. Please explain abbreviations: What does NSS, ICR mean.

NSS stands for "normal saline solution"

ICR stands for "Institute of Cancer Research". ICR mice is a strain of albino mice originated in SWISS and have been sent to various places by the Institute of Cancer Research in the USA. This is a general-purpose model used for studies in a wide range of fields including toxicity, pharmacology, drug efficacy, and immunology.

13. Figure 1 The description of in vivo results may be clarified. The fact that the high concentration of extract was not inhibiting PCA in the same manner as control dexamethasone (also because the concentration used was different). Even the top concentration of extract did not statistically inhibit PCA. Is it due to a statistical error or is similar concentration effect observed in vitro as well? The cause may be too short pre-treatment in vivo with only a single dose??

The concentrations used in the *in vivo* study were dramatically higher than in the *in vitro* experiments. In our experiments, the mice were fed with 250 µL of the extract solutions at 1.4, 14 and 140 mg/mL for the 10, 100 and 1000 mg/kg groups, respectively. The passive cutaneous anaphylaxis (PCA) assay was performed according to the published standard protocol that was used to study the effects of other extract formulations or active compounds. And, the treatment doses were used according to previous studies and standard considerations, which inquired into treatment safety.

Unlike what we observed from the *in vitro* study (in Table1), we did not observe dose-dependent effect of the crude ethanol extract in the passive cutaneous anaphylaxis (PCA) reaction assay. **This could be due to an issue of pharmacokinetics in the *in vivo* model that is obviously different from in the *in vitro* model. Hence, further study of the extract in terms of molecular mechanism and pharmacokinetics will be important for optimizing the treatment doses and conditions**.

14. Table 1. The statistics is not reasonable. Stars are missing for ketotifen at 100 and crude or water at 2000 ug/mL. It is not reasonable to evaluate the effects of the samples toxic to the cells, such as when the cell death is over 20%, especially for butanol layer. Also the concentrations used were extremely high. Is there a reasoning for it? It would be useful to see thte IC50 values for clarification in the text, because the current description is very brief and often skips the inconsistent results.

As stated in question#2, **the activity results of the samples that were over 20% toxic were removed from Table 1. And, the discussion was also corrected accordingly**. We performed the test at 500, 1000 and 2000 µg/mL because we do not observe the significant difference in term of the activity at lower concentrations.

The statistical analysis was double checked and corrected.

15. Is the beta-hexo release ratio percentage of max, meaning max is triton or inducer alone?

The values were calculated according to the following equations.

β-hexosaminidase released ratio = A405 (supernatant)/ [A405 (supernatant) + A405 (cell lysate)]

β-hexosaminidase release ratio (% of max) = [β-hexosaminidase released ratio (sample)/ β-hexosaminidase released ratio (vehicle control)] x 100

So, the “max” was the vehicle control, of which the experiment was performed by

1. sensitizing the cells with IgE.
2. treating with DMSO (instead of the extracts or the isolated compounds) in Siraganian buffer.
3. stimulating the cells with the antigen.

16. What is AGC and its value of $1x10^5$?

The AGC target value or automatic gain control target value is an MS parameter specifying the ion population in mass analyser. AGC target setting value for orbitrap mass analyser of Tribrid Fusion mass spectrometer is approximately between $1x10^5$-$2x10^5$. Different AGC target setting will lead to different time use for data acquisition event in each MS, MS/MS cycle and number of obtained MS/MS data. **The author therefore indicated the value in the materials and methods section of the manuscript**.

17. How authors could use 1 g of EA fraction when only 0.9 g was obtained?

We performed two batches of extraction; 200g- and 100g-scales. The obtained yields are shown in the following table.

Experiment	Fractions	200g-scale		100g-scale	
		gram	%yield	gram	%yield
maceration	Plant powder	200		100	
	crude (EtOH)	26.29	13.1	14.72	14.7
solvent extraction	crude EtOH taken for partition	24.5		14.7	
	EtOH/Hex	11.2	45.7	7.5	51.0
	**EtOH/EtOAc**	**0.9**	3.7	**0.6**	4.1
	EtOH/BuOH	2.6	10.6	1.3	8.8
	EtOH/H2O	4.6	18.8	2.9	19.7
	total	19.3	78.8	12.3	83.7

After the extractions, we combined the same fractions from the two batches together before moving forward to the experiments reported in the manuscript. So, we obtained 1.5 grams of the EtOH/EtOAc fraction in total (0.9 grams +0.6 grams).

18. P. 13. Line 18 The statement "The parameters were adjusted to fit to the chromatographic data obtained from this experiment" it's not clear, how the parameters were adjusted and if in objective manner?

The parameters used in Compound Discoverer software were selected to fit the chromatographic data obtained from this experiment. For example, Orbitrap Fusion mass spectrometer is high accurate mass measurement. Therefore, only data with less than 5 ppm mass-tolerance was included for analysis. RT tolerance was set as 0.2-min upon average RT difference in each LCMS injection. The value was obtained from manually checked the elution time of chromatographic peaks in raw data.

The statement in Page15, lines 9-10, was changed to be "The parameters in CD3.1 for data analysis were set to be compatible with chromatographic data obtained from this experiment.".

19. Do the features represent the mass?

Feature represents molecular weight at specific retention time. For example, Feature: 516.12723_3.168 refers to a peak that was detected as molecular weight of 516.12723 and eluted at retention time of 3.168 minutes.

20. How the authors ensure that the isolated compounds were matching the metabolomics study?

The authors compared orthogonal data (retention time and MS, MS/MS at 2 different fragmentation energy) of isolated compounds and data of crude extracts used for metabolomics study as shown in the **S1 Appendix** and the data were in good agreement.

The isolated compounds were injected to LC-MS/MS under the same condition used for crude extract analysis. The peak of each purified compound was matched well to metabolomics study (peaks obtained from F3:EtOH/EtOAc) with low difference in retention time (the range of 0.00-0.02 min) and low mass error within ± 3 ppm as shown in **S1 Appendix** (Comparison of MS and MS/MS data of the features obtained from metabolite profiling to those of isolated compounds).

21. Except the in-house mass list, was there another database available and used for MS2 except mzcloud which often gave no result?

There are other MS2 databases to aid compound identification eg. NIST mass spectrometer database (https://chemdata.nist.gov/). NIST contains approximately 1.3 million MS/MS spectra of approximately 31,000 compounds from GC-MS/MS and LC-MS/MS. However, the data were collected from various types of ionization, fragmentation mechanism, mass analyser and resolution power. Searching on the database may lead to fewer number of hits because the experimental MS/MS data was matched to the data from different types of MS acquisition.

NIST 20 Tandem Mass Spectral Libraries

NIST 20 Flyer

- **30,999 compounds** doubled over NIST 17!
- **1,320,389 tandem spectra** doubled over a range of fragmentation conditions including positive an negative modes from ion-trap (up to MS^4) and 'beam-type' collision cells (QTOF, HCD, and QqQ); spectra acquired at high accuracy and resolution
- **185,608 precursor ions** including in-source fragments

Library	Instrument Type	Compounds	Precursor Ions	Spectra
**Small Molecule HRAM[1] MS/MS Library (hr_msms_nist)**	Beam Type Collision Cell	27,623	88,380	946,309
	Ion Trap	25,472	79,529	80,403
**Small Molecule Low Resolution MS/MS Library (lr_msms_nist)**	Beam Type Collision Cell	2,799	3,863	34,589
	Ion Trap	27,824	162,028	181,060
**Small Molecule APCI[2] High Resolution MS/MS Library (apci_msms_nist)**	Beam Type Collision Cell	246	605	3,555
**Biological Peptide MS/MS Library[3] (biopep_msms_nist)**	Beam Type Collision Cell	1,814	5,637	77,182
	Ion Trap	1,828	6,422	13,062
**All**	Beam Type Collision Cell	29,890	95,175	1,048,069
	Ion Trap	29,444	173,890	272,320
**Total**		30,999	185,608	1,320,389

[1] - HRAM: High Resolution Accurate Mass.
[2] - APCI: Atmospheric Pressure Chemical Ionization; for extractable and leachable compounds.
[3] - commercially available peptides; also contains small peptides from hr_msms_nist and lr_msms_nist.

22. Metabolomics Standard Initiative MSI levels 1-4 and the differences between them should be described. The resulting compounds identification such as four isolated compounds or apigenin should be given.

The authors have added the detail and differences of MSI level in the revised manuscript.

In the revised manuscript, Page17, line 25

“ Metabolomics Standard Initiative (MSI) level 1-4 were proposed as a minimal reporting standard for chemical analysis. MSI level 4 refers to unidentified or unclassified compounds that can be detected using spectral data. MSI level 3 includes compounds that can be putatively characterized or classified by using physicochemical properties or spectral similarity to a chemical class. MSI level 2 is for putatively annotated compounds with spectral similarity to known compounds in public/commercial spectral libraries. MSI level 1 is for novel and non-novel identified compounds. Novel MSI I metabolites can be characterized by elemental analysis, NMR, IR, UV and accurate mass measurement, while non-novel MSI I metabolite identification could be validated with a commercial standard compounds by comparison of a minimum of two independent and orthogonal data under identical experimental conditions.”

23. The effects of compounds on viability and hexosaminidase enzymatic activity should be mentioned clearly in the text (currently in Sup data), while negative values of EA layer should be explained, and methodology reviewed, -46.1 ± 0.9 (1000ug/mL) -89.5 ± 0.5 (2000 ug/mL)

In the revised manuscript, more detail about the effects of compounds on viability and hexosaminidase enzymatic activity is added to the discussion about the inhibitory activity of crude extracts on IgE-mediated mast cell degranulation.

In the revised manuscript, Page13, lines 11-14

“Moreover, to assure that the observed effects of the extracts on the inhibition of mast cell degranulation were not from the inhibition of the enzyme activity, β-hexosaminidase inhibitory activity of each extract was determined. The results showed that no significant inhibition was observed for all tested samples (see S3 Table).”

For determination of β-hexosaminidase inhibitory activity, RBL-2H3 7.5 x 10^6 cells were lysed with 15 mL of 1%triton-X 100 solution in Siraganian buffer. Then, aliquots of 40 μL of the supernatant containing β -hexosaminidase were incubated with 10 μL of the sample solutions in Siraganian buffer at indicated concentrations or DMSO (vehicle control) and 50 μL of the substrate solution. After incubation at 37˚C for 2 hours, 200 μL of the stop buffer was added and the absorbance was measured at 405 nm with a microplate reader. The experiment was performed in triplicate. The inhibitory activity was calculated according to the following equations.

$$\%\text{inhibition of } \beta\text{-hex activity} = [1- [A_{405} (\text{sample})/ A_{405} (\text{control})]] \times 100$$

Since the tested samples did not inhibit the activity of the enzyme, the absorbance values obtained from treatment with the tested sample and vehicle control were in the same range [Abs (sample) ≈ Abs (control)]. In case that Abs(sample) was even a little bit higher than Abs (control), the calculated value for %inhibition of β-hex activity would be negative.

24. P. 29. Line 8-9. The condition should be mentioned in Materials section. The further purification besides MPLC should be described and purity level estimated. The Fig. 3 MPLC chromatogram peaks are overlapping.

In the revised manuscript, the condition in Line 8-9 page 20 is removed. The further purification besides MPLC is described in the Material and methods section.

In the revised manuscript, Page8, lines 24-26

“The fraction with the highest anti-allergic activity (F3: EtOH/EtOAc) was further purified to obtain major chemical components for structural identification and activity validation. One gram of the F3: EtOH/EtOAc fraction was purified by using Medium Pressure Liquid Chromatography (MPLC; PuriFlash 450, Interchim, France) over reversed phase silica gel (PF-15C18AQ-F0080) and eluted with 3%-97% MeOH/H2O. **The resulting fractions were examined by thin layer chromatography (TLC). The fractions containing identical compounds were combined and concentrated under vacuum.** The NMR spectra of the isolated compounds were recorded on Bruker AVANCE NMR Spectrometer operating at 300 MHz for 1H and 75 MHz for 13C. High-resolution mass spectra were obtained using electron spray ionization (ESI) on Orbitrap Fusion™ Tribrid™ Mass Spectrometer (Thermo, Massachusetts, USA).”

The fractions obtained from MPLC were checked again by using thin-layer chromatography (TLC) so that only the pure fractions were collected. The yields reported in the manuscript were isolated yields. And from HPLC analysis, the purity of all the isolated compounds were at least 95%. The following are HPLC chromatograms of the four isolated compounds.

HPLC chromatograms of the isolated compounds

- *3,5-dicaffeoylquinic acid*

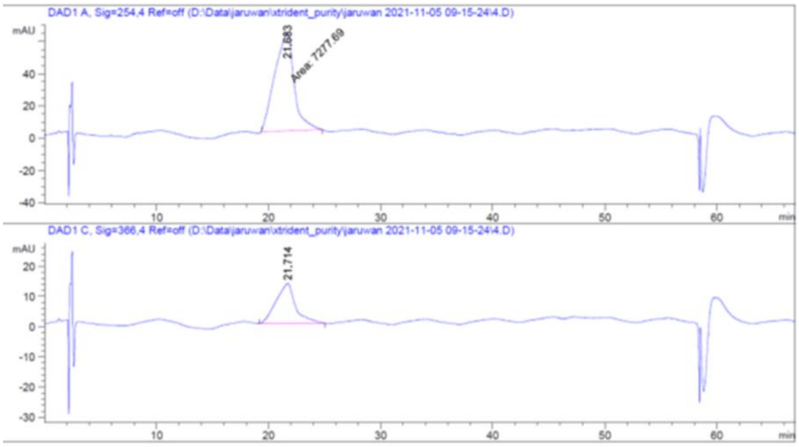

Signal 1: DAD1 A, Sig=254,4 Ref=off

Peak #	RetTime [min]	Type	Width [min]	Area [mAU*s]	Height [mAU]	Area %
1	21.683	MM	1.9970	7277.68945	60.73751	100.0000
Totals :				7277.68945	60.73751	

Signal 2: DAD1 C, Sig=366,4 Ref=off

Peak #	RetTime [min]	Type	Width [min]	Area [mAU*s]	Height [mAU]	Area %
1	21.714	BB	1.4561	1604.91162	13.27803	100.0000
Totals :				1604.91162	13.27803	

- *luteolin-7-O-glucoside*

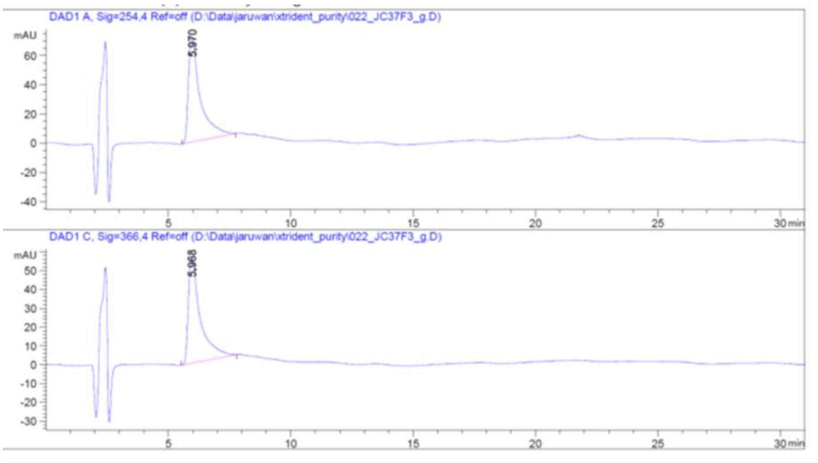

Signal 1: DAD1 A, Sig=254,4 Ref=off

Peak #	RetTime [min]	Type	Width [min]	Area [mAU*s]	Height [mAU]	Area %
1	5.970	BB	0.4821	2424.39087	71.77569	100.0000
Totals :				2424.39087	71.77569	

Signal 2: DAD1 C, Sig=366,4 Ref=off

Peak #	RetTime [min]	Type	Width [min]	Area [mAU*s]	Height [mAU]	Area %
1	5.968	BB	0.4866	1920.42749	56.21298	100.0000
Totals :				1920.42749	56.21298	

- *quercetin-3-O-rhamnoside*

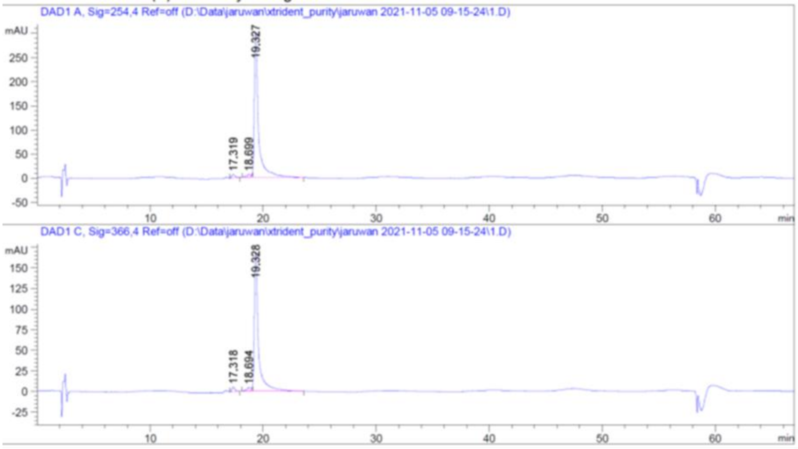

Signal 1: DAD1 A, Sig=254,4 Ref=off

Peak #	RetTime [min]	Type	Width [min]	Area [mAU*s]	Height [mAU]	Area %
1	17.319	BB	0.2633	138.09891	7.41467	1.7050
2	18.699	BV E	0.3372	142.05417	5.53922	1.7538
3	19.327	VB R	0.3717	7819.50537	300.43076	96.5412

Signal 2: DAD1 C, Sig=366,4 Ref=off

Peak #	RetTime [min]	Type	Width [min]	Area [mAU*s]	Height [mAU]	Area %
1	17.318	BB	0.2580	103.88760	5.66264	2.2848
2	18.694	BV E	0.3144	103.75903	4.37941	2.2820
3	19.328	VB R	0.3691	4339.29346	168.14775	95.4333

-
- *kaempferol-3-O-rhamnoside*

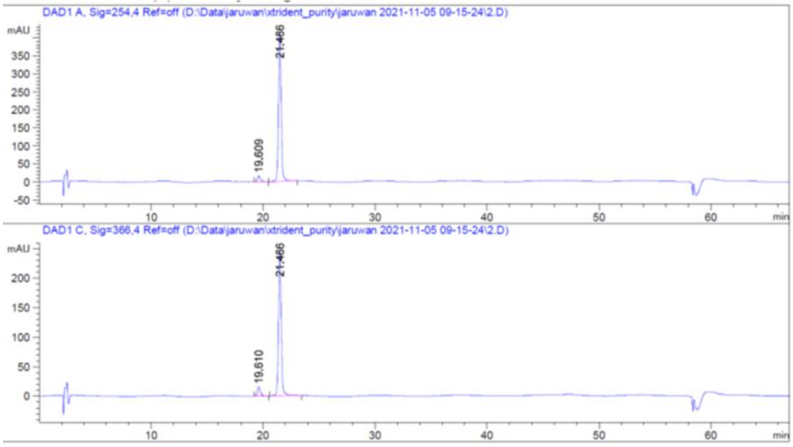

Signal 1: DAD1 A, Sig=254,4 Ref=off

Peak #	RetTime [min]	Type	Width [min]	Area [mAU*s]	Height [mAU]	Area %
1	19.609	BB	0.2855	317.31110	16.86901	4.3850
2	21.486	VB R	0.2623	6918.90869	413.10666	95.6150

Signal 2: DAD1 C, Sig=366,4 Ref=off

Peak #	RetTime [min]	Type	Width [min]	Area [mAU*s]	Height [mAU]	Area %
1	19.610	BB	0.2861	294.57797	15.61703	6.6367
2	21.486	BB	0.2647	4144.07324	245.77841	93.3633
Totals :				4438.65121	261.39544	

25. What represents the last four number of each “feature”?

The last four number of each feature is the “retention time”. For example, in feature 516.12723_3.168, the last four number 3.168 indicates retention time (RT) that metabolite with molecular weight of 516.12723 dalton was eluted from the chromatographic column.

26. Please unify a reference style, there are missed capital for Genus name or the style is inconsistent. Capitalization, please unify

In the revised manuscript, the references are re-checked and unified.

27. LC-MS/MS in S1 show only few peaks, on which basis they were identified and what could be the major peak at 5.17?

LC-MS in **S1 Appendix** (LC-MS Metabolite profiling) is base peak ion chromatogram showing relative abundance of peaks presented in each sample. The major peak at 5.17 min (5.15-5.22 min) was predicted to be a compound with molecular weight of 324.21996 and chemical formula of C21H28N2O. For compound identification, MS2 showed no matching to any spectrum on mzCloud database. Based on MS1 data, this major peak at 5.17 min could be predicted with the lowest mass error of -0.64 ppm to several compounds including 4,4'-bis(Diethylamino)benzophenone, 1-(9H-Carbazol-9-yl)-3-(dipropylamino)-2-propanol, N-[4-(Diethylamino)phenyl]-4-(2-methyl-2-propanyl)benzamide, diampromide, and N2-Butyl-N-(1,2-diphenylethyl)-N2-methylglycin-amide.

There are many syntax and grammar errors or misspellings, please see following as examples only:

P. 6 l. 12 by intraperitoneal administration

What does NSS, ICR mean.

The grammatical error is corrected.

The meaning of NSS and ICR are described in the above comment (question#12).

P13, l. 3 screening was

The grammatical error is corrected.

P.14, l. 9 discrimination possibly be attributed to

The grammatical error is corrected.

P.3 lines 21-25 rephrase please. Statistic treatment,

The sentences are rephrased.

P. 4 knowledges.

The grammatical error is corrected.

P. 4 Line 13 contributed

The grammatical error is corrected.

P. 21. Line 6 bioassay-guided is not so clear

The word “bioassay-guided isolation” is removed.

P. 13. Line 16 detect

The word “Detect unknown compound” is the name of step used in CD3.1analysis software. These are also for “Align retention time”, “Group unknown compound” **To make it clear for readers, they are changed to “Unknown compound detection”, “Retention time alignment” and “Unknown compound grouping”**.

P. 14 Unsupervised analysis could be explained. Figure 2 is not very clear.

Unsupervised PCA analysis is explained in the revised manuscript.

P. 15. Line 1-2 rephrase please.

The sentences are rephrased.

P. 29 cpd 24 name

The typo is corrected.

Conclusion. Please specify statements: P. 23. Line 2 “Some proinflammatory factors” is too vague

The wording is changed.

Line 8-10 please rephrase

The sentences are rephrased.

Line12-15 “to its highly abundance” rephrase and consider about role of hexane layer

The wording is changed.

Line14-16 please rephrase

The sentences are rephrased.

PCA (same for in vivo experiment) abbreviation should not be used for “Fig 2. Principle component analysis (PCA) and Heat map visualization of X.tridentata extracts.”

To avoid the confusion, we use the full name for the “passive cutaneous anaphylactic” in the revised manuscript. Therefore, **“PCA” present in the manuscript only stands for “Principle component analysis”.**

Also, the analysis and discrimination of layers is not clear.

The authors have modified this paragraph in the revised manuscript.

<u>Additional corrections</u>.

- The legend for Fig4 is corrected.
- “The Center of Excellence on Environmental Health and Toxicology, Commission on Higher Education, Ministry of Education, Bangkok, Thailand” is added as another affiliation for Jaruwan Chatwichien.
- More detail is added in the Acknowledgements section.
