# Supplementary material for: LC-MS/MS metabolomics-facilitated identification of the active compounds responsible for anti-allergic activity of the ethanol extract of Xenostegia tridentata
Source: PLoS One. 2022 Apr 15;17(4):e0265505. doi: 10.1371/journal.pone.0265505 (PMC9012362; doi:10.1371/journal.pone.0265505)
Supplement: Attachment — Submitted filename: Response to Reviewers.pdf [file pone.0265505.s007.pdf]

Response to reviewers

[PONE-D-21-29347R1] - [EMID:c1cfdba533d70f83]

LC-MS/MS metabolomics-facilitated identification of the active compounds responsible for anti-allergic activity of the ethanol extract of *Xenostegia tridentata*

Author's response to Journal Requirements:

Please review your reference list to ensure that it is complete and correct. If you have cited papers that have been retracted, please include the rationale for doing so in the manuscript text, or remove these references and replace them with relevant current references. Any changes to the reference list should be mentioned in the rebuttal letter that accompanies your revised manuscript. If you need to cite a retracted article, indicate the article's retracted status in the References list and also include a citation and full reference for the retraction notice.

The reference list is carefully reviewed. The following changes are made.

1. The references 28 and 33 are replaced with other relevant papers.
2. DOI of reference 23 is corrected.
3. The titles are displayed in sentence-format (capitalize the first letter of the first word).
4. The author's names and journal abbreviations are re-checked and corrected.

Author's responses to reviewer's comments:

1. The role of LC-MS/MS metabolomics in current study may be exaggerated since the methods failed to identify the major compounds in the first place.

In this work, LC-MS/MS metabolomics facilitated the identification of several potential anti-allergic compounds in *X. Tridentata* without prior purification step. The technique was successfully used to identify three major compounds (luteolin-7-O-glucoside, quercetin-3-O-rhamnoside and kaempferol-3-O-rhamnoside) that were later confirmed their identities by MPLC separation-NMR structural elucidation. Although LC-MS/MS was not able to ensure the specific isomer of feature 516.12723_3.168, the class of this feature could be identified as dicaffeoylquinic acid. It was also noted that LC-MS/MS metabolomics technique could identify other compounds (Apigenin, (2E)-3-(4-Hydroxyphenyl)-N-[2-(4-hydroxyphenyl)ethyl]acrylamide, Apigetrin (Apigenin-7-O-glucoside)) that could not be obtained from MPLC separation.

2. The PC 1 and PC 2 descriptors were not explained in Fig. 2.

PC1 and PC2 are explained in the main text.

In the 2nd revised manuscript, Page 15, lines 19-22,

"Principal component 1 (PC1) represents the maximal variation of data, whereas principal component 2 (PC2) is orthogonal to the PC1 axis and accounts for the second highest variation. Herein, two-component PCA models accounted for the total variance of 71.1% and 72.9% for ESI positive and negative, respectively."

3. Dicaffeoylquinic was identified as 4,5 by MS/MS but 3,5 by isolation.

As explained on page 18, lines 21-27, from the MS/MS analysis, we could only putatively identify the feature 516.12723_3.168 as dicaffeoylquinic acid. Consequently, the isolation and NMR analysis were performed to confirm that the compound was 3,5-dicaffeoylquinic acid.

4. Information could be shared in Supporting Information:

- **The list of 116 collected samples related to this plant may have been shared in Supporting information or online database.**

  The author added the list of 116 compounds as a supplementary file “S4 Table. library for LC-MS”.

- **The collected literature data on previously reported bioactivity (Reply 8) could also be added to Supporting information, taken that authors spent time to analyze the data (Note. cynaroside % Inh values must be mistaken 0.21 and 0.59).**

  The data on previously reported anti-allergic activity of the isolated compounds is added to S3 Table. The %inhibition of cynaroside reported in the indicated reference is re-checked. 0.21 and 0.59 were the values taken from the paper. They were not mistaken.

- **The table with yields of extraction. (Reply 17)**

  The extraction yield is added to S3 Table.

- **The fact that vehicle control being activated by antigen in M&M (Reply 15)**

  The detail about vehicle control is added to S3 Table.

- **HPLC chromatograms of isolated compounds (Reply 24).**

  The HPLC chromatograms of isolated compounds are added to S2 Appendix.

- **The possible identity of major peak at 5.15-5.22 (could be mentioned in the main text).**

  The possible identity of this peak is added to the main text.

In the 2nd revised manuscript, Page 18, lines 3-12,

“LC-MS metabolite profiles of five extracts (F1-F5) in both ESI positive and negative (S1 Appendix) were subjected to metabolomics data analysis as previously described. The LC-MS profile is a base peak ion chromatogram showing the relative abundance of peaks presented in each sample. The major peak for five extracts analyzed in ESI positive mode at 5.15-5.22 min in S1 Appendix was predicted to be a compound with a molecular weight of 324.21996 and a chemical formula of $C_{21}H_{28}N_2O$. For compound identification, MS2 showed no match to any spectrum on mzCloud database. Based on MS1 data, this major peak at 5.15-5.22 min could be predicted with the lowest mass error of -0.64 ppm to several compounds including 4,4'-bis(Diethylamino)benzophenone, 1-(9H-Carbazol-9-yl)-3-(dipropylamino)-2-propanol, N-[4-(Diethylamino)phenyl]-4-(2-methyl-2-propanyl)benzamide, diampromide and N2-Butyl-N-(1,2-diphenylethyl)-N2-methylglycin-amide.”

5. Very nice and clear NMR analysis and literature comparison in Supporting Information. On which basis author confirmed the correct position of glucoside luteolin-7-O-glucoside when there is no HMBC correlation H1'' to C-7?

According to the literature[C], it's stated that "**the chemical shift value of H-1'' (δ_H 5.07) was typical of an attachment at C-7 position. The chemical shift value of H-1'' also confirmed that the β-glucose unit was connected to C-7 through an O-linkage**." Also, as shown in the below table, when compare the obtained proton and carbon NMR spectra to the spectra of different luteolin-glucosides reported in literatures, we found that our isolated compound corresponds to luteolin-7-O-glucoside.

position	δ_H (J in Hz)			
	experiment	luteolin-7-O-glucoside[a]	Luteolin-5-O-β-D-glucoside[b]	Luteolin-3'-O-β-glucopyranoside[c]
3	6.75 (1H,s)	6.73 (1H,s)	6.56 (1H, s)	6.54 (1H,s)
6	6.44 (1H, d, 2.1)	6.46 (1H, d, 2.0)	6.71 (1H, d, 2.0)	6.20 (1H, br, s)
8	6.78 (1H, d, 2.1)	6.81 (1H, d, 2.0)	6.81 (1H, d,2.0)	6.80 (1H, br, s)
2'	7.42 (1H, d, 2.3)	7.43 (1H, d, 2.0)	7.37 (1H, d, 1.5)	7.79 (1H, br, s)
5'	6.90 (1H, d, 8.3)	6.94 (1H, d, 8.2)	6.89 (1H, d, 8.0)	6.98 (1H, d, 7.5)
6'	7.45 (1H, dd, 8.3,2.3)	7.47 (1H, dd, 8.2, 2.0)	7.38 (1H, dd, 8.0, 1.5)	7.64 (1H, d, 6.0)
**1''**	**5.08 (1H, d, 7.1)**	**5.07 (1H, d, 7.5)**	**4.72 (1H, d, 7.5)**	**4.91 (1H, brs)**
	δ_C			
2	164.5	164.9	161.8	164.4
3	103.2	103.7	108.7	104.1
4	181.9	181.8	177.4	181.8
5	161.1	161.2	163.1	161.6
6	99.5	100	104.8	99.3
7	162.9	163.4	159.1	163.6
8	94.7	95.4	98.6	94.7
9	156.9	157.4	158.8	157.5
10	105.3	105.8	106.1	103.7
1'	121.4	121.9	122	122.3
2'	113.6	113.9	113.6	114.8
3'	145.8	146	146.1	145.9
4'	149.9	150.1	149.7	150.9
5'	116	116.4	116.4	116.8
6'	119.2	119.7	119	122.1
**1''**	**99.9**	**100.4**	**105**	**102.3**
2''	73.1	73.4	74.1	73.9
3''	77.2	77.5	78	76.5
4''	69.5	70	70.2	70.6
5''	76.4	76.6	76.1	77.9
6''	60.6	61	61.3	61.5

[a]Chem. Nat. Compd. *45*, 881–882 (2009)

[b]Food Chemistry *164*, 150–157 (2014)

[c]FABAD J. Pharm. Sci. *28*, 71-75 (2003)

6. Semi-quantification is based on AUC of detected mass. Could relative amount per g of plant extract be calculated?

Relative AUC levels of four compounds across five fractions were shown in Fig 4. To calculate the relative amount per gram of the plant extract, a standard compound with known amount could be injected to LC-MS/MS under the same condition. Observed AUC of varied amount of standard compound could be used to generate a standard curve for calculating relative amount per gram of plant extract.

7. Some English errors could be corrected:

p.17 l.13, l.16 was/were (*MSI 1)

The grammatical errors are corrected.

Supporting: diCaffeoyl, lutoelin-7-O-D-glucoside etc.

The typos in supporting material are corrected.

p.4 line 7 *considered as safe

The word “are safe” is replaced with “which are considered safe”.

p.11 l.18 *is possibly related

The article “was” is added in line 18, page 11.

Other corrections:

1. Since the name of affiliation #6 has recently changed (Jan 6, 2022), we have updated the name in the revised manuscript accordingly.

2. The file name “S4 Table” was changed to “S5 Table”.
