# Supplementary material for: LC-MS/MS metabolomics-facilitated identification of the active compounds responsible for anti-allergic activity of the ethanol extract of Xenostegia tridentata
Source: PLoS One. 2022 Apr 15;17(4):e0265505. doi: 10.1371/journal.pone.0265505 (PMC9012362; doi:10.1371/journal.pone.0265505)
Supplement: Attachment — Submitted filename: Responses to reviewers.pdf [file pone.0265505.s008.pdf]

PONE-D-21-29347R2

LC-MS/MS metabolomics-facilitated identification of the active compounds responsible for anti-allergic activity of the ethanol extract of *Xenostegia tridentata*

Author's responses to editor's comments

1. The authors did not fully address Reviewer's and Academic Editor's concerns. Specifically, PC1 and PC2 descriptors should be given in the caption for Figure 2, regardless of their introduction in the main text. Figure captions should stand alone and be readable without referencing to the main text.

The descriptions of PC1 and PC2 are added to the caption for Fig 2.

2. Regarding Reviewer's suggestion about expressing compound masses as relative amounts per gram, I am really convinced that the reviewer asked the authors to present such a calculation (as a table, presumably), not just to answer if this possibility exist.

(Reviewer's suggestion: Semi-quantification is based on AUC of detected mass. Could relative amount per g of plant extract be calculated?)

Regarding the reviewer's suggestion, we believe that the relative amount per gram of different compounds in the plant extracts **could not be <u>accurately</u> calculated** based on the AUC of the detected mass shown in our "semi-quantification" result. (*Noted that the semi-quantification analysis is the comparison of the same compound in different fractions*). This is because degree of ionization of different compounds can be different. (eg. 1 gram of compound A and 1 gram of compound B can give different AUCs.) To obtain a relative amount per gram of the plant extract, a standard curve needs to be obtained from external standard calibration plots, internal standard calibration plots, or standard addition method. In external standard calibration, a standard compound with a known amount could be injected into LC-MS/MS under the same condition. The observed AUC of different amount of a standard compound could be used to generate a standard curve to calculate the relative amount per gram of plant extract.

The following is our effort to estimate the relative amount of each compound, based on the currently available data. Herein, the purified peaks were considered as standard compounds in the calculation below.

Prior to the calculation, **we replaced the data of ESI-516.12723_3.168 (ESI negative) with ESI+516.12723_3.168 (ESI positive) in Figure 4**. We therefore would like to thank the editor and reviewers for this question, which prompted us to notice that it would be better to compare the data from the same ionization mode. Both the area under the curve of compound of interest in extracts ($AUC_{Extract}$) and the area under the curve of purified peaks ($AUC_{Compound}$) can be determined by using Qual Browser software, shown in Table 1 (presented in the revised Figure 4) and Table 2, respectively. Crude extracts and purified peaks were at the same amount of 0.1 mg per injection (1 mg/mL, 10 μL). The calculation was performed as shown in the steps below:

AUC of compound 0.1 mg = $AUC_{Compound}$

AUC of compound X mg in the crude extract = $AUC_{Extract}$

$$AUC_{Extract} = (X * AUC_{Compound})/0.1$$

X	= 0.1 * (AUC $_{Extract}$/ AUC $_{Compound}$)	
0.1 mg of extract contain compound of interest	X	mg
1 g of extract contain compound of interest	X * 1/ 0.1 g = X/0.1	g
1 g of extract contain compound of interest	0.1 * (AUC $_{Extract}$/ AUC $_{Compound}$)/0.1	g
1 g of extract contain compound of interest	AUC $_{Extract}$/ AUC $_{Compound}$	g

Therefore, the relative amount per gram of extract was determined using the equation below. The result is shown in Table 3.

Relative amount per gram of extract = AUC $_{Extract}$/ AUC $_{Compound}$

It should also be noted that this calculation used only one concentration of a purified peak and did not account for sample-to-sample variation or matrix effect. Therefore, the calculation is an estimation of relative amounts per gram. Moreover, to avoid misunderstanding the purpose of using the data as accurate comparison, **in the revised manuscript, we have replaced the word "semi-quantitation" with "relative metabolite level" in main text.**

Table 1. Area under the curve (AUC) of crude extracts

Feature		Average AUC of extract (AUC$_{Extract}$)				
		F1	F2	F3	F4	F5
		Cr.EtOH	EtOH/Hexane	EtOH/EtOAc	EtOH/BuOH	EtOH/H2O
ESI+	516.12723_3.168	12,634,842	1,717,453	68,026,912	13,540,138	189,293
ESI+	448.10082_3.034	34,035,133	7,107,225	153,130,350	41,472,219	106,948
ESI+	448.10108_3.251	35,406,011	14,333,418	171,914,293	5,196,971	50,831
ESI+	432.10612_3.604	5,226,637	2,194,514	36,449,731	199,786	n/d

Table 2. Average area under the curve (AUC) of purified peak 1-4

Compound	AUC			Average AUC of compound (AUC $_{Compound}$)
	LC1	LC2	LC3	
3,5-dicaffeoylquinic acid	160,259,536	153,864,153	163,936,249	159,353,313
luteolin-7-O-glucoside	420,844,486	428,113,381	413,038,103	420,665,323
quercetin-3-O-rhamnoside	705,867,873	790,660,894	707,709,687	734,746,151
kaempferol-3-O-rhamnoside	221,844,931	261,203,612	232,464,910	238,504,484

Table 3. Approximate relative amount per gram of crude extract calculated with only one concentration of purified compounds

Feature		AUC $_{Compound}$ from Table 1	Calculated relative amount (gram) per 1 gram of extract				
			F1	F2	F3	F4	F5
			Cr.EtOH	EtOH/Hexane	EtOH/EtOAc	EtOH/BuOH	EtOH/H2O
ESI+	516.12723_3.168	159,353,313	0.08	0.01	0.43	0.08	0.00
ESI+	448.10082_3.034	420,665,323	0.08	0.02	0.36	0.10	0.00

ESI+	448.10108_3.251	734,746,151	0.05	0.02	0.23	0.01	0.00
ESI+	432.10612_3.604	238,504,484	0.02	0.01	0.15	0.00	n/d

3. Please do not randomly capitalize words (compounds particularly), even if abbreviation is provided in parenthesis (e.g., P5L6, P5L7, P10L21, P10L23, P18L10-11, and further in the text).

The words throughout the manuscript are checked and corrected to not be randomly capitalized.

4. Citations in square brackets in the text should stand before a comma.

The citations in square brackets are moved to stand before commas and periods.

5. Please do not spell out "nanometers" (P7L15). Using "nm" only is quite acceptable. Also, using "AlCl3" without previous description is also perfectly fine (P7L14). Do not express centrifugation speed in rpm (P8L1). Use "g" instead (it needs computing), since equal rotation (rpm) in rotors with different diameters produce different g force. Therefore, g is a constant, while rpm is not. Do not use semicolons when listing items after colons, but use commas instead.

The words are corrected according to the editor’s suggestions.

6. Merremia tridentata is a synonym of the studied plant species and cannot be referred to as "other plants in the Convolvulaceae family" (P17L4). Moreover, using vernacular expressions such as "other plants" (might present other individuals) instead of "plant species" is not scientifically justified.

The word “in other plants” is replaced with “plant species”.
